# Unnatural Triggers Converted From Tetrazine‐Attached Sialic Acid for Activation of Optoacoustic Imaging‐Guided Cancer Theranostics

**DOI:** 10.1002/anie.202503850

**Published:** 2025-05-02

**Authors:** Yinglong Wu, Lihe Sun, Xiaodong Zhang, Wenbin Zhong, Xiaokai Chen, Shihuai Wang, Yun Chen, Dongdong Wang, Ting He, Hongzhong Chen, Jingjing Guo, Fang Zeng, Menghuan Li, Zhong Luo, Shuizhu Wu, Yanli Zhao

**Affiliations:** ^1^ School of Chemistry Chemical Engineering and Biotechnology Nanyang Technological University 21 Nanyang Link Singapore 637371 Singapore; ^2^ Biomedical Division State Key Laboratory of Luminescent Materials and Devices College of Materials Science and Engineering South China University of Technology 381 Wushan Road Guangzhou 510640 P.R. China; ^3^ School of Life Science Chongqing University Chongqing 400044 P.R. China

**Keywords:** Activatable systems, Cancer theranostics, Image‐guided therapy, Multispectral optoacoustic imaging, Unnatural targets

## Abstract

Constructing chemical groups on cell membranes through metabolic glycoengineering of unnatural sugars is an effective means to solve the issue of insufficient or even lack of targets in cancer theranostics. Herein, we address the limitations by developing a tetrazine precursor (SiaTz) based on a non*O*‐acetylated sialic acid scaffold and then utilizing it to create unnatural tetrazine triggers on the surface of cancer cells. SiaTz exhibits a good balance between the stability and reaction kinetics under physiological conditions and can be efficiently converted into corresponding tetrazine trigger through bypassing several size‐limiting steps in metabolic glycoengineering process. We also prepare a proof‐of‐concept theranostic combination of a *trans*‐cyclooctene derivative (CyTCO) and a thermal‐sensitive drug 2,2′‐azobis[2‐(2‐imidazolin‐2‐yl) propane]‐dihydrochloride (AIPH) to verify the activation function of tetrazine triggers in theranostics of orthotopic and metastatic tumors. In the presence of tetrazine triggers, CyTCO can be activated via bio‐orthogonal reaction to induce optoacoustic signal enhancement, enabling high‐contrast diagnostic imaging and precise tumor localization to guide subsequent treatments. Tetrazine trigger‐activated CyTCO displays high photo‐to‐heat conversion efficiency, which can cause an obvious increase in temperature under laser irradiation and then initiate AIPH decomposition to produce toxic radicals for combined therapy.

## Graphical abstract

Tetrazine precursors (SiaTz) based on a non*O*‐acetylated sialic acid scaffold are rationally designed and synthesized, which can be efficiently converted into unnatural tetrazine (Tz) triggers on cancer cell surfaces via metabolic glycoengineering. These Tz triggers activate theranostic agents for multispectral optoacoustic tomographic (MSOT) imaging, enabling localization and image‐guided therapy of orthotopic and metastatic tumors.

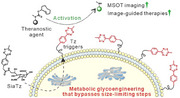

## Introduction

Cancer theranostics is an emerging biomedical technology that integrates cancer diagnostic imaging and cancer therapy, enabling early detection and precise treatment at the right time and appropriate dose.^[^ ^1^ ^]^ With a focus on patient‐centered care rather than adopting the “one size fits all” approach, this biotechnology is driving a shift from traditional medicine to a contemporary precision personalized medicine. To date, considerable endeavors have been devoted to developing various theranostic systems integrating different diagnostic imaging modalities (e.g., nuclear imaging and fluorescence imaging) and distinct treatment methods (e.g., chemotherapy and phototherapy).^[^ ^2^, ^3^, ^4^ ^]^ Regardless of the combination of treatment and imaging mode, the theranostic procedures basically involves two steps: accurate molecular imaging following efficient delivery of theranostic candidates into tumors via receptor‐mediated targeting, and subsequent image‐guided therapy.^[^ ^5^, ^6^ ^]^ Therefore, theranostic efficacy depends largely on whether cancer cells possess significantly more targetable receptors than normal cells, allowing the administered agents to be sufficiently enriched in the tumor tissues and minimizing the undesired adverse effects on healthy tissues. However, the expression of receptor targets varies greatly among different types of tumors (known as heterogeneity) and is even absent in some specific tumors,^[^ ^7^, ^8^, ^9^, ^10^ ^]^ which poses a huge challenge to the universal applicability of theranostic agents.

Over the past few decades, various chemical groups (e.g., azide, and alkyne) have been successfully conjugated to glycan backbones as unnatural targets to mimic the functions of endogenous receptors, thus overcoming the tumor heterogeneity.^[^ ^11^, ^12^ ^]^ Since these unnatural targets are expressed through intracellular metabolic glycoengineering of monosaccharide precursors, the desired chemical groups can be generated on the membrane by preconjugating the corresponding moieties to monosaccharides and then culturing cells with these modified precursors.^[^ ^13^ ^]^ Additionally, owing to the abnormal activity of enzymes (e.g., sialyltransferases and sialidases) related to metabolic glycoengineering in tumors, exogenous unnatural chemical targets can be presented at a higher number density on the membrane regardless of the cancer cell phenotype, which is highly beneficial for improving targeting efficiency.^[^ ^14^, ^15^ ^]^ Furthermore, the complementary functional groups in theranostic agents are able to interact with the unnatural targets on cell membranes in a bio‐orthogonal, biocompatible, highly specific, and efficient manner, thereby displaying lower immunogenicity in the host compared to protein antibodies directed against conventional endogenous receptors.

Currently, a commonly used unnatural chemical target is the azide group, which can specifically recognize extracellular alkyne‐containing imaging/therapeutic compounds via click chemistry and is typically produced by cellular metabolism of the per‐*O*‐acetylated mannose‐derived precursor.^[^ ^16^, ^17^, ^18^ ^]^ To further improve the expression efficiency, sialic acid analogs which are downstream molecules of mannoses in metabolic glycoengineering, have also been developed as precursors.^[^ ^14^ ^]^ Moreover, since the direct conversion from sialic acid precursors to unnatural targets bypasses several previous size‐limiting steps and only undergoes the final steps of metabolism, it also allows large‐sized chemical groups (e.g., dibenzocyclooctyne and bicyclononyne) to be incorporated into sialic acid and then rapidly generated on cell membranes.^[^ ^14^, ^19^ ^]^ Despite the recent advancements of theranostic systems employing unnatural azide targets, the rate constants of this emerging targeting strategy based on bio‐orthogonal reaction of dibenzocyclooctyne and azide are frequently below 1 M^−1^ s^−1^.^[^ ^20^ ^]^ Such a low reaction rate is very unfavorable for the administered theranostic agents to quickly reach the maximum concentration in the lesion to adequately exert their functions. Moreover, the targeting process is simply azide groups covalently attaching alkyne‐functionalized theranostic agents to the cell membrane surface, which cannot trigger the release of active imaging/therapeutic components, thus compromising the imaging contrast and therapeutic efficacy.^[^ ^21^ ^]^ In addition, recent studies have shown that while the *O*‐acetylation of monosaccharide precursors can improve cellular uptake, it may also lead to off‐target labelling and neurotoxicity due to their nonenzymatic reaction with abundant protein cysteine residues in living cells (known as artificial S‐glyco‐modification),^[^ ^22^, ^23^, ^24^, ^25^ ^]^ which prompts a shift in attention toward the development of non*O*‐acetylated monosaccharide‐based precursors for the construction of unnatural chemical targets. From these perspectives, several hurdles need to be cleared before unnatural chemical targets with triggering capabilities can reach their full potentials in wider preclinical and clinical applications.

Tetrazine ligation wherein a 1,2,4,5‐tetrazine (Tz) bio‐orthogonally reacts with an alkylene, has attracted considerable attention in biomedical applications due to its rapid and tunable reaction kinetics.^[^ ^26^ ^]^ Apart from forming covalent linkage with the complementary alkylene reagents, tetrazines can also activate the release of the substituents (e.g., imaging agents and drugs) from alkylene‐functionalized agents through a postbonding intramolecular elimination process.^[^ ^27^ ^]^ More importantly, tetrazine ligation can be performed specifically in a high yield under mild and aqueous conditions, which makes it possible to fabricate tetrazine targets on the cell surface as unnatural triggers for cancer theranostics.

When it comes to preparing precursors for unnatural tetrazine triggers, the stability of tetrazine groups under complex physiological environments needs to be thoroughly considered. In general, the tetrazine skeleton can be stabilized by incorporating electron‐donating groups to avoid hydrolysis and attack by biological nucleophiles.^[^ ^27^ ^]^ However, the introduction of electron‐rich substituents on the one hand reduces the reactivity of tetrazine toward its complementary alkenyl group, and on the other hand increases the size and steric hindrance of tetrazine. In view of these circumstances, it is necessary to address the issues of balancing reaction kinetics and stability while improving the conversion efficiency of large‐sized tetrazine precursors in the development of unnatural tetrazine triggers for selective activation of in vivo tumor theranostics.

**Figure 1:**
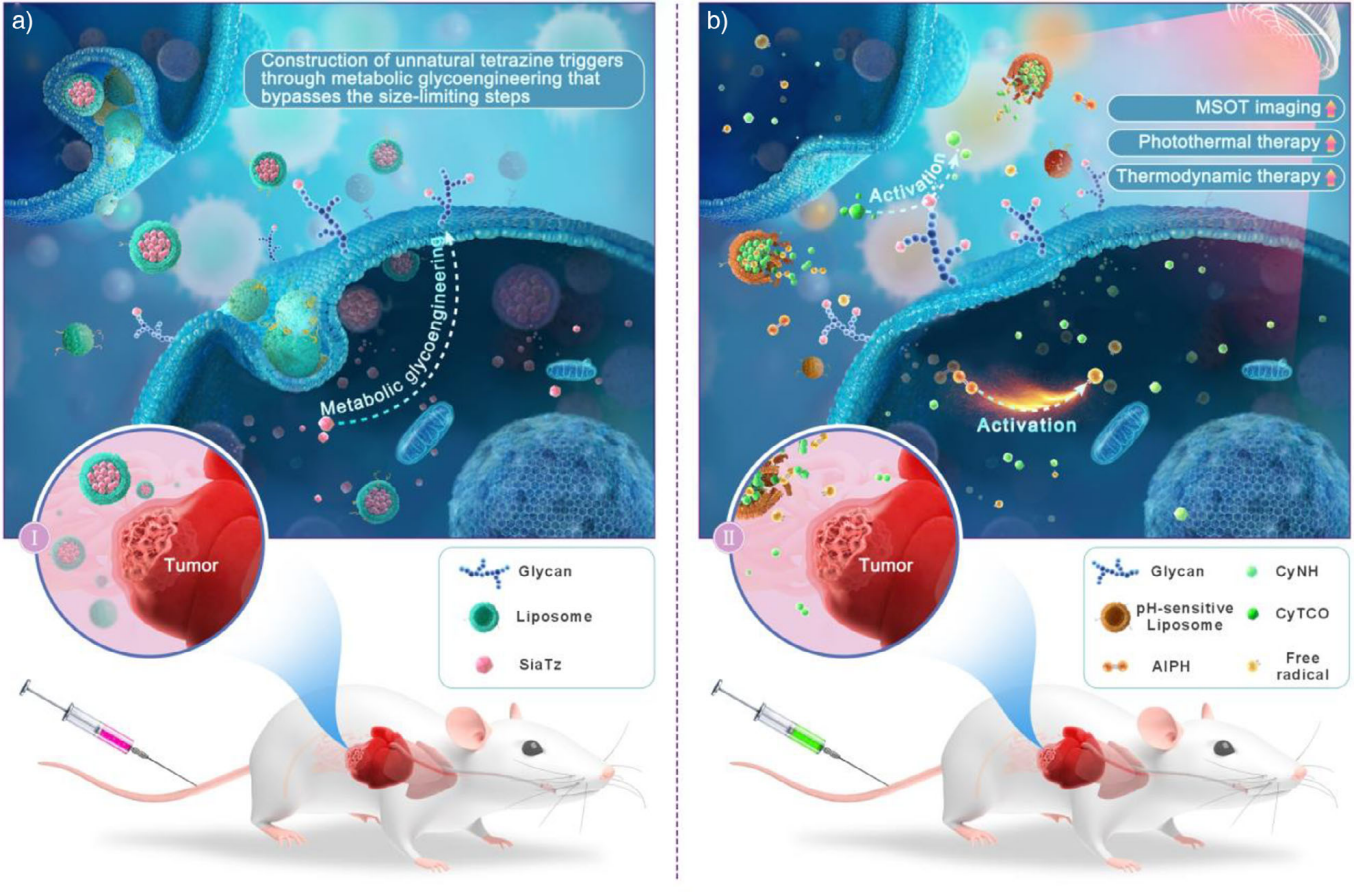
Schematic illustration. a) Construction of unnatural tetrazine triggers on cancer cell surface glycans via metabolic glycoengineering of the precursor SiaTz. The conventional liposomes are able to promote the accumulation of SiaTz in tumors via the EPR effect and help these hydrophilic precursors enter tumor cells. b) MSOT imaging‐guided synergistic photothermal/thermodynamic treatment of tumors activated by tetrazine triggers via bio‐orthogonal reaction. The pH‐sensitive liposomes can deliver the theranostic combination CyTCO/AIPH to tumor tissues, and rapidly release them in the acidic microenvironment, where they respond to the tetrazine triggers on cancer cell surfaces to produce active imaging/therapeutic agents (CyNH/free radicals), thereby allowing highly specific tumor theranostics.

In this study, we report an unnatural tetrazine trigger generated by metabolic glycoengineering of chemically modified sialic acid precursors on the surface of cancer cells, and demonstrate its utility in activating multispectral optoacoustic tomographic (MSOT) imaging‐guided photothermal/thermodynamic combination therapy of tumors in vivo via bio‐orthogonal reaction, as shown in Figure 1. The tetrazine precursor SiaTz is synthesized by the condensation of phenyl‐stabilized methyltetrazine and non*O*‐acetylated sialic acid and exhibits a high rate constant when reacting with *trans*‐cyclooctene (TCO) reagent in aqueous medium. After assembly with phospholipids, the precursors can be enriched in the tumor tissue via enhanced permeability and retention (EPR) effect, and efficiently converted into unnatural tetrazine triggers on the surface of cancer cells via metabolic glycoengineering (Figure 1a).

To verify the activation function of the tetrazine triggers expressed on the membranes, a proof‐of‐concept theranostic agent CyTCO is rationally designed by installing the complementary group TCO into the synthetic near‐infrared chromophore CyNH, and then loaded into pH‐sensitive liposomes together with a temperature‐sensitive drug 2,2′‐azobis[2‐(2‐imidazolin‐2‐yl) propane]‐dihydrochloride (AIPH). After the prepared pH‐sensitive liposomes accumulate and lyse in the acid tumor microenvironment, the released CyTCO can quickly bind to tetrazine triggers via bio‐orthogonal reaction and produce the active optoacoustic/photothermal agent CyNH through the subsequent bond cleavage. Using MSOT imaging, CyNH (the activated form of CyTCO) allows precise tumor localization and generation of orthogonal maximum intensity projection (MIP) images to guide phototherapy. Upon NIR laser irradiation, activated CyTCO can also rapidly transform the absorbed photon energy into heat for photothermal treatment (PTT) of cancer cells, while the elevated temperature can further trigger the decomposition of AIPH to yield free radicals for thermodynamic treatment (TDT) (Figure 1b). In this way, the tumor cells can be completely ablated via the tetrazine trigger‐mediated activation of synergistic PTT/TDT therapy.

## Results and Discussion

### Chemical Remodeling of Cancer Cell Surface Using Tetrazine Precursors

The precursor SiaTz (a tetrazine‐attached non*O*‐acetylated sialic acid compound) was synthesized according to the route shown in Figure S1. In the meanwhile, the control precursor ManTz based on extensively reported non*O*‐acetylated mannose was also synthesized. All the target products were well characterized by using ^1^H nuclear magnetic resonance (NMR), ^13^C NMR, and mass spectrometry (MS) (Figures S2–S13).

**Figure 2:**
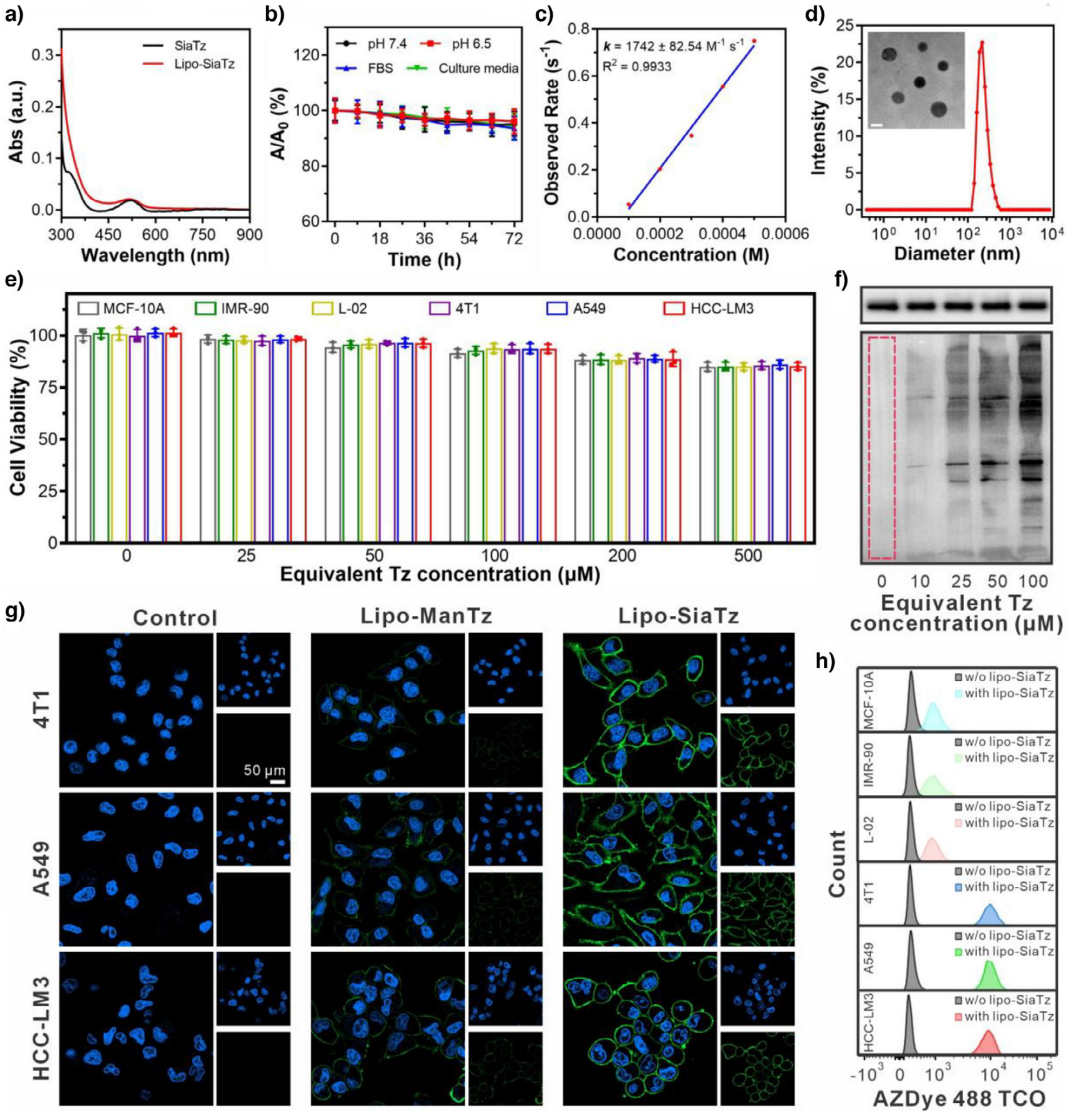
Generation of unnatural tetrazine triggers on cancer cell surface. a) Absorption spectra of SiaTz (10 µM) and Lipo‐SiaTz (equivalent Tz concentration: 10 µM). b) Absorbance ratio of SiaTz stored in pH 7.4 CPBS, pH 6.5 CPBS, FBS media, or cell culture media at room temperature for different times. c) Observed rate as a function of added TCO concentration. d) Size distribution and typical TEM image (inset) of Lipo‐SiaTz. Scale bar: 200 nm. e) Viabilities of various cells upon 48 h of incubation with Lipo‐SiaTz at different concentrations (*n* = 3 independent experiments). Data were represented as mean values ± standard deviation. f) Western blot images of 4T1 cells 48 h after administration with different concentrations of Lipo‐SiaTz. GAPDH (upper panel) was selected as the internal reference. g) CLSM images of cancer cells pretreated with Lipo‐SiaTz or Lipo‐ManTz (equivalent Tz concentration: 50 µM) and then labeled with AZDye 488 TCO. h) Flow cytometry data of tetrazine group expression in various normal (MCF‐10A, IMR‐90, and L‐02) and cancer (4T1, A549, and HCC‐LM3) cells after incubation with Lipo‐SiaTz. AZDye 488 TCO was used to specifically label the tetrazine groups generated on cell surface.

Next, the reaction kinetics and stability of the tetrazine groups in the synthetic precursors under biomimetic conditions were investigated. As shown in Figures 2a,b and S14, both SiaTz and ManTz exhibited a characteristic absorption peak at 520 nm and displayed considerable stability within 72 h in physiological environments, which is quite promising for expression of unnatural triggers on cell surfaces and biological applications. Upon addition of TCO‐containing compounds, the absorbance of SiaTz at 520 nm decreased sharply, indicating its reaction with the TCO groups (Figure S15). The second‐order rate constant of this bio‐orthogonal tetrazine‐TCO reaction was determined as 1742 ± 82.54 M^−1^ s^−1^, much higher than those of conventional couplings (e.g., 0.31–0.96 M^−1^ s^−1^ for azide‐DBCO reactions, and 0.0004–3.34 M^−1^ s^−1^ for cyclopropane‐tetrazine reactions),^[^ ^11^ ^]^ ensuring a rapid response at low concentrations under physiological conditions (Figure 2c and Table S1). To improve bioavailability and cellular uptake, SiaTz was encapsulated into phospholipid liposomes (Lipo‐SiaTz), which were spherical (236 nm) and quite stable (Figures 2d and S14c). Similarly, Lipo‐ManTz showed properties comparable to those of Lipo‐SiaTz (Figure S14 and Table S2). As shown in Figures 2e and S14, both Lipo‐SiaTz and Lipo‐ManTz presented low cytotoxicity even at a tetrazine equivalent concentration of 500 µM, suggesting their biocompatibility for construction of unnatural chemical triggers.

The conversion of SiaTz into tetrazine triggers via metabolic glycoengineering in cells was analyzed by western blots, indicating dose‐dependent expression of tetrazine triggers (Figure 2f). Confocal laser scanning microscopy (CLSM) images in Figure 2g revealed the tetrazine triggers were generated and distributed on the cell surfaces within 48 h. It has been reported that chemical modification of mannose precursors with larger than five carbon atoms or branched structures hinders the enzymatic conversion in metabolic glycoengineering, while sialic acid precursors, as the downstream molecules of mannose, can bypass those size‐limiting metabolic processes and be easily converted into unnatural chemical targets.^[^ ^14^ ^]^ Therefore, despite bearing a large‐sized phenyl‐stabilized tetrazine group, SiaTz could still produce a higher density of tetrazine triggers on the cell surfaces, as compared to ManTz (Figure 2g).

In addition, it was found from the flow cytometric data in Figure 2h and western blot results in Figure S16 that, the expression level of tetrazine triggers in cancerous cells (4T1, A549, and HCC‐LM3) was about 7 times higher than that in noncancerous cells (MCF‐10A, IMR‐90, and L‐02). This difference highlights the strong potential of tetrazine triggers as effective targets for cancer theranostics, as a 5‐ to 6‐fold increase in receptor expression is typically sufficient for tumor‐targeted theranostics (e.g., 5‐fold for transferrin receptor in certain ovarian cancer, and 6.5‐fold for HER2 in certain breast cancers).^[^ ^28^, ^29^ ^]^ More importantly, the tetrazine triggers presented high stability on cancer cell surfaces, supporting effective activation of theranostic agents (Figure S16c). These findings lucidly demonstrated the efficient construction of tetrazine triggers on tumor cell surfaces through metabolic glycoengineering of SiaTz, underscoring its promise for cancer theranostics.

### Utilization of SiaTz or its Converted Unnatural Triggers for Activation of Theranostic Agents in Solutions and Cells

**Figure 3:**
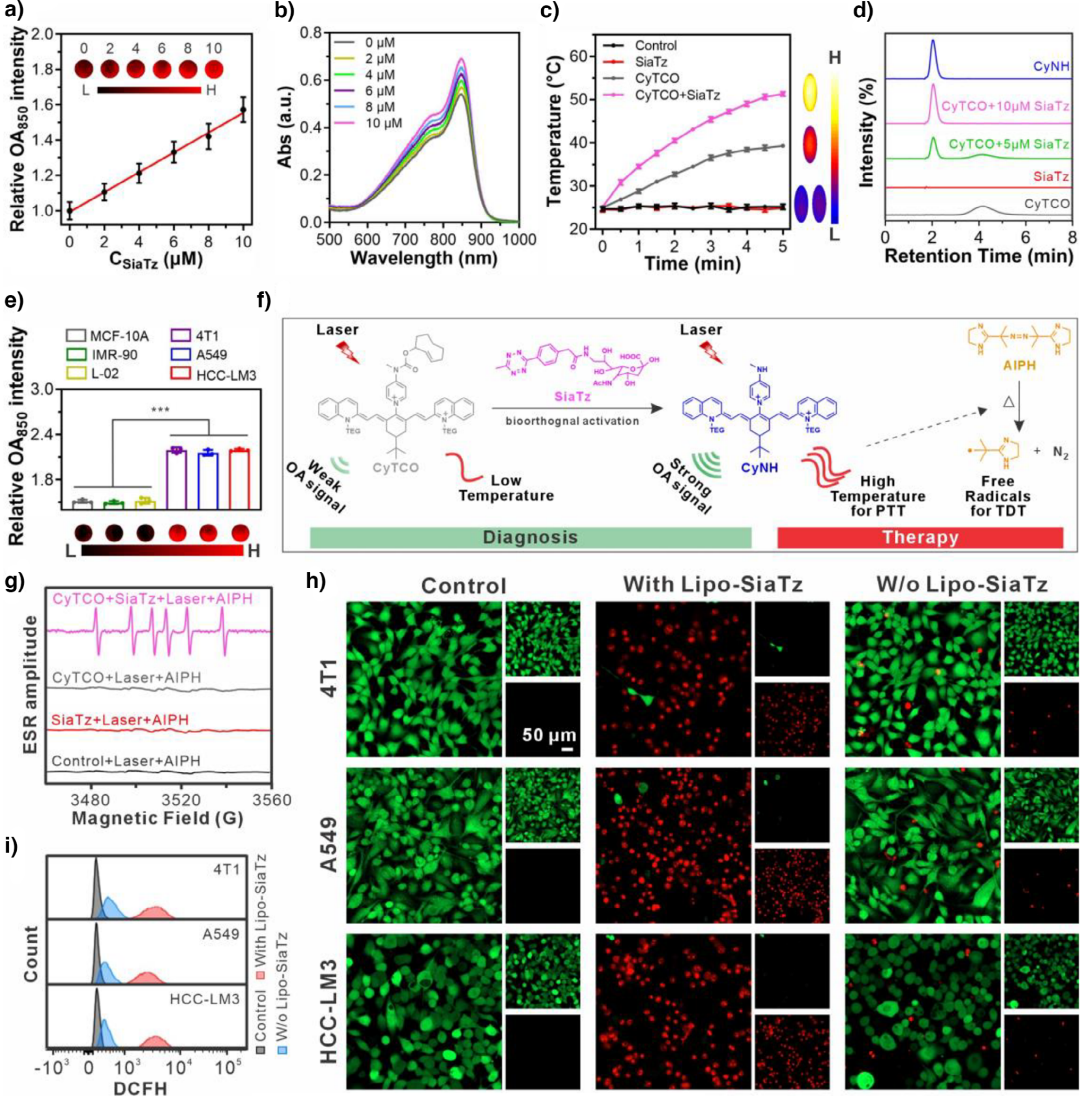
Use of the tetrazine precursor SiaTz or its converted tetrazine triggers for activation of theranostic agents in solutions and cells. a) Relative optoacoustic (OA) intensity of CyTCO (10 µM) after mixing with different concentrations of SiaTz in phantom and their corresponding OA images. b) Absorption spectra for CyTCO (10 µM) after mixing with different concentrations of SiaTz. c) Photothermal conversion behavior of pure CPBS (control), CyTCO alone, SiaTz alone, and the reaction mixture of CyTCO and SiaTz under NIR laser irradiation at 1 W cm^−2^ and the corresponding thermal images of those solutions after 5 mins of irradiation. d) HPLC traces for pure CyTCO, pure SiaTz, the reaction mixture of CyTCO and SiaTz (molar ratio: 1/0.5), and the reaction mixture of CyTCO and SiaTz (molar ratio: 1/1) and pure CyNH (the tetrazine‐activated form of CyTCO) in 850 nm channel. e) Relative OA intensities of various Lipo‐SiaTz‐pretreated cells incubated with CyTCO (10 µM) for 60 min in phantom (*n* = 3 independent experiments) as well as their corresponding optoacoustic images. Data were represented as mean values ± standard deviation. f) Illustration of SiaTz‐mediated CyTCO activation for MSOT imaging‐based diagnosis, photothermal therapy (PTT) and subsequent thermodynamic therapy (TDT) via AIPH cleavage induced by heat from activated CyTCO. g) ESR spectra of AIPH in the following solutions: CPBS (control), SiaTz, CyTCO, and the reaction mixture of SiaTz and CyTCO under NIR laser irradiation (1 W cm^−2^, 5 min). Free radicals from AIPH cleavage were captured by DMPO. h) Calcein‐AM/PI staining CLSM images and i) flow cytometric analysis on the intracellular free radical levels of cancer cells pretreated with or without Lipo‐SiaTz, following CyTCO/AIPH treatment and laser irradiation. For the control group, the cancer cells received no treatments. Statistical significance was determined by two‐tailed t test. ^***^ *p* < 0.001.

To confirm the capacity of the tetrazine group in SiaTz and the converted unnatural triggers to initiate the release of active theranostic candidates via bio‐orthogonal click reaction, as a proof of concept, an optoacoustic/photothermal bifunctional chromophore CyNH was synthesized and then caged by a TCO group to form the activatable theranostic agent CyTCO. The chemical structures of CyTCO and CyNH were characterized by ^1^H NMR, ^13^C NMR, and MS (Figures S17–S25). Then, the activation capability of the tetrazine groups in SiaTz toward CyTCO was investigated through measuring the changes in optoacoustic (OA) properties in phantoms. As exhibited in Figures 3a and S26, after SiaTz addition, CyTCO exhibited a maximum OA signal at 850 nm, with intensity correlating linearly to SiaTz concentration. It is worth noting that CyTCO itself showed good chemical stability (Figure S27a), thus confirming that the optoacoustic signal enhancement of CyTCO was induced by SiaTz. Moreover, this SiaTz‐triggered CyTCO activation was highly specific and unaffected by other biological substances (Figure S27b).

Given that optoacoustic effect is dependent on the transformation efficiency from light absorption to heat release and further to acoustic signal generation, the optoacoustic behavior of a certain chromophore strongly relies on its light‐harvesting capacity as well as excited‐state deactivation processes.^[^ ^30^, ^31^, ^32^ ^]^ As evinced by the results in Figures 3a–c and S27c,d, the enhancement of CyTCO optoacoustic signal after the addition of SiaTz can be ascribed to the following reasons: first, the increase in absorption is beneficial to the uptake of light energy; second, the intensified nonradiative decay helps to yield more heat for optoacoustic signal amplification.

Beyond photophysical analysis, the chemical activation of CyTCO by SiaTz was also examined. The high‐performance liquid chromatography (HPLC) data in Figure 3d showed CyTCO's peak at 4.2 min, which disappeared upon SiaTz addition, forming a new peak at 2.0 min corresponding to CyNH, as confirmed by MS (Figure S27e). These findings further verified that SiaTz can induce the release of active optoacoustic imaging agent CyNH from caged CyTCO via the bio‐orthogonal click reaction, as proposed in the inset of Figure S27e.

After demonstrating the SiaTz's activation function, its converted tetrazine triggers on the membranes were evaluated for CyTCO activation in optoacoustic imaging and phototherapy of cancer cells. The tetrazine triggers were constructed on the cell surfaces through pretreating the cells with Lipo‐SiaTz for 48 h. Obviously, upon CyTCO addition, SiaTz‐pretreated cancerous (4T1, A549, and HCC‐LM3) cells exhibited markedly stronger optoacoustic signal than SiaTz‐pretreated noncancerous (MCF‐10A, IMR‐90, and L‐02) cells (Figure 3e), highlighting its potential for precise cancer diagnosis and tumor localization in vivo.

To enhance therapeutic effects of activated CyTCO, a thermo‐sensitive drug AIPH, which decomposes above 44 °C to release alkyl free radicals for thermodynamic therapy,^[^ ^33^, ^34^ ^]^ was selected for an activatable cascade thermodynamic treatment (Figure 3f). Electron spin resonance (ESR) spectroscopy results (Figure 3g) confirmed free radical generation only in the CyTCO‐SiaTz mixture under NIR laser irradiation, as activated CyTCO efficiently converted light to heat, surpassing AIPH's pyrolysis threshold.

The ability of tetrazine triggers converted from SiaTz to activate CyTCO/AIPH for synergistic photothermal/thermodynamic cancer therapy was studied via methyl thiazolyl tetrazolium (MTT) assays. CyTCO/AIPH and NIR laser irradiation were biocompatible, but when the cancer cells were pretreated with Lipo‐SiaTz to express tetrazine triggers, CyTCO/AIPH could lead to a pronounced decrease in their viabilities after 5 min of laser irradiation (Figure S28). Moreover, CyTCO/AIPH combination displayed a stronger therapeutic effect than CyTCO alone (Figures S28 and S29).

For the cancer cells without expression of tetrazine triggers (not pretreated with Lipo‐SiaTz), even the use of CyTCO/AIPH combination followed by laser irradiation failed to induce high cytotoxicity (Figure S28). Notably, a control precursor (Sia) lacking tetrazine groups failed to activate CyTCO/AIPH (Figure S30), which fully demonstrated that only the presence of tetrazine triggers converted from SiaTz precursors could initiate the activation of the therapeutic combination CyTCO/AIPH for effective tumor cell elimination.

To intuitively evaluate the activation effect of SiaTz‐converted tetrazine triggers on CyTCO/AIPH, Calcein AM/Propidium Iodide (PI) costaining experiments were also performed to visualize living (green signal) and dead (red signal) cancer cells in different groups via fluorescence. As shown in Figure 3h, the cancer cells lacking tetrazine triggers (no Lipo‐SiaTz pretreatment) retained strong green fluorescence after CyTCO/AIPH and laser irradiation, indicating minimal cell death. In contrast, tetrazine‐expressing cells (pretreated with Lipo‐SiaTz) showed intense red fluorescence, suggesting that the unnatural tetrazine triggers converted from SiaTz could successfully activate the CyTCO/AIPH combination to induce severe cell damage upon laser irradiation, aligning with MTT assay findings. Additionally, flow cytometry with a commercial fluorescent probe 2′,7′‐dichlorodihydrofluorescein diacetate (DCFH‐DA) was conducted to confirm that SiaTz‐converted tetrazine triggers indirectly induced AIPH decomposition to release free radicals for thermodynamic therapy. As shown in Figure 3i, CyTCO/AIPH alone did not significantly enhance fluorescence, indicating that almost no free radicals were produced. However, in tetrazine‐expressing cells (pretreated with Lipo‐SiaTz), CyTCO/AIPH with NIR laser irradiation led to strong fluorescence, whereas CyTCO alone failed to do so (Figure S29c). These flow cytometric data demonstrated that tetrazine triggers activated CyTCO via bio‐orthogonal reaction, elevating temperature under laser irradiation and subsequently triggering AIPH decomposition for synergistic therapy.

### Application of SiaTz‐Converted Tetrazine Triggers in Activating Theranostic Agents for Diagnostic Imaging and Image‐Guided Therapy of Subcutaneous Tumors in Mice

Inspired by the above results, we employed the SiaTz precursors to construct unnatural tetrazine triggers in the tumors of living mice and investigated their ability to activate the theranostic combination CyTCO/AIPH for diagnosis and localization of the tumors via MSOT imaging and image‐guided synergistic photothermal/thermodynamic therapy. To efficiently deliver the theranostic combination into tumors and use it to verify the activation capability of SiaTz‐converted tetrazine triggers in vivo, CyTCO and AIPH were prepared into pH‐sensitive liposomes (pHLipo‐CyTCO/AIPH) by assembly with a pH‐responsive phospholipid DSPE‐PEOz, which can accelerate the release of payloads in the acidic environments.^[^ ^35^, ^36^ ^]^ pHLipo‐CyTCO/AIPH exhibited a stable 225 nm diameter at pH 7.4 but rapidly disintegrated at pH 6.5, releasing 90% of their payload within 12 h (Figure S31a–c). TEM images confirmed its pH sensitivity, showing intact spherical structures at pH 7.4 and fragmentation at pH 6.5 (Figure S31d). These findings suggest pHLipo‐CyTCO/AIPH is expected to expeditiously release the loaded CyTCO/AIPH in the acidic tumor microenvironment to validate the activation capacity of SiaTz‐converted tetrazine triggers in vivo. The biosafety of pHLipo‐CyTCO/AIPH and Lipo‐SiaTz was also evaluated in mice after intravenous injection. Blood analysis, serum biochemistry, body weight trends, and organ histology showed no obvious abnormalities across groups, suggesting that both formulations had good long‐term biocompatibility (Figure S32).

**Figure 4:**
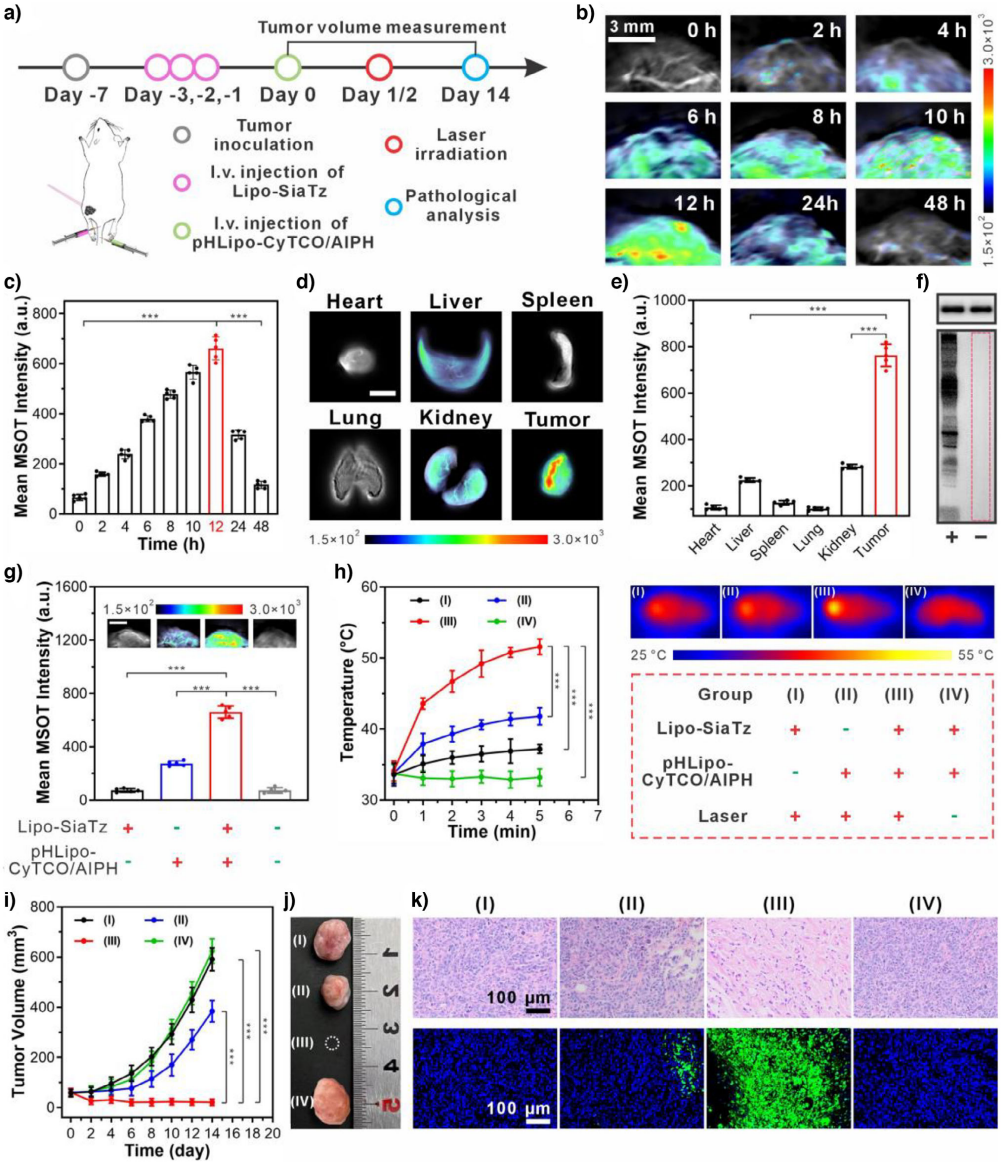
Application of SiaTz‐converted tetrazine triggers in activating PTT/TDT combination therapy of subcutaneous tumors in mice. a) Timeline for liposome administration and synergistic photothermal/thermodynamic therapy in mice with subcutaneous tumor. b) Representative MSOT images and c) mean MSOT intensities of the tumor region of Lipo‐SiaTz‐pretreated mice at different time points after intravenous injection of pHLipo‐CyTCO/AIPH (scale bar: 3 mm) (*n* = 5). d) Representative MSOT images and e) mean optoacoustic intensities of the major organs and tumor harvested from Lipo‐SiaTz‐pretreated mice 12 h after injection of pHLipo‐CyTCO (scale bar: 5 mm) (*n* = 5). f) Western blot analysis showing the expression level of tetrazine triggers in the subcutaneous tumors from mice administered with (+) or without (−) Lipo‐SiaTz. GAPDH (upper panel) was selected as the internal reference. g) Mean MSOT intensities and typical MSOT images (insets) of the tumor areas in different groups of mice. (scale bar: 3 mm) (*n* = 5). h) Temperature rise curves of subcutaneous tumors in different group of mice after NIR laser irradiation for different times and the representative thermal images of the mice in different group after 5 min of laser irradiation (*n* = 5). Grouping details: (I) pretreatment with Lipo‐SiaTz for 3 consecutive days followed by laser irradiation (no pHLipo‐CyTCO/AIPH administration); (II) administration with pHLipo‐CyTCO/AIPH followed by laser irradiation (no Lipo‐SiaTz pretreatment); (III) pretreatment with Lipo‐SiaTz for 3 consecutive days followed by pHLipo‐CyTCO/AIPH administration and laser irradiation; (IV) pretreatment with Lipo‐SiaTz for 3 consecutive days followed by pHLipo‐CyTCO/AIPH administration (no laser irradiation). i) Tumor volume of mice with subcutaneous tumors in different groups after treatment (*n* = 5). j) Typical photographs of excised tumors from mice in each group 14 days after different treatment. k) Representative H&E (upper panel) and TUNEL (lower panel) staining slides of excised tumors from different groups of mice 14 days after treatment. Data were represented as mean values ± standard deviation. Statistical significance was determined by two‐tailed t test. ^***^ *p* < 0.001.

To easily assess the contribution of SiaTz‐converted tetrazine triggers to enhancing the imaging contrast and therapeutic efficacy, we established a subcutaneous 4T1 tumor model in mice. Then these tumor‐bearing mice were randomly divided into several groups and received different administrations (Figure 4a). Through the spectral unmixing function,^[^ ^37^, ^38^ ^]^ MSOT imaging revealed a gradual increase in optoacoustic signal of activated CyTCO at tumors, peaking at 12 h postinjection before declining due to metabolism (Figure 4b,c). Ex vivo MSOT imaging and western blot analysis confirmed specific tetrazine trigger expression in tumors, leading to stronger optoacoustic signals (Figure 4d–g). These results demonstrated that the presence of tetrazine triggers enabled to enhance the signal‐to‐background ratio of MSOT imaging through activating the administered theranostic agents, thus improving the sensitivity in tumor detection and diagnosis.

Encouraged by the imaging results, we next examined the role of SiaTz‐converted tetrazine triggers in activating CyTCO/AIPH for tumor elimination. At 12 h postinjection of pHLipo‐CyTCO/AIPH, tumor sites were irradiated with an NIR laser (1 W cm^−2^) to evaluate in vivo light‐to‐heat conversion efficacy of CyTCO in the presence or absence of tetrazine triggers. In the control groups (I, II, and IV), tumor temperatures remained below 42 °C, insufficient for effective PTT and AIPH pyrolysis (Figure 4h). In contrast, Lipo‐SiaTz‐pretreated mice (Group III) exhibited a rapid temperature rise, reaching 51.6 ± 1.1 °C in 5 min, demonstrating tetrazine‐mediated CyTCO activation for enhanced in vivo photothermal conversion and effective initiation of PTT/TDT treatment.

The therapeutic efficacy of SiaTz‐converted tetrazine triggers in activating CyTCO/AIPH for synergistic PTT/TDT treatment was assessed by recording the tumor volumes within 14 days (Figure 4i,j). Among all the tested groups, the mice expressing tetrazine triggers in tumors (Group III) displayed the most pronounced inhibitory effect after treatment, while tumors in the other control groups (I, II, and IV) presented an uncontrollable and rapid growth trend. Histological examination further confirmed reduced tumor cell density and increased apoptosis in Group III, as evidenced by hematoxylin and eosin (H&E) and terminal deoxynucleotidyl transferase dUTP nick end labeling (TUNEL) staining (Figure 4k). These observations aligned with tumor volume measurements, indicating that tetrazine trigger‐mediated CyTCO/AIPH activation effectively induced tumor cell death under laser irradiation. Moreover, Group III demonstrated the highest survival rate and few recurrences upon posttreatment (Figure S33a,c), highlighting the critical role of SiaTz‐converted tetrazine triggers in activating PTT/TDT for effective tumor elimination and prolonged survival. These investigations illustrate that tetrazine triggers can be constructed in vivo from SiaTz precursors for activation of theranostic agents, enabling diagnostic imaging and image‐guided therapy.

### Application of SiaTz‐Converted Tetrazine Triggers in Activating Theranostic Agents for Diagnostic Imaging and Image‐Guided Therapy of Orthotopic Liver Tumors in Mice

To further validate the construction and application of tetrazine triggers, we employed an orthotopic liver tumor (OLT) mouse model, which better mimics the tumor microenvironment than the subcutaneous model.^[^ ^39^, ^40^ ^]^ The OLT model was created by intrahepatic injection of luciferase‐transfected HCC‐LM3 (HCC‐LM3‐fLuc) cells into the left lobe of mouse livers (Figure S34a) and monitored via bioluminescence imaging.^[^ ^41^ ^]^ As shown in Figure S34b,c, strong bioluminescence indicated tumor formation in OLT mice, while sham‐operated mice showed negligible signals. H&E staining further revealed significant morphological abnormalities in liver sections of OLT mice, confirming the presence of tumors histologically (Figure S34d).

**Figure 5:**
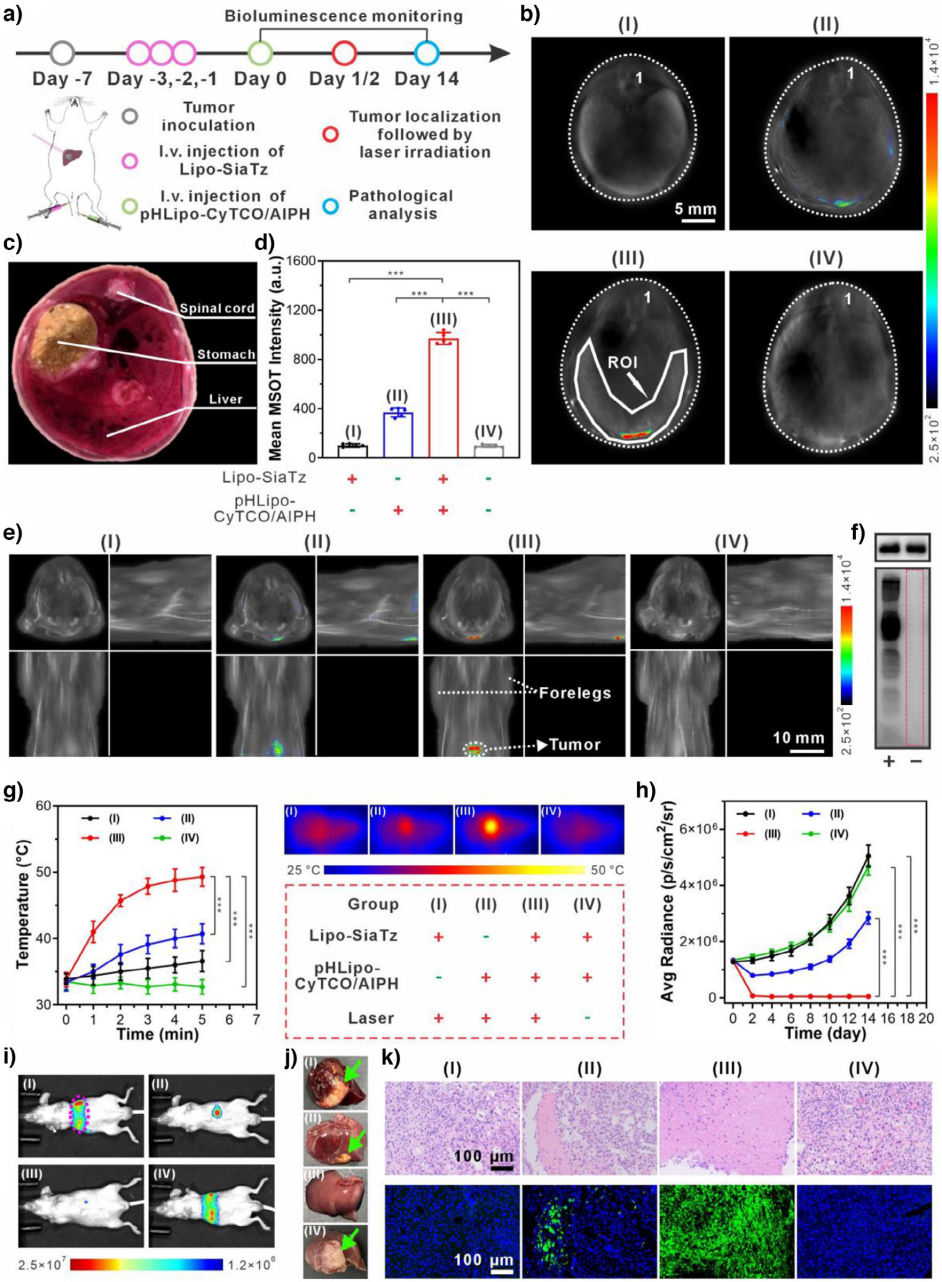
Application of SiaTz‐converted tetrazine triggers in activating the CyTCO/AIPH combination for lesion localization and subsequent PTT/TDT therapy of orthotopic liver tumors in mice. a) Timeline for liposome administration, lesion localization imaging and image‐guided synergistic photothermal/thermodynamic therapy in mice with orthotopic liver tumor. b) Representative cross‐sectional MSOT images of mice in different group. Organ mark: 1) spinal cord. The liver region of mice was delineated by a white line and defined as ROI. c) Cryosection image of a male mouse corresponding to the cross‐section in (b). d) Mean MSOT intensities at ROI of liver regions of mice in different group (*n* = 5). e) Representative orthogonal MIP MSOT images of the mice in different group. f) Western blot analysis showing the expression level of tetrazine triggers in the orthotopic liver tumors from mice administered with (+) or without (−) Lipo‐SiaTz. GAPDH (upper panel) was selected as the internal reference. g) Temperature rise curves of liver tumors in different group of mice after NIR laser irradiation for different times and the representative thermal images of the mice in different group after 5 min of laser irradiation (*n* = 5). Grouping details: (I) pretreatment with Lipo‐SiaTz for 3 consecutive days followed by laser irradiation (no pHLipo‐CyTCO/AIPH administration); (II) administration with pHLipo‐CyTCO/AIPH followed by laser irradiation (no Lipo‐SiaTz pretreatment); (III) pretreatment with Lipo‐SiaTz for 3 consecutive days followed by pHLipo‐CyTCO/AIPH administration and laser irradiation; and (IV) pretreatment with Lipo‐SiaTz for 3 consecutive days followed by pHLipo‐CyTCO/AIPH administration (no laser irradiation). h) Bioluminescence intensity at ROI of mice bearing orthotopic liver tumor after different treatment (*n* = 5). i) Typical bioluminescence images of mice bearing orthotopic liver tumor 14 days after different treatment. The liver region of mice was delimited with a pink dashed circle and defined as ROI. j) Representative photographs of excised liver from different groups of mice 14 days after treatment. The solid tumors in liver were indicated by green arrows. k) Representative H&E (upper panel) and TUNEL (lower panel) staining slides of excised livers from different groups of mice bearing orthotopic liver tumor 14 days after treatment. Data were represented as mean values ± standard deviation. Statistical significance was determined by two‐tailed *t* test. ^***^ *p* < 0.001.

OLT‐bearing mice were randomly divided into four groups and treated according to the timeline in Figure 5a. MSOT imaging was performed 12 h after pHLipo‐CyTCO/AIPH administration. As shown in Figure 5b–d, a strong and concentrated MSOT signal was observed in the liver region of tetrazine trigger‐expressing mice (Group III). In contrast, weak signals were detected in nontetrazine‐expressing mice (Group II), which was attributed to the accumulation of unactivated CyTCO via the EPR effect. Groups I and IV showed almost no signal due to the absence of CyTCO. These results reflected the successful expression of tetrazine triggers in OLTs, enabling high‐contrast tumor detection through CyTCO activation.

MSOT imaging is able to provide orthogonal maximal intensity projection (MIP) images by stacking cross‐sectional scans, allowing precise lesion localization to guide medical interventions.^[^ ^42^, ^43^ ^]^ As indicated from Figure 5e, the orthogonal MSOT MIP image accurately pinpointed the orthotopic liver tumor in Lipo‐SiaTz‐pretreated mice (Group III), which was in good agreement with the bioluminescence imaging results in Figure S34b. Western blot analysis of resected tumors confirmed the expression of tetrazine triggers in Lipo‐SiaTz‐pretreated mice, while no expression was detected in untreated controls (Figure 5f). These findings fully demonstrated that Lipo‐SiaTz administration induces tetrazine trigger expression in orthotopic liver tumors, enhancing tumor imaging contrast by activating CyTCO in situ.

Guided by the acquired MSOT MIP images, the location of the orthotopic liver tumors could be easily determined using the two forelegs as reference points. At around 12 h post pHLipo‐CyTCO/AIPH injection, NIR laser was applied to the area identified as a tumor. After 5 min, liver tumor temperatures remained below 41 °C in Groups I, II, and IV but reached 49.3 ± 1.4 °C in Group III, demonstrating the tetrazine triggers had the potential to activate CyTCO/AIPH for synergistic PTT/TDT treatment. (Figure 5g). Afterward, the therapeutic efficacy of tetrazine trigger‐induced CyTCO/AIPH activation was evaluated by bioluminescence intensity at the liver site. As shown in Figure 5h,i, bioluminescence in control groups (I, IV) steadily increased, spreading across the liver by day 14. Group II showed a transient decline before gradual regrowth, indicating unactivated CyTCO/AIPH failed to eliminate tumors. In contrast, the bioluminescence of Group III quickly dropped to a nearly negligible level after treatment, suggesting effective tumor eradication by tetrazine trigger‐activated CyTCO/AIPH.

After bioluminescence monitoring, some mice were euthanized, and their livers were harvested for photography. As seen from Figure 5j, by day 14 post‐treatment, no tumor mass was found in the livers of Group III, whereas residual tumors remained in Group II. In control groups (I, IV), tumors grew extensively, occupying most of the liver. Additionally, H&E staining and TUNEL assays confirmed extensive tumor necrosis/apoptosis only in Group III (Figure 5k), consistent with bioluminescence imaging results Furthermore, Group III presented the highest survival rate within 45 days after treatments, with rare relapses (Figure S33b,d). These results strongly revealed that tetrazine triggers converted from SiaTz precursors can achieve diagnostic imaging and image‐guided therapy of deep‐seated orthotopic liver tumors through in‐situ bio‐orthogonal activation of the administered theranostic agents.

### Application of SiaTz‐Converted Tetrazine Triggers in Activating Theranostic Agents for Diagnostic Imaging and Image‐Guided Therapy of Lymphatic Metastasis in Mice

**Figure 6:**
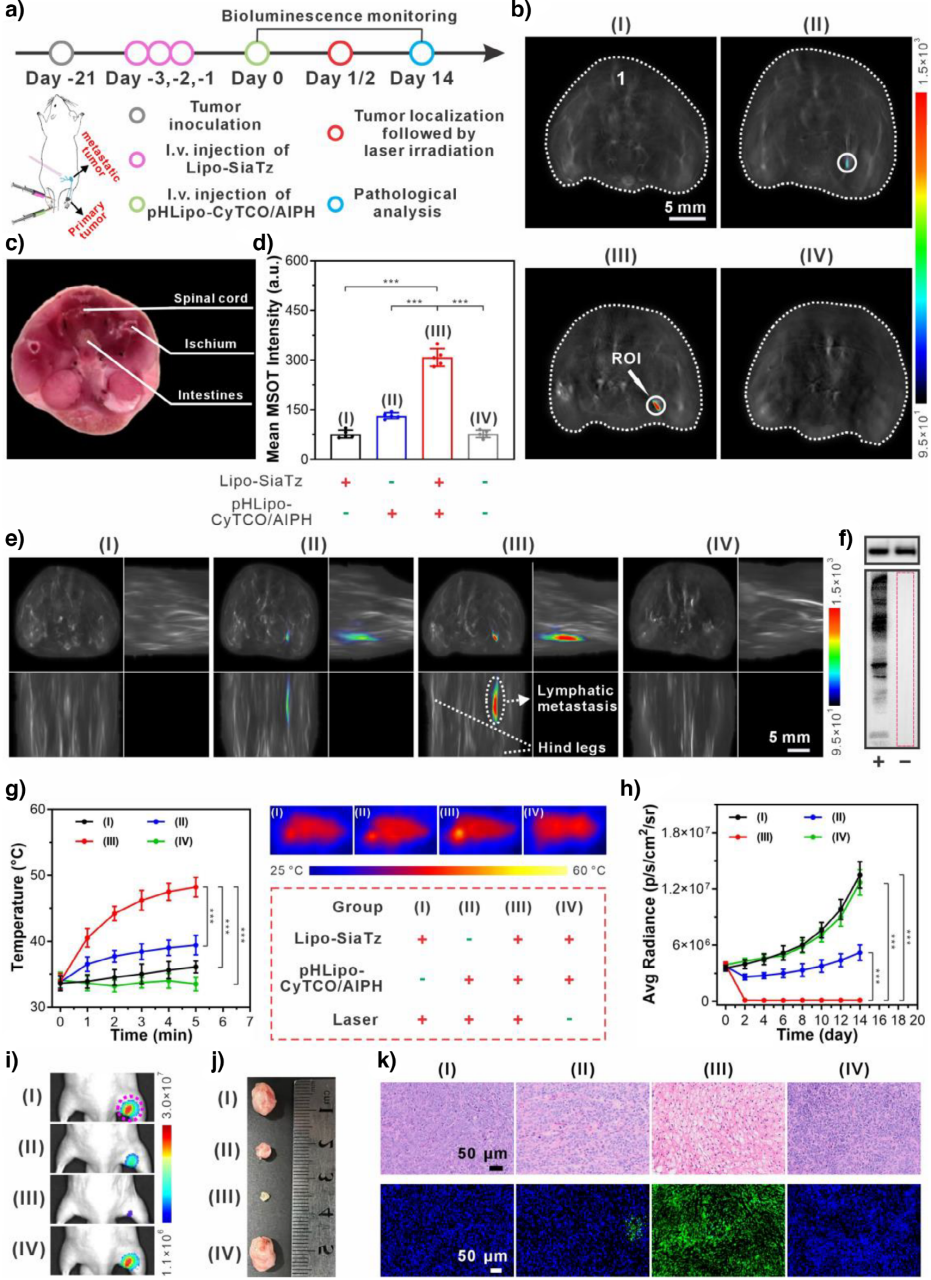
Application of SiaTz‐converted tetrazine triggers in activating the CyTCO/AIPH combination for lesion localization and subsequent PTT/TDT therapy of lymphatic metastasis in mice. a) Timeline for liposome administration, lesion localization imaging, and image‐guided synergistic photothermal/thermodynamic therapy in mice with lymphatic metastasis. b) Representative cross‐sectional MSOT images of mice in different group. Organ mark: 1) spinal cord. The popliteal lymph node region of mice was delimited with a white circle and defined as ROI. c) Cryosection image of a male mouse corresponding to the cross‐section location in (b). d) Mean MSOT intensities at ROI of popliteal fossa positions of mice in different group (*n* = 5). e) Representative orthogonal MIP MSOT images of the mice in different group. f) Western blot analysis showing the expression level of tetrazine triggers in the lymphatic metastatic tumors from mice administered with (+) or without (−) Lipo‐SiaTz. GAPDH (upper panel) was selected as the internal reference. g) Temperature rise curves of lymphatic metastatic tumors in different group of mice after NIR laser irradiation for different times and the representative thermal images of the mice in different group after 5 min of laser irradiation (*n* = 5). Grouping details: (I) pretreatment with Lipo‐SiaTz for 3 consecutive days followed by laser irradiation (no pHLipo‐CyTCO/AIPH administration); (II) administration with pHLipo‐CyTCO/AIPH followed by laser irradiation (no Lipo‐SiaTz pretreatment); (III) pretreatment with Lipo‐SiaTz for 3 consecutive days followed by pHLipo‐CyTCO/AIPH administration and laser irradiation; and (IV) pretreatment with Lipo‐SiaTz for 3 consecutive days followed by pHLipo‐CyTCO/AIPH administration (no laser irradiation). h) Bioluminescence intensity at ROI of mice with lymphatic metastasis after different treatment (*n* = 5). i) Typical bioluminescence images of mice bearing lymphatic metastatic tumor 14 days after different treatment. The popliteal lymph node region of mice was delimited with a pink dashed circle and defined as ROI. j) Representative photographs of excised lymphatic metastatic tumors from different groups of mice 14 days after treatment. k) Representative H&E (upper panel) and TUNEL (lower panel) staining slides of excised lymphatic metastatic tumors from different groups of mice 14 days after treatment. Data were represented as mean values ± standard deviation. Statistical significance was determined by two‐tailed *t* test. ^***^ *p* < 0.001.

Given that theranostics has also been attempted to expand its application in the diagnosis and treatment of metastatic tumors in recent decades, we next established a mouse model of lymphatic metastasis to explore the potential of tetrazine triggers converted from the SiaTz precursors in theranostics of metastatic tumors. As shown in Figure S35, on day 21 postinoculation of 4T1‐fLuc cells into the footpad, strong bioluminescence signals appeared at both the footpad (primary tumor) and adjacent popliteal lymph node (metastatic site), indicating tumor metastasis. Photographs and H&E staining of lymph nodes further validated the successful model establishment. The metastatic tumor‐bearing mice were divided into four groups and treated per the timeline in Figure 6a. Following pHLipo‐CyTCO/AIPH injection, Group III (Lipo‐SiaTz pretreatment) exhibited stronger MSOT signals at the popliteal lymph node than Group II (no Lipo‐SiaTz pretreatment) (Figure 6b,c). The control groups (I and IV) lacking CyTCO administration showed no exogenous MSOT signals (Figure 6b,d). More importantly, metastatic tumors of Group III were accurately mapped with a higher signal‐to‐noise ratio in the MSOT MIP image (Figure 6e). Western blot analysis (Figure 6f) confirmed tetrazine trigger expression in metastatic tumors, further demonstrating their role in facilitating high signal‐to‐noise imaging diagnosis and localization through bio‐orthogonal activation of theranostic agents.

Determining the temperature changes within tumors could help assess the capacity of SiaTz‐converted tetrazine triggers to activate administered agents for photothermal and thermodynamic therapy. As displayed in Figure 6g, pHLipo‐CyTCO/AIPH administration followed by laser irradiation expeditiously raised the intratumoral temperature to 48.2 ± 1.5 °C in Group III, whereas it only reached 39.4 ± 1.5 °C in Group II. The control groups (I and IV) showed even lower intratumoral temperatures, insufficient for effective PTT/TDT. These findings highlight the potential of SiaTz‐converted tetrazine triggers to activate CyTCO for thermal tumor ablation and AIPH cleavage to generate cytotoxic free radicals within the metastases for cascade thermodynamic therapy.

To prove the above inference, bioluminescence intensity at the lymphatic metastasis sites was measured (Figure 6h,i). Although Group II showed a slower increase in bioluminescence compared to Groups I and IV, it never ceased. On the contrary, Group III displayed a significant reduction in bioluminescence, with the signal nearly disappearing 14 days post‐treatment. As shown in Figure 6j, metastatic tumors in Group III were notably shrunken and hardened. Furthermore, the H&E and TUNEL staining of resected tumors in Group III revealed extensive apoptosis and necrosis (Figure 6k), supporting the bioluminescence findings. Together, these results strongly demonstrated that SiaTz‐converted tetrazine triggers could activate the administered formulations and enhance their therapeutic efficacy against metastatic tumors.

## Conclusion

The construction of unnatural triggers through metabolic glycoengineering serves as a promising approach to remodeling cell membranes with chemical groups, which makes it possible to achieve highly specific targeted imaging and therapy of tumors via bio‐orthogonal reaction, especially for the tumors lacking endogenous targets. In summary, we have rationally designed and synthesized a tetrazine precursor SiaTz based on the non*O*‐acetylated sialic acid skeleton and confirmed its excellent stability and satisfactory reaction kinetics under physiological conditions. Compared with the control mannose‐based precursor ManTz with the same bulky tetrazine group, SiaTz allows for more efficient generation of tetrazine triggers as it can bypass the size‐limiting steps in metabolic glycoengineering. More importantly, after incubation with the liposomal SiaTz precursors, cancerous cells can express significantly more unnatural tetrazine triggers on their surfaces than noncancerous ones due to the abnormal activity of enzymes related to metabolic glycoengineering, thus enabling the generated tetrazine triggers to be used as targets for selective imaging and therapy.

Unlike the commonly‐used azide targets which can only covalently bind to molecules with complementary groups, the tetrazine triggers are also capable of initiating the in‐situ release of active imaging/therapeutic components from the complementary group TCO‐caged agents. To validate the activation function, we have also developed a proof‐of‐concept theranostic agent CyTCO and then used it together with a thermal‐sensitive drug AIPH to form an activatable theranostic combination CyTCO/AIPH. Upon responding to tetrazine group via bio‐orthogonal reaction, CyTCO can be activated to release the active optoacoustic/photothermal compound CyNH. On one hand, the enhanced optoacoustic signal from the tetrazine trigger‐activated CyTCO facilitates high‐contrast tumor diagnostic imaging and enables precise localization of tumor focus with the help of z‐stack MIP images generated by the MSOT system to guide subsequent treatments. On the other hand, the tetrazine trigger‐activated CyTCO with an intensified photothermal conversion performance is able to induce a rapid increase in temperature and further initiates AIPH pyrolysis to produce cytotoxic free radicals for synergistic PTT/TDT tumor therapy. The vital role of the unnatural tetrazine triggers in activating MSOT imaging and subsequent imaged‐guided combination therapy has been well revealed in the mouse models of subcutaneous tumors, orthotopic liver tumors, and lymphatic metastasis. For the deeper tumors, activatable theranostic combination with strong absorption at NIR‐II or even longer regions can be consciously designed and developed to fully exert their diagnostic and therapeutic functions upon tetrazine trigger activation and longer‐wavelength laser irradiation with deeper tissue penetration. Thus, this study herein fully demonstrates the powerful functions of unnatural tetrazine triggers converted from tetrazine‐attached non*O*‐acetylated sialic acid precursors in cancer theranostics, and offers helpful insights into the development of other theranostic systems that can be activated by tetrazine triggers, thereby providing an opportunity for realizing personalized medicine in future clinical practice.

## Conflict of Interests

The authors declare no conflict of interest.

## Supporting information



Supporting Information

## Data Availability

The data that support the findings of this study are available from the corresponding author upon reasonable request.
