# Supplementary material for: Unnatural Triggers Converted From Tetrazine‐Attached Sialic Acid for Activation of Optoacoustic Imaging‐Guided Cancer Theranostics
Source: Angew Chem Int Ed Engl. 2025 May 2;64(26):e202503850. doi: 10.1002/anie.202503850 (PMC12184287; doi:10.1002/anie.202503850)
Supplement: Supplementary file 1 — Supporting Information [file ANIE-64-e202503850-s001.docx]

**Supporting Information**

**Experimental Section**

**Chemicals.** Acetic acid, cholesterol, *trans*-cyclooctene (TCO)-amine hydrochloride salt, DSPE-PEG_2000_, HRP-streptavidin, lecithin, D-mannosamine hydrochloride, N-methylpyridin-4-amine, 2-methylquinoline, phosphorus oxychloride, sodium acetate, sodium azide, 4-(tert-butyl)cyclohexan-1-one, p-toluenesulfonyl chloride, triethylamine, and triphenylphosphine as well as anhydrous solvents such as acetone, acetonitrile (MeCN), chloroform (CHCl_3_), N,N-dimethylformamide (DMF), ethanol, pyridine, and tetrahydrofuran (THF) were purchased from Sigma-Aldrich. 2,2'-Azobis[2-(2-imidazolin-2-yl)propane] dihydrochloride (AIPH), N,N-diisopropylethylamine, and 4-dimethylaminopyridine were procured from Tokyo Chemical Industry Co. Ltd. N-acetylneuraminic acid methyl ester, (E)-cyclooct-2-en-1-yl(4-nitrophenyl) carbonate, and 1-(iodomethoxy)-2-(2-methoxyethoxy)ethane were obtained from BLD Pharmatech Ltd. and used without further purification. AZDye 488 TCO and methyltetrazine-NHS ester were purchased from Vector Laboratories, Inc. DSPE-PEOz_2000_ was bought from Xi’an ruixi Biological Technology Co., Ltd. TCO-PEG_4_-biotin was acquired from Broadpharm. The solvents for purification including acetone, dichloromethane (DCM) and methanol (MeOH) were of analytical grade. Triple-distilled water was used throughout the experiments.

**Characterizations.** ^1^H and ^13^C NMR spectra were measured with a Bruker BBFO 400 Spectrometer. The liquid chromatography-mass spectra (LC-MS) were recorded on a ThermoFinnigan LCQ quadrupole ion trap mass spectrometer. The high-resolution mass spectra (HR-MS) were obtained from TripleTOF 5600+ system. The absorption spectra were collected on UV-3600 Shimadzu UV-Vis-NIR Spectrophotometer. Transmission electron microscopy (TEM) images were collected on JEOL JEM-1400. The size distributions of liposomes were determined through dynamic light scattering (DLS) by using Malvern Instruments Zetasizer Nano-S at 25 ℃. Photothermal profiles were recorded by irradiating the samples with an 808 nm NIR laser and the images were captured by a thermal imager. HPLC data were acquired from Agilent 1260 Infinity liquid chromatograph (with DAD). ESR spectra were recorded using a Bruker ER200-SRC-10/12 ESR spectrometer. MTT assay was measured with a Varioskan™ LUX multimode microplate reader. Flow cytometry was taken on a BD FortessaX20 5-laser cytometer. Confocal laser scanning microscopy (CLSM) images were acquired by ZEN 2 (blue edition) software of Carl Zeiss LSM 800. Optoacoustic imaging was performed on an inVision128 multispectral optoacoustic tomographic (MSOT) imaging system (iThera Medical GmbH). Bioluminescence images were acquired from an AMI small animal fluorescence imaging system (Spectral Instruments Imaging Co.).

**Synthesis of compound 1.** In a single-neck round-bottom flask, the starting chemical N-acetylneuraminic acid methyl ester (3.23 g, 10 mmol) was dissolved in anhydrous pyridine (40 mL) and then cooled to 0 °C. Next, p-toluenesulfonyl chloride (2.0 g, 10.5 mmol) was added into the solution. After the mixture was stirred at room temperature overnight, the solvent was removed and then the residue was purified by column chromatography using the eluent dichloromethane/methanol (10:1 v/v) to give the product Compound 1 as a white solid (3.39 g, yield: 71.3%). ^1^H NMR (400 MHz, DMSO-*d*_6_): δ 8.11 (d, *J* = 8.3 Hz, 1H), 7.75 (d, *J* = 8.2 Hz, 2H), 7.47 (d, *J* = 8.1 Hz, 2H), 6.36 (d, *J* = 1.7 Hz, 1H), 4.90-4.67 (m, 3H), 4.20 (d, *J* = 9.6 Hz, 1H), 3.94-3.79 (m, 2H), 3.73-3.64 (m, 5H), 3.45 (q, *J* = 10.0 Hz, 1H), 3.20 (d, *J* = 8.8 Hz, 1H), 2.42 (s, 3H), 2.02 (dd, *J* = 12.8, 4.9 Hz, 1H), 1.89 (s, 3H), 1.70 (t, *J* = 12.8, Hz, 1H). LC-MS (m/z): 478.17 [M+H]^+^.

**Synthesis of compound 2.** Compound 1 (954 mg, 2 mmol) and sodium azide (650 mg, 10 mmol) were dissolved in water (5 mL) and then diluted with acetone (15 mL) in a sealed tube. After being refluxed overnight, the mixture was evaporated in vacuo to remove the solvents. The resulting residue was purified by flash chromatography using dichloromethane/methanol (10:1 v/v) as eluent to afford the product Compound 2 as a pale yellow solid (530 mg, yield: 80%). ^1^H NMR (400 MHz, D_2_O): δ 3.94 (d, *J* = 10.1 Hz, 2H), 3.85-3.76 (m, 2H), 3.53-3.44 (m, 2H), 3.36 (dd, *J* = 13.1, 6.0 Hz, 1H), 2.20 (dd, *J* = 13.1, 4.9 Hz, 1H), 1.95 (s, 3H), 1.81-1.73 (m, 1H). LC-MS (m/z): 334.27 [M]^+^.

**Synthesis of compound 3.** Compound 2 (334 mg, 1 mmol) was dissolved in water (4 mL) and then a solution of triphenylphosphine (787 mg, 3 mmol) in tetrahydrofuran (6 mL) was added. The mixture was stirred at room temperature overnight. Next, 2 mL of acetic acid as well as 20 mL of water was added, and the suspension was stirred for additional 2 hours. After that, the suspension was washed three times with dichloromethane and the aqueous phase was lyophilized. The resulting crude product was further purified in a silica-gel column using dichloromethane/methanol (3:1 v/v) as eluent to give the product Compound 3 as a pale yellow solid (176 mg, 57 %). ^1^H NMR (400 MHz, CD_3_OD): δ 3.96 (m, 5H), 3.50 (d, *J* = 8.1 Hz, 1H), 3.01-2.94 (m, 1H), 2.23 (d, *J* = 12.5 Hz, 1H), 2.02 (s, 3H), 1.82 (t, *J* = 13.2 Hz, 1H). LC-MS (m/z): 309.14 [M+H]^+^.

**Synthesis of SiaTz.** Compound 4 (77 mg, 0.25 mmol) and commercially available methyltetrazine-NHS ester (82 mg, 0.25 mmol) were dissolved with anhydrous dimethylformamide (3 mL) in a single-neck round-bottom flask. After triethylamine (100 μL, 0.75 mmol) was added into the medium, the mixture was stirred at room temperature for 24 h. The solvent was evaporated in vacuo and the resulting residue was purified in a silica-gel column using dichloromethane/methanol (5:1 v/v) as eluent to obtain the target product SiaTz as a pink power (141 mg, yield: 92%). ^1^H NMR (400 MHz, CD_3_OD): δ 8.49 (d, *J* = 8.3 Hz, 2H), 7.56 (d, *J* = 8.3 Hz, 2H), 4.08-3.97 (m, 2H), 3.92-3.73 (m, 3H), 3.72-3.61 (m, 3H), 3.39 (d, *J* = 8.8 Hz, 1H), 3.03 (s, 3H), 2.22 (dd, *J* = 12.8, 4.7 Hz, 1H), 1.99 (s, 3H), 1.93-1.77 (m, 1H). ^13^C NMR (100 MHz, CD_3_OD): δ 175.03, 174.11, 171.75, 168.73, 165.24, 141.9, 132.14, 131.16, 128.97, 96.68, 72.03, 71.81, 70.74, 67.85, 54.34, 44.71, 43.61, 40.70, 22.70, 21.03. LC-MS (m/z): 521.27 [M+H]^+^. HR-MS (m/z): found 521.1990 [M+H]^+^. Solubility in water: ≈ 40 mg/mL.

**Synthesis of ManTz.** D-mannosamine hydrochloride (54 mg, 0.25 mmol) and methyltetrazine-NHS ester (82 mg, 0.25 mmol) were dissolved with anhydrous dimethylformamide (3 mL) in a single-neck round-bottom flask. After triethylamine (100 μL, 0.75 mmol) was added into the medium, the mixture was stirred at room temperature for 24 h. The solvent was evaporated in vacuo and the resulting residue was purified in a silica-gel column using dichloromethane/methanol (8:1 v/v) as eluent to obtain the target product ManTz as a pink power (86 mg, yield: 88%).^1^H NMR (400 MHz, CD_3_OD): δ 8.46 (d, *J* = 6.6 Hz, 2H), 7.57 (d, *J* = 7.6 Hz, 2H), 5.04 (s, 1H), 4.32 (s, 1H), 4.05(d, *J* = 11.6 Hz, 1H), 3.81 (m, 3H), 3.73 (s, 2H), 3.64 (m, 1H), 3.02 (s, 1H). ^13^C NMR (100 MHz, CD_3_OD): δ 173.84, 168.65, 165.21, 142.07, 131.93, 131.20, 128.83, 94.86, 73.46, 70.54, 68.57, 62.32, 55.36, 43.40, 21.04. LC-MS (m/z): 392.07 [M+H]^+^. HR-MS (m/z): found 392.1568 [M+H]^+^. Solubility in water: ≈ 22.5 mg/mL.

**Synthesis of compound 4.** Freshly distilled dimethylformamide (20 mL, 273 mmol) was added into a 250 mL two-neck round flask under an argon atmosphere. Afterwards, phosphorus oxychloride (20 mL, 131 mmol) dissolved in dichloromethane (20 mL) was added dropwise within 20 min at 0 °C. After being stirred at room temperature for 30 min, a solution of 4-(tert-butyl)cyclohexan-1-one (7.7 g, 50 mmol) in dichloromethane (20 mL) was slowly added into the mixture via a syringe. The solution was refluxed with vigorously stirring for 1.5 h at 80 °C. After that, the resulting mixture was cooled down, poured into ice-cold water and allowed to stand in a refrigerator overnight. The suspension was filtered, and the precipitate was collected and dried in vacuum as yellow solid without further purification (7.8 g, yield: 68%).

**Synthesis of compound 5.** 2-Methylquinoline (2.86 g, 20 mmol) and 1-(iodomethoxy)-2-(2-methoxyethoxy)ethane (5.48 g, 20 mmol) were dissolved in anhydrous acetonitrile (10 mL) in a sealed tube. After being refluxed at 90 °C overnight, the solvent was evaporated in vacuum and the crude product was purified in a silica-gel column using dichloromethane/methanol (10:1 v/v) as eluent. The product was collected and dried in vacuum as a reddish-brown oil (7.67 g, yield: 92%). ^1^H NMR (400 MHz, CDCl_3_): δ 9.00 (d, *J* = 8.5 Hz, 1H), 8.66 (d, *J* = 9.1 Hz, 1H), 8.28 (d, *J* = 8.1 Hz, 1H), 8.13 (t, *J* = 8.6 Hz, 1H), 8.01 (d, *J* = 8.5 Hz, 1H), 7.85 (t, *J* = 7.6 Hz, 1H), 5.45 (s, 2H), 4.17 (s, 2H), 3.48(m, 2H), 3.41 (m, 6H), 3.29 (d, *J* = 5.4 Hz, 6H). LC-MS (m/z): 290.20 [M]^+^.

**Synthesis of compound 6.** Compound 4 (1.14 g, 5 mmol) and Compound 5 (4.17 g, 10 mmol) were dissolved in ethanol (20 mL) followed by addition of sodium acetate (0.82 g, 10 mmol). The mixture was refluxed at 90 °C for 2 h under an argon atmosphere. Afterwards, the solvent was removed by vacuum-rotary evaporation procedure, and the resulting mixture was purified by silica gel chromatography with dichloromethane/methanol (50:1) as eluent to obtain Compound 6 as a dark green solid (4.0 g, yield: 89%). ^1^H NMR (400 MHz, CDCl_3_): δ 7.99 (d, *J* = 10.7 Hz, 2H), 7.87 (d, *J* = 9.4 Hz, 2H), 7.77 (d, *J* = 8.7 Hz, 2H), 7.69-7.56 (m, 6H), 7.29 (d, *J* = 7.4 Hz, 2H), 6.33 (s, 2H), 4.71 (s, 4H), 4.05 (s, 4H), 3.67-3.63 (m, 4H), 3.59-3.55 (m, 4H), 3.52-3.48 (m, 4H), 3.43-3.40 (m, 4H), 3.28 (s, 6H), 2.81 (s, 2H), 2.16 (s, 2H), 1.55 (s, 1H), 1.07 (s, 9H). LC-MS (m/z): 771.63 [M]^+^

**Synthesis of compound 7.** Compound 6 (898 mg, 1 mmol) and N-methylpyridin-4-amine (540 mg, 5 mmol) were dissolved in anhydrous acetonitrile (10 mL). The mixture was stirred at 50 °C overnight under an argon atmosphere. Afterwards, the solvent was removed by vacuum-rotary evaporation procedure, and the resulting mixture was purified by aluminum oxide chromatography with dichloromethane/methanol (30:1) as eluent to obtain Compound 7 as a dark green solid (610 mg, yield: 61%). ^1^H NMR (400 MHz, CDCl_3_): δ 10.48 (d, *J* = 15.3 Hz, 1H), 8.15 (s, 1H), 7.86 (d, *J* = 8.6 Hz, 3H), 7.78 (d, *J* = 8.7 Hz, 2H), 7.68 (t, *J* = 7.8 Hz, 4H), 7.47 (t, *J* = 6.5 Hz,1H), 7.40 (t, *J* = 7.4 Hz, 2H), 7.32 (d, *J* = 9.3 Hz, 2H), 7.15 (d, *J* = 23.6 Hz, 1H), 6.60 (d, *J* = 13.2 Hz, 2H), 6.45 (d, *J* = 13.5 Hz, 2H), 4.73 (s, 4H), 4.05 (s, 4H), 3.68-3.63 (m, 4H), 3.60-3.55 (m, 4H), 3.53-3.49 (m, 4H), 3.45-3.41 (m, 4H), 3.29 (s, 6H), 3.13 (s, 3H), 2.91-2.82 (m, 2H), 2.31-2.19 (m, 2H), 1.68 (dt, *J* = 25.5, 12.3 Hz, 1H), 1.08 (s, 9H). LC-MS (m/z): 422.58 [M/2]^+^.

**Synthesis of CyTCO.** Compound 7 (200 mg, 0.2 mmol), (E)-cyclooct-2-en-1-yl (4-nitrophenyl) carbonate (291 mg, 1 mmol), N, N-diisopropylethylamine (258 mg, 2 mmol) and 4-dimethylaminopyridine (24 mg, 0.2 mmol) were dissolved in anhydrous dichloromethane (10 mL). The mixture was stirred at room temperature under an argon atmosphere for 48 h. Afterwards, the solvent was removed by vacuum-rotary evaporation procedure, and the resulting mixture was purified in a silica-gel column using dichloromethane/methanol (15:1 v/v) as eluent as eluent to obtain CyTCO as a dark green solid (86 mg, yield: 37%). ^1^H NMR (400 MHz, CDCl_3_): δ 8.60 (d, *J* = 62.0 Hz, 4H), 7.86-7.59 (m, 8H), 7.44 (d, *J* = 38.2 Hz, 4H), 6.49 (s, 4H), 5.88 (s, 1H), 5.58 (d, *J* = 15.7 Hz, 2H), 4.68 (s, 4H), 4.04 (s, 4H), 3.71-3.64 (m, 4H), 3.63-3.58 (m, 4H), 3.56-3.52 (m, 4H), 3.47-3.43 (m, 4H), 3.32 (s, 6H), 2.92 (d, *J* = 16.2 Hz, 2H), 2.51 (d, *J* = 10.1 Hz, 1H), 2.35-2.19 (m, 3H), 2.09-1.70 (m, 8H), 1.58-1.45 (m, 2H), 1.10 (s, 9H), 0.89-0.79 (m, 2H). ^13^C NMR (100 MHz, CDCl_3_): δ 157.25, 152.95, 152.18, 146.88, 146.15, 139.96, 137.97, 136.00, 133.17, 132.94, 129.60, 129.53, 126.48, 126.04, 125.72, 121.28, 117.53,116.28,107.77, 77.86, 77.36, 71.92, 71.16, 70.78, 70.65, 68.35, 59.11, 49.39, 42.22, 40.63, 36.05, 32.62, 29.82, 29.08, 27.52, 27.09, 24.37. LC-MS (m/z): 498.55 [M/2]^+^. HR-MS (m/z): found 498.2977 [M/2]^+^. Solubility in water: ≈ 1.2 mg/mL.

**Preparation of Lipo-SiaTz, Lipo-ManTz and pHLipo-CyTCO/AIPH**. These liposomes were prepared by thin-film hydration. In brief, lecithin, cholesterol, and DSPE-PEG_2000_ were dissolved in chloroform at a molar ratio of 2:1:0.4 in a round-bottom flask. After the chloroform was allowed to evaporate under low pressure, a transparent film was formed at the flask bottom. Then the film was hydrated with saline (3 mL) containing SiaTz (22.2 mg) or ManTz (11.7 mg) and incubated at room temperature for 60 min. As for the pH-sensitive liposomes (pHLipo-CyTCO/AIPH), CyTCO (9 mg) was dissolved in chloroform together with lecithin, cholesterol, and DSPE-PEOz_2000_ (pH-responsive lipid), and then the solvent was evaporated to form a film. Next, the film was hydrated with saline (3 mL) containing AIPH (1.2 mg) and incubated at room temperature for 60 min. After a short while of mild sonication, the resulting emulsion was extruded repeatedly through an Avanti extruder with different pore size filters to obtain nanoparticles with a diameter of approximately 200 nm, which was used for in vitro and in vivo experiments. The loading efficiency (*LE*) and loading capacity (*LC*) were calculated by following equations, and the results were displayed in Table S1:

(1) *LE* (%) = $\frac{mass of loaded component}{mass of added conponent}$ ×100%

(2) *LC* (%) = $\frac{mass of loaded component}{mass of formulation}$ ×100%

**Kinetics determination and stability studies.** The second order rate constant was measured under pseudo-first order conditions using a fixed amount of SiaTz and an excess of a commercially available TCO compound and by monitoring the disappearance of the characteristic tetrazine absorption at 520 nm, similar to previous reports^[1-2]^. Data was collected immediately after rapid mixing of Tz and TCO compound in citrate-phosphate buffered saline (CPBS, 10 mM, pH = 6.5) at 25 °C. The final concentration of SiaTz was 10 µM, and the final concentration of TCO-amine HCl salt was 100, 200, 300, 400 or 500 μM. The observed rate (*k_obs_*) was determined by nonlinear regression analysis of the data points. The *k_obs_* values were then plotted against the concentration of TCO to yield the second order rate constants (*k_2_*) from the slope of the line and the standard error of the slope.

As for the stability of SiaTz, changes in its characteristic absorbance under different solution environments were monitored at different times.

**Cell culture.** Human embryonic lung fibroblast (IMR-90), human mammary epithelial cells (MCF-10A), human lung cancer cells (A549) and mouse mammary adenocarcinoma cells (4T1) were obtained from American Type Culture Collection (ATCC). Human normal liver cells (L-02) were purchased from Ubigene Biosciences Co., Ltd. High metastasis human hepatoma cells (HCC-LM3) were obtained from iCell Bioscience Inc. MCF-10A was incubated in mammary epithelial cell growth basal medium (MEBM) supplemented with MEGM SingleQuot Kit at 37 °C under 5% of CO_2_, IMR-90 was incubated in Eagle's minimum essential medium (EMEM) supplemented with 10% FBS and 1% penicillin and streptomycin at 37 °C under 5% of CO_2_, 4T1 and L-02 were incubated in RPMI-1640 medium supplemented with 10% FBS and 1% penicillin and streptomycin, while A549 and HCC-LM3 were cultured in Dulbecco's modified eagle medium (DMEM) supplemented with 10% FBS and 1% penicillin and streptomycin at 37 °C under 5% of CO_2_. When the cell density reached 70-80% of confluence, subculturing was considered complete. The medium was changed approximately every 1-2 days.

**Cell viabilities.** The relative viabilities of IMR-90, MCF-10A, L-02, 4T1, A549 and HCC-LM3 cells which were exposed to Lipo-SiaTz or Lipo-ManTz were assessed by MTT assay for cytotoxicity studies. These cell lines were seeded in 96-well plates with an initial seeding density of 5 × 10^4^ per milliliter and cultured in the medium. After a 24-h period of incubation at 37 °C under 5% of CO_2_, the cells were washed with pre-warmed PBS solution, and then PBS was substituted with fresh culture medium containing Lipo-SiaTz or Lipo-ManTz (Tz concentration: 0, 25, 50, 100, 200, or 500 µM), after that the cells were subject to incubation for 24 h. Afterwards, the wells were washed with PBS and incubated for another 4 h with medium containing 0.5 mg mL^-1^ MTT. Upon discarding the culture medium, DMSO (150 μL) was added to dissolve the precipitates, and the absorbance was later measured with a Varioskan™ LUX multimode microplate reader at 570 nm. Finally, the statistical mean and standard deviation of absorbance at 570 nm were employed to estimate the relative cell viability. In the assays, for each concentration, three independent experiments were performed.

**Detection of generation of tetrazine groups on cell surface.** The generation of tetrazine groups on cell surface was verified by confocal laser scanning microscopy (CLSM) imaging. The 4T1, A549 and HCC-LM3 cells were seeded with a density of 5 × 10^4^ per milliliter in a confocal dish and allowed to adhere. After 1 day, the original cell media were replaced with fresh ones containing Lipo-SiaTz or Lipo-ManTz (equivalent Tz concentration: 50 μM). After incubation for 48 hours at 37 °C under 5% CO_2_, these cells were sequentially labelled with AZDye 488 TCO (10 μM) and Hoechst 33342 (10 µg mL^-1^) for 10 min. For the control group, the cells were pretreated without any Tz-containing reagent.

The difference in the ability of cancer cells and normal cells to produce Tz triggers was studied by flow cytometry. IMR-90, MCF-10A, L-02, 4T1, A549 and HCC-LM3 cells were seeded onto culture dish at 2 × 10^5^ per milliliter and allowed to culture for 24 h before treatments. Then the original cell media were replaced with fresh ones containing Lipo-SiaTz (equivalent Tz concentration: 50 μM). After incubation for 48 hours at 37 °C under 5% CO_2_, these cells were labelled with AZDye 488 TCO (10 μM) for 10 min. For control groups, the cells were directly labelled with AZDye 488 TCO without Lipo-SiaTz pretreatment. Afterwards, the cells were washed, trypsinized, centrifuged and then resuspended in PBS for flow cytometry. On a BD FortessaX20 5-laser cytometer, the cells were first identified through FSC-A vs. SSC-A to remove debris and free nanoparticles, and then single cells were further identified through FSC-A vs. FSC-H. Finally, the fluorescence intensity of 10,000 cells from each well were recorded and analyzed.

**Stability of tetrazine groups on cell surface.** The stability of tetrazine groups on cell surface was evaluated by measuring the changes in cell optical density at 520 nm (OD_520_, corresponding to the maximum characteristic absorption peak of tetrazine groups). HCC-LM3 cells were first seeded into a petri dish at 5 × 10^4^ cells per milliliter and allowed to adhere after 24 h of culture. Next, the original cell media were replaced with fresh ones containing Lipo-SiaTz (equivalent Tz concentration: 50 μM) to allow the cells to take up these precursors and express Tz triggers. Two days later, after removing the free SiaTz precursors from the culture media, these cells were trypsinized and reseeded onto 96-well plates. The cells with or without Lipo-SiaTz pretreatment were then incubated for different times (0, 12, 24, 36 and 48 h). Finally, the cells in each well were washed, and the OD_520_ value was measured with a microplate reader. For each incubation time, optical density measurement was performed on five independent cell wells.

**Responses of CyTCO to SiaTz.** CyTCO was dissolved in DMSO and then diluting it with CPBS under stirring for optical spectra measurement (final concentration: 10 μM). The absorbance, optoacoustic, photothermal heating trend changes of CyTCO upon the addition of varied amounts of SiaTz in CPBS (10 mM, pH = 6.5) containing 5% (v/v) DMSO were recorded after 5 min of mixing. For selectivity experiments, SiaTz was added into the CyTCO solution together with other biologically relevant substances. The optoacoustic signal changes were measured by filling the test solutions in commercial Wilmad NMR tubes and then acquiring the data on a multispectral optoacoustic tomographic imaging system (inVision 128, iThera Medical GmbH). The absorption spectral variation was measured in a quartz cell (light path, 10 mm) on a UV-3600 Shimadzu UV-Vis-NIR Spectrophotometer. The photothermal heating trend was attained by monitoring the changes in temperature of the solutions of SiaTz alone, CyTCO alone or the mixture of CyTCO and SiaTz after 5 mins of reaction under irradiation with an 808 nm laser (1 W cm^−2^). The temperature of the samples was recorded by an electronic thermometer.

**In vitro apoptosis.** 4T1, A549, and HCC-LM3 cells (5 × 10^3^ cells per well) were seeded in 96-well plates for 12 h, and then the cells were first pretreated with a fresh medium containing Lipo-SiaTz (equivalent Tz concentration: 50 μM) for 2 days. After being washed with PBS, the cells were incubated with CyTCO (10 μM) and AIPH (50 μM) for 6 hours. Next, the cells were irradiated by an 808 nm laser (power density = 1 W cm^-2^) for 5 min and then the irradiated cells were cultured for another 12 h at 37 °C under 5% CO_2_. For the control groups, the cells were treated according to the corresponding formulations. Finally, the cell viability of each group was determined using MTT assays.

Additionally, Calcein-AM/PI staining kit was used to visualize live and dead cells of each group, and the fluorescence images were recorded by a confocal laser scanning microscopy (excitation wavelength: 470 nm for Calcein-AM, 560 nm for PI).

**Detection of intracellular free radical production.** The intracellular production of free radicals was evaluated by flow cytometry using DCFH-DA fluorescent probe, which would change from non-fluorescence to green fluorescence (DCF) in the presence of free radicals. Firstly, 4T1, A549, and HCC-LM3 cancer cells were seeded into 6-well plates at an initial density of 5×10^4^ per milliliter. After being incubated in Lipo-SiaTz (equivalent Tz concentration: 50 μM) containing medium for 2 days, the cells were washed with PBS, and subsequently incubated with CyTCO (10 μM) as well as AIPH (50 μM) for 6 hours. Then DCFH-DA probe (10 μM) was added, and after 30 mins, the cells were irradiated with an 808 nm laser (power density = 1 W cm^-2^) for 5 min. For the control groups, the cells were treated according to the corresponding formulations and stained with DCFH-DA probe. Finally, the cells in each group were detached, and the fluorescence intensity of intracellular activated DCFH was analyzed by flow cytometry.

**Animal experiments.** The male BALB/c mice (4-5 weeks old) were purchased from Guangdong Medical Laboratory Animal Center (GDMLAC, China). The in vivo experiments were approved and conducted in compliance with the regulations of Ethics Committee of Laboratory Animal Center of South China Agricultural University (Approval No. 2023d091). The animals were housed in sterile cages within laminar airflow hoods at 24 °C, 45-65% humidity in a specific pathogen-free room with a 12 h light/12 h dark schedule and fed autoclaved chow and water ad libitum. Mice were randomly allocated to different groups to carry out subsequent experimental investigations. In the case of lethal experimental procedures, mice would be euthanized by exposure to carbon dioxide gas in a rising concentration.

**Biosafety evaluation.** The biosafety of Lipo-SiaTz and pHLipo-CyTCO/AIPH was evaluated by histological examination, body weight measurement, blood routine tests and serum biochemical assays. The mice administrated with PBS (control), Lipo-SiaTz (equivalent SiaTz concentration: 52 mg kg^-1^) or pHLipo-CyTCO/AIPH (equivalent CyTCO/AIPH concentration: 2.4 mg kg^-1^/2.6 mg kg^-1^) were sacrificed 1 week or 1 month post injection. Then, the blood samples and major organs of these mice were collected and analyzed. For blood routine tests, parameters such as hemoglobin (HGB), red blood cells (RBC), white blood cells (WBC), hematocrit (HCT), mean corpuscular volume (MCV), corpuscular hemoglobin (MCH), mean corpuscular hemoglobin concentration (MCHC), red cell distribution width (RDW), and platelets (PLT) were measured. For serum biochemical assays, alanine aspartate aminotransferase (AST), transaminase (ALT), creatinine (CREA), and carbamide (Urea) indicating hepatic and renal function were measured. As for histological examination, the collected organs were embedded in paraffin, sectioned to 4 μm for hematoxylin and eosin (H&E) staining and then observed under a microscope.

**Multispectral optoacoustic imaging.** All in vitro phantom and in vivo mouse multispectral optoacoustic experiments were conducted on the inVision 128 MSOT device. For experiments in phantom, the test solutions were filled in NMR tubes, and then mounted on the device holder. The optoacoustic images of CyTCO mixed with different concentrations of SiaTz were acquired at 850 nm. As for multispectral optoacoustic imaging of cells, the treated cells in culture dishes were washed, trypsinized, centrifuged, suspended in PBS, and fully filled into commercial Wilmad NMR tubes, and then fixed on the holder of the imaging instrument for data collection.

For in vivo multispectral optoacoustic imaging experiments of subcutaneous 4T1 tumor model, the mice pre-administrated with Lipo-SiaTz (equivalent SiaTz concentration: 52 mg kg^-1^) were anesthetized by 1% isoflurane delivered via a nose cone, and then intravenously injected with pHLipo-CyTCO/AIPH (equivalent CyTCO/AIPH concentration: 2.4 mg kg^-1^/2.6 mg kg^-1^). The tumor region of each mouse was imaged at predetermined time intervals (0, 2, 4, 6, 8, 10, 12, 24 and 48 h). As for the orthotopic liver tumor and lymphatic metastasis model, the mice which was previously administered Lipo-SiaTz were intravenously injected with pHLipo-CyTCO/AIPH. After 12 h, these mice were placed in the prone position, and then scanned to acquire MSOT images. During the data collection process, 680 nm, 700 nm, 730 nm, 760 nm, 800 nm (background), 850 nm, 875 nm and 900 nm were selected considering the major inflection points corresponding to the absorption curves of CyNH (the Tz-activated form of CyTCO), oxyhemoglobin and deoxyhemoglobin, and 10 individual frames were acquired for each wavelength. Subsequently, spectral unmixing was performed by using a linear regression algorithm to extract optoacoustic signals generated from CyNH (the Tz-activated form of CyTCO). Orthogonal MIP MSOT images were generated by stacking cross-sectional images along the z-axis. Cryosection images provided with *viewMSOT* software in the MSOT system were used for the reference to anatomical details.

**In vivo antitumor efficacy.** For the mouse model of subcutaneous tumor, 2 × 10^6^ 4T1 cancer cells suspended in a Matrigel/PBS (3:2) mixture (50 μL) were subcutaneously injected onto the back of male BALB/c nude mice. When the tumor volumes reached around 60 mm^3^, the mice were randomly divided into 4 groups. All the procedures including drug administration, optoacoustic imaging and photothermal/thermodynamic combined therapy were strictly conducted according to the timeline shown in Figure 4. Tumor volume was calculated as follows: tumor volume (V) = (tumor length) × (tumor width)^2^/2.

The orthotopic liver tumor mouse model was established by surgical intrahepatic injection of HCC-LM3-fLuc cancer cells (2×10^6^ cells for each mouse). Generally, the male mice were anesthetized, and a midline incision (about 1-1.5 cm) was made to expose the left lobe of the liver. After HCC-LM3-fLuc cells suspended in Matrigel/PBS (3:2) mixture (50 μL) were injected slowly into the left lobe of liver at a 30° angle using a sterile 30G needle, a transparent bleb could be observed at the point of injection under the liver capsule. Then, gentle pressure was applied for about 2 min with a sterile cotton swab to prevent bleeding. Finally, the left lobe of the liver was gently squeezed back into the abdominal cavity and the incision was closed using a sterile 6-0 biodegradable surgical silk suture. The mice were observed for a few hours and then returned to the animal housing facilities. For sham surgery group, the mice were only injected with equal volume of Matrigel/PBS mixture without HCC-LM3-fLuc cells. When the bioluminescence intensity at the liver area reached around 1.3 × 10^6^ p s^-1^ cm^-2^ sr^-1^, the mice were randomly divided into 4 groups. All the procedures including drug administration, optoacoustic imaging and photothermal/thermodynamic combined therapy were strictly conducted according to the timeline shown in Figure 5.

The lymphatic metastasis mouse model was established by injecting 2.5 × 10^5^ 4T1-fLuc cancer cells suspended in PBS (pH 7.4, 20 µL) into the right hind footpads of male mice and incubated for 21 days before experiments. When the bioluminescence intensity at the popliteal lymph node region reached around 3.7 × 10^6^ p s^-1^ cm^-2^ sr^-1^, the mice were randomly divided into 4 groups. All the procedures including drug administration, optoacoustic imaging and photothermal/thermodynamic combined therapy were strictly conducted according to the timeline shown in Figure 6.

**TUNEL assays**. The excised tumors were fixed in 10% buffered formalin and embedded in paraffin. Sections of 5 μm were affixed to slides and then deparaffinized and rehydrated. Endogenous peroxidase activity was quenched in 3% H_2_O_2_ in methanol for 15 min. The slides were incubated with terminal deoxynucleotidyl transferase (TdT) solution at 37 °C for 1h. After washed with PBS, the slides were successively incubated with Streptavidin-FITC and DAPI solution for another 60 and 5 min. Finally, the slides were cover-slipped and then observed under an optical microscope.

**Western blot analysis.** After intravenous injection of Lipo-SiaTz (equivalent SiaTz concentration: 52 mg kg^-1^) into tumor-bearing mice for three consecutive days, the tumors were excised and rinsed in cold sterile PBS and then homogenized in ice-cold RIPA Lysis Buffer (1/10, w/v, Keygen Biotech) with protease inhibitor cocktails (0.1 %) and PMSF (1 mM, Keygen Biotech). Homogenates were centrifuged at 12000 g for 5 min at 4 °C, and supernatants were assayed for protein content using a BCA Protein Assay Kit (Keygen Biotech) according to instructions provided. The lysate was incubated with TCO-PEG_4_-biotin (0.25 mM) (Broadpharm) for 6 h at 37 °C. Next SDS-PAGE loading buffer was added into the protein supernatants (1/4, v/v, Keygen Biotech) and then the mixture was boiled for 10 min (95 °C). The protein samples were separated by electrophoresis on SDS-PAGE gels and transferred to PVDF membranes. The PVDF membranes were blocked with the Western Blocking Buffer (Keygen Biotech) at room temperature for 1 h. After washed by TBST, the membranes were incubated with HRP-Streptavidin (Sigma-Aldrich) at 1:2000 dilution for 2 h. Finally, the membranes were rinsed with TBST 5 times, and then were subject to chemiluminescence detection (ECL Kit, Keygen Biotech) by Tanon 5200 Imaging System.


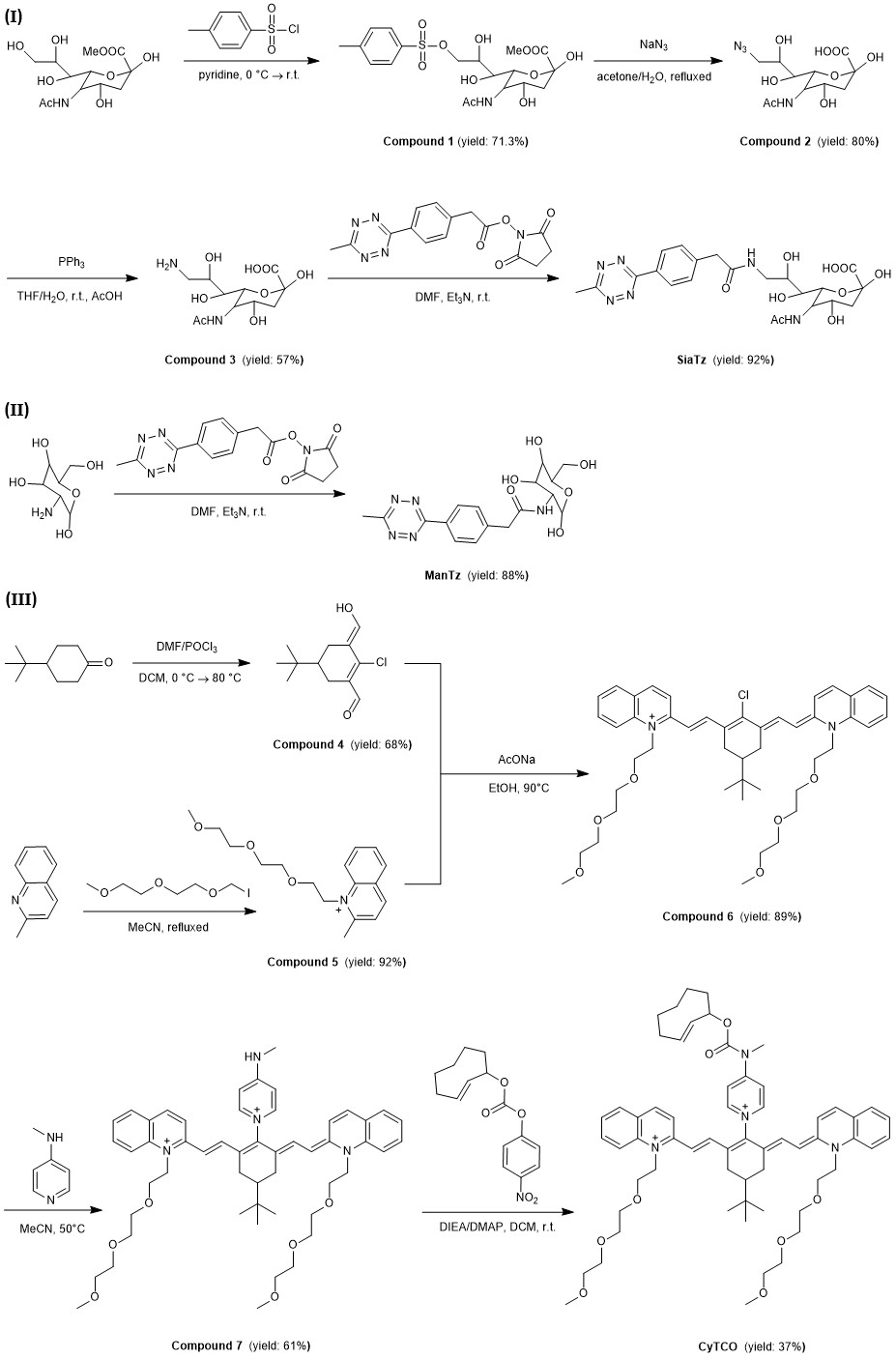


**Figure S1.** Synthesis route of (Ⅰ) SiaTz, (II) ManTz, and (III) CyTCO.


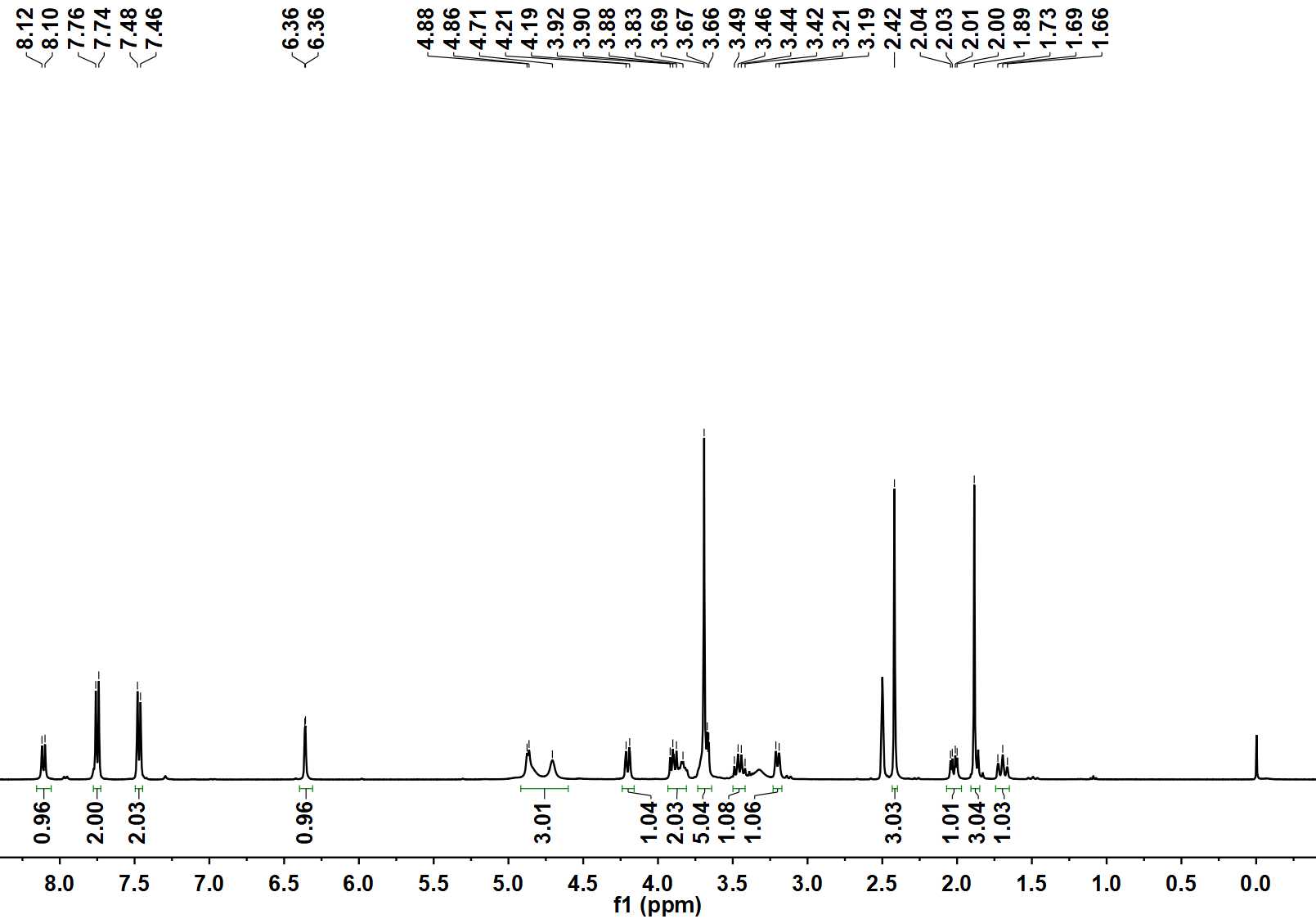


**Figure S2.** ^1^H NMR spectrum of compound **1** in DMSO-*d*_6_.


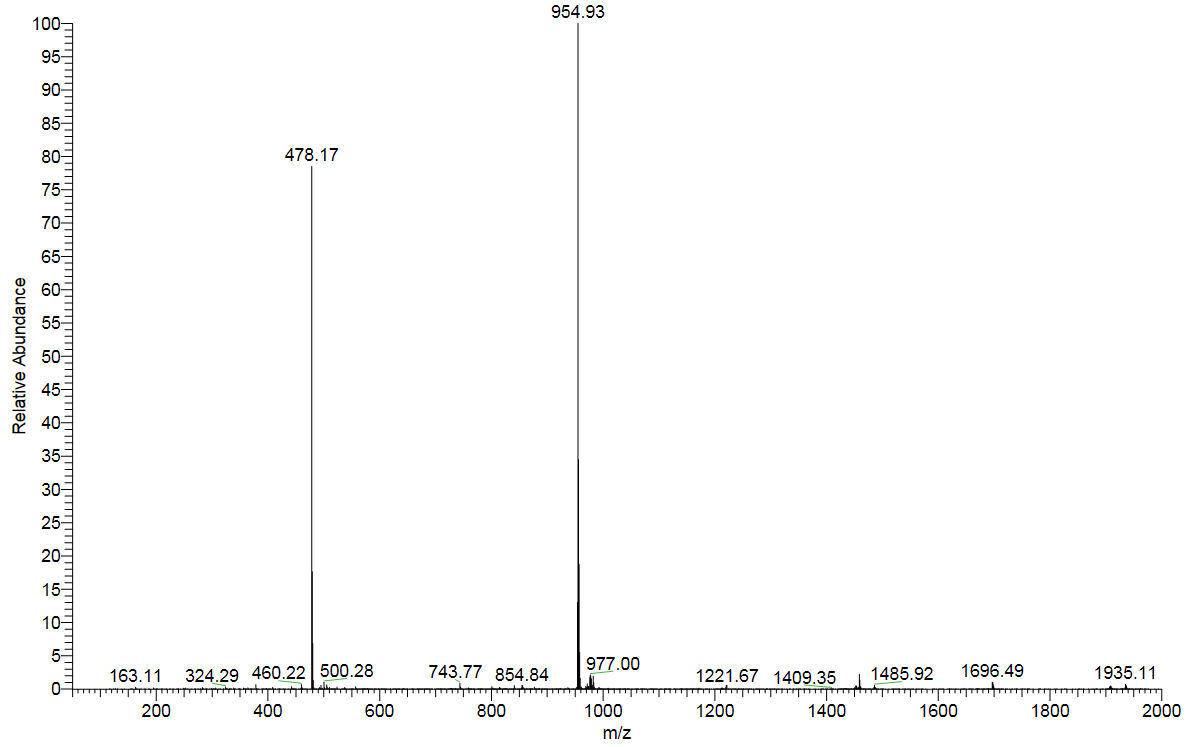


**Figure S3.** Liquid chromatography-mass spectrum of compound **1.**


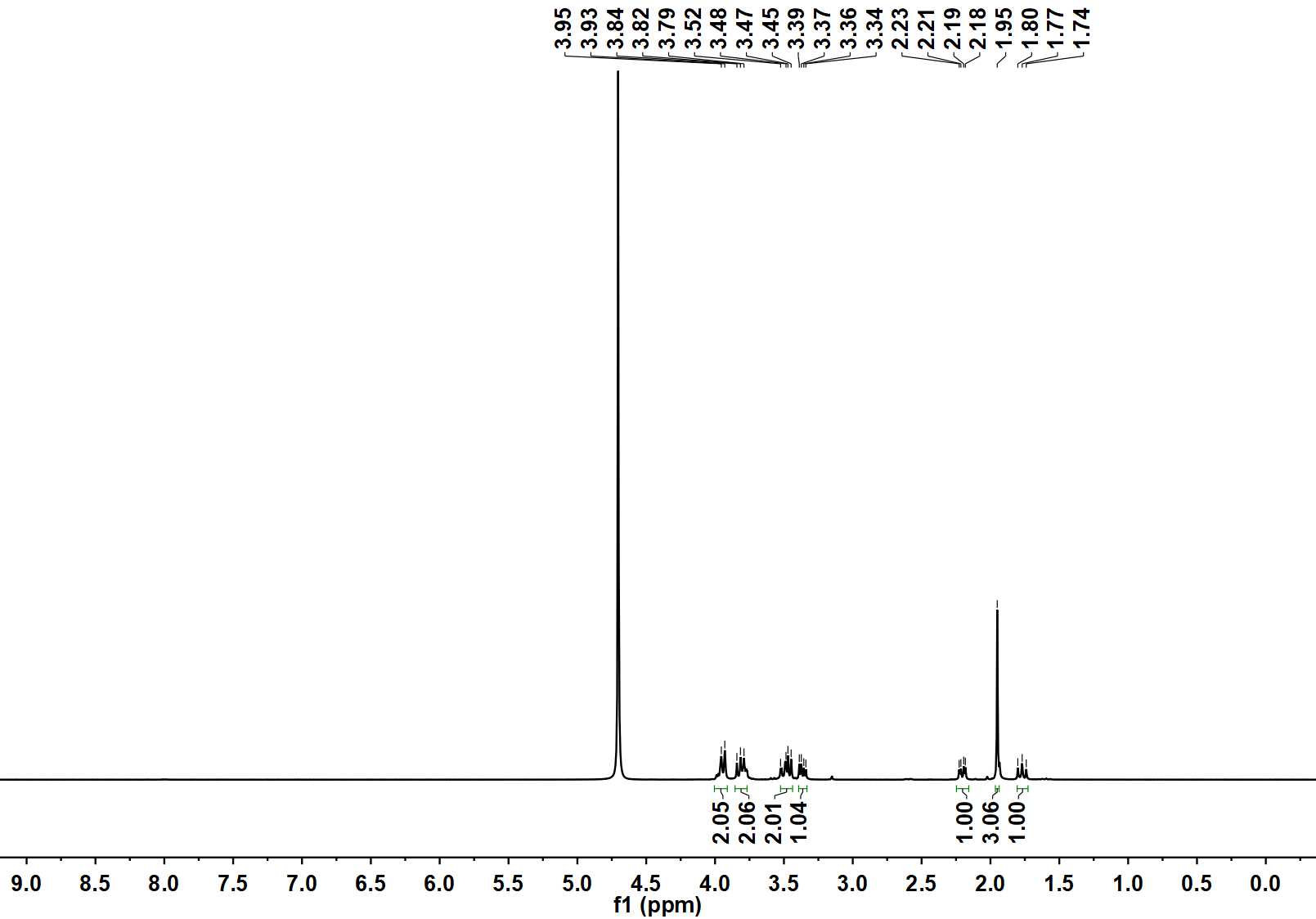


**Figure S4**. ^1^H NMR spectrum of compound **2** in D_2_O.


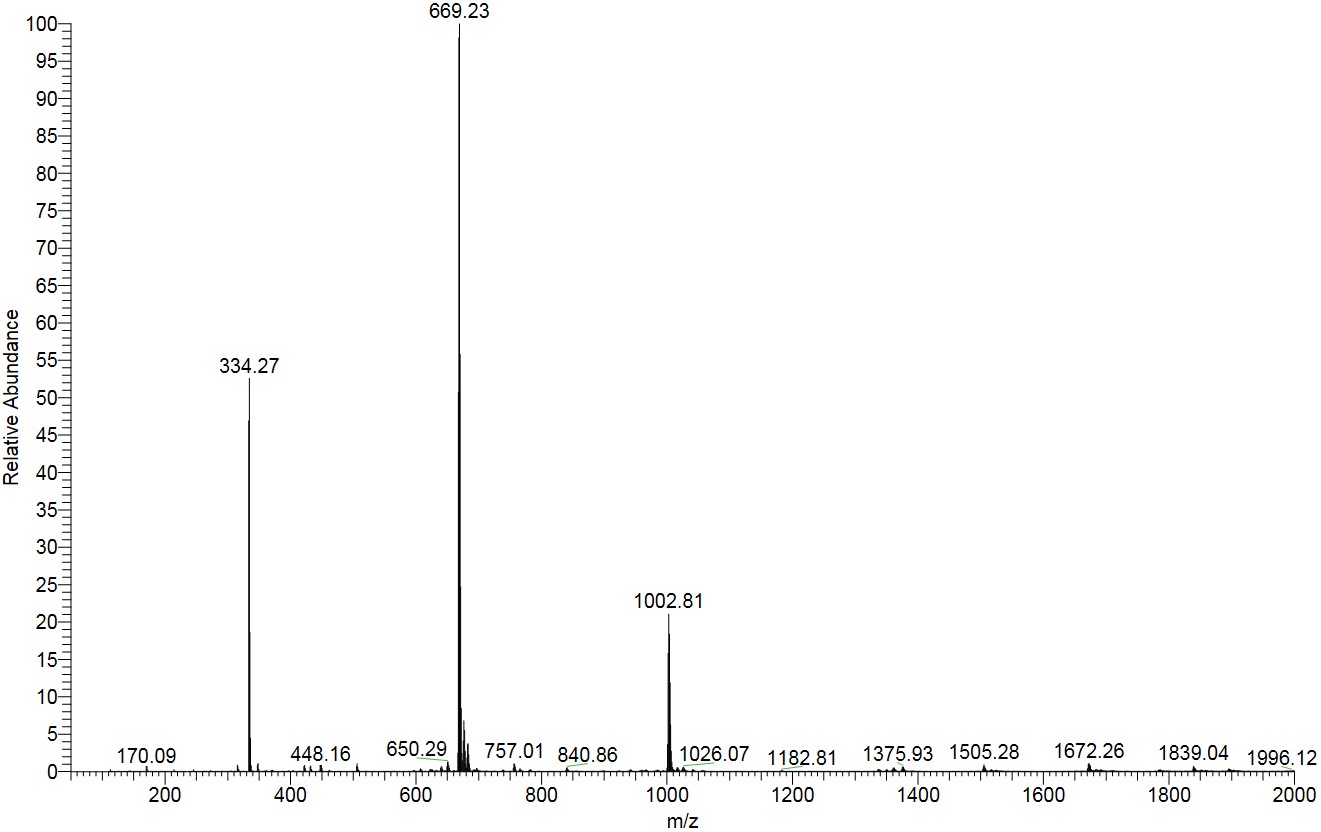


**Figure S5.** Liquid chromatography-mass spectrum of compound **2.**


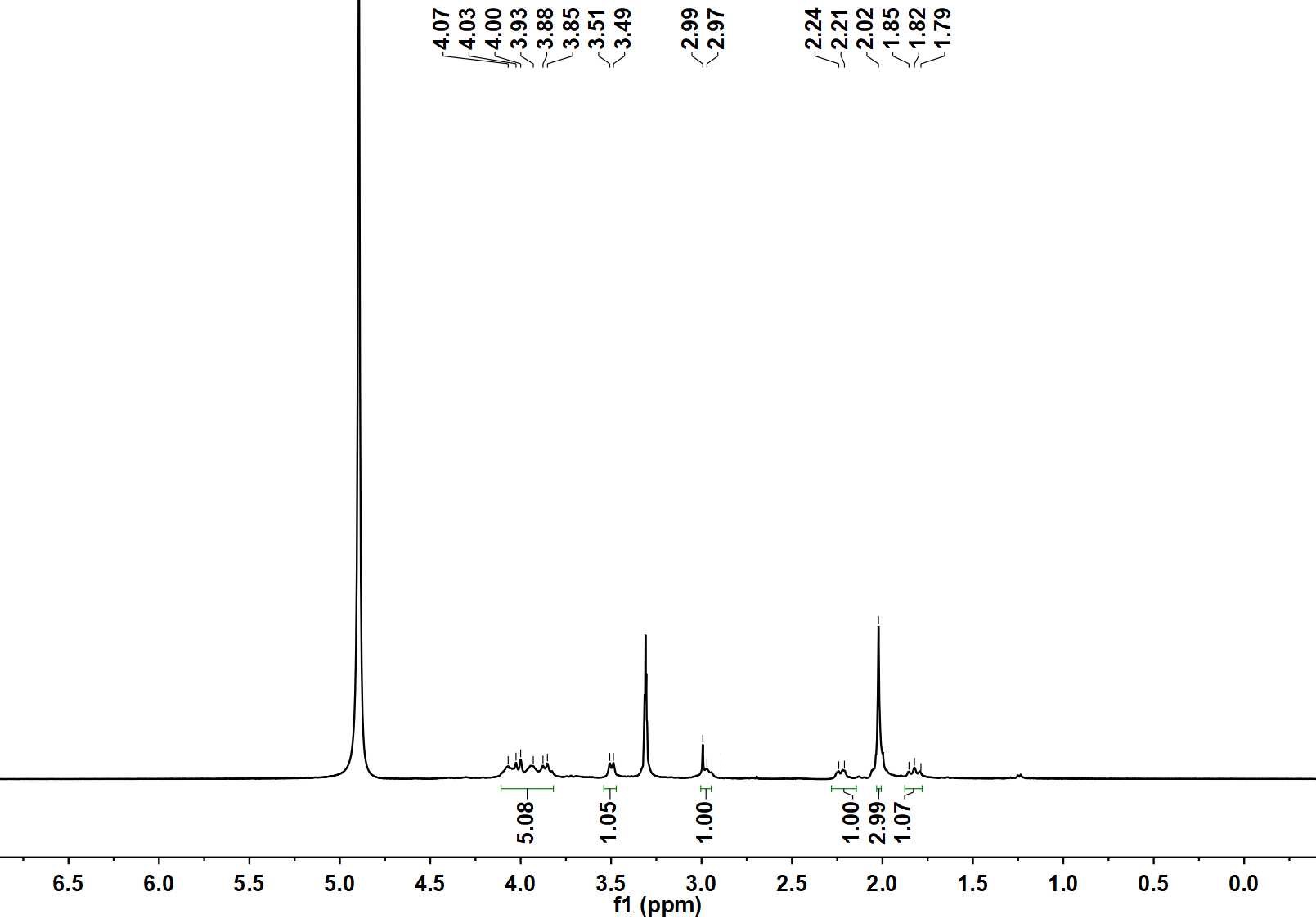


**Figure S6.** ^1^H NMR spectrum of compound **3** in MeOD*-d*_4_.


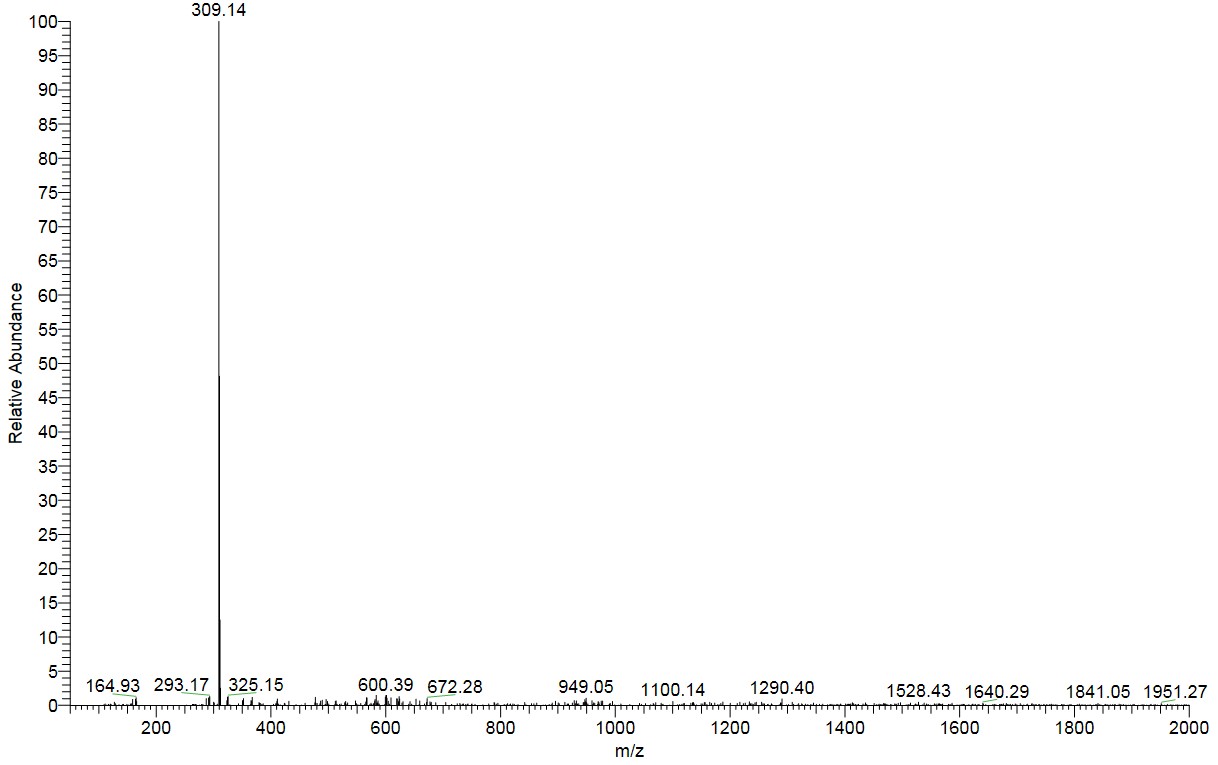


**Figure S7.** Liquid chromatography-mass spectrum of compound **3.**


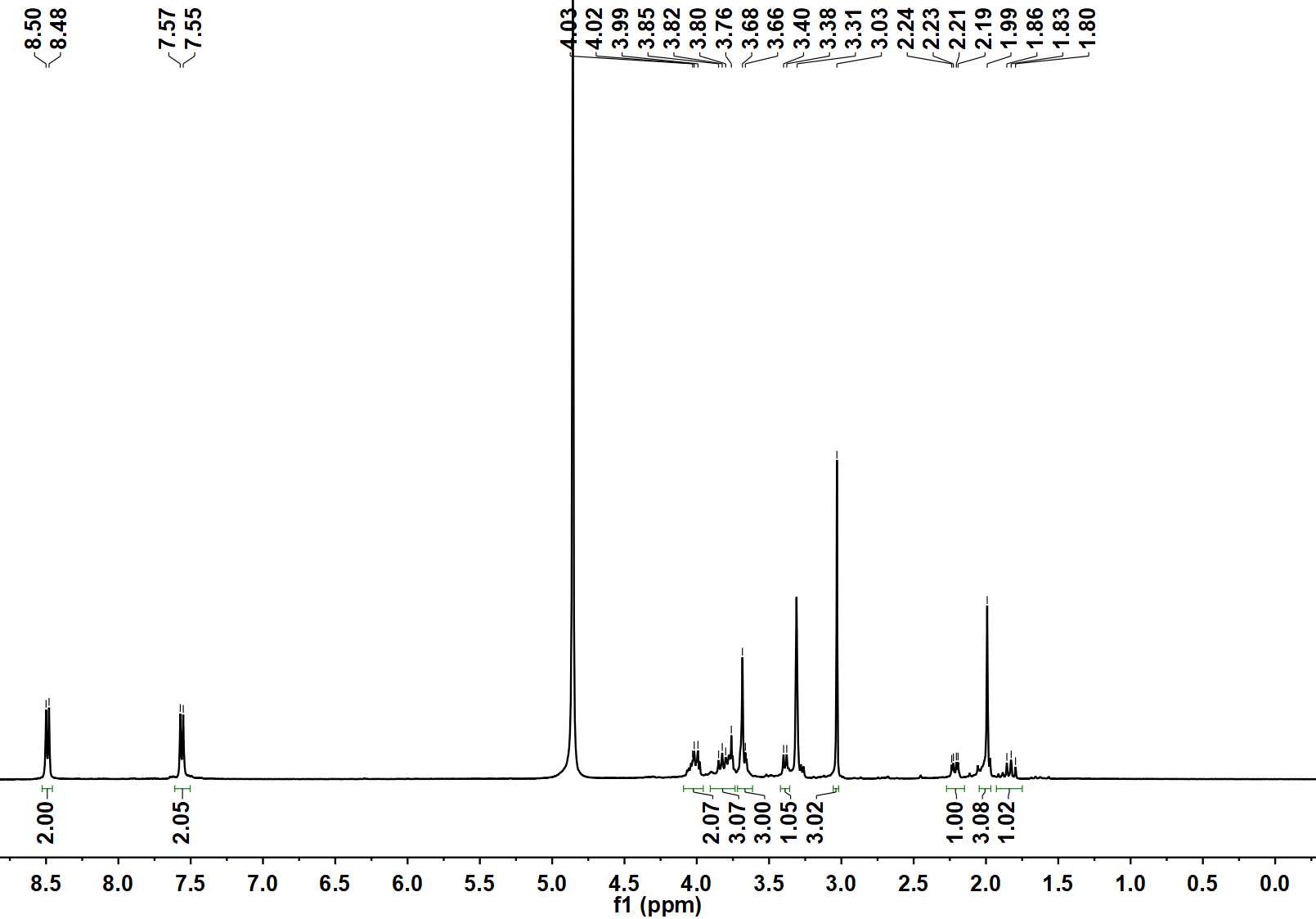


**Figure S8.** ^1^H NMR spectrum of SiaTz in MeOD*-d*_4_.


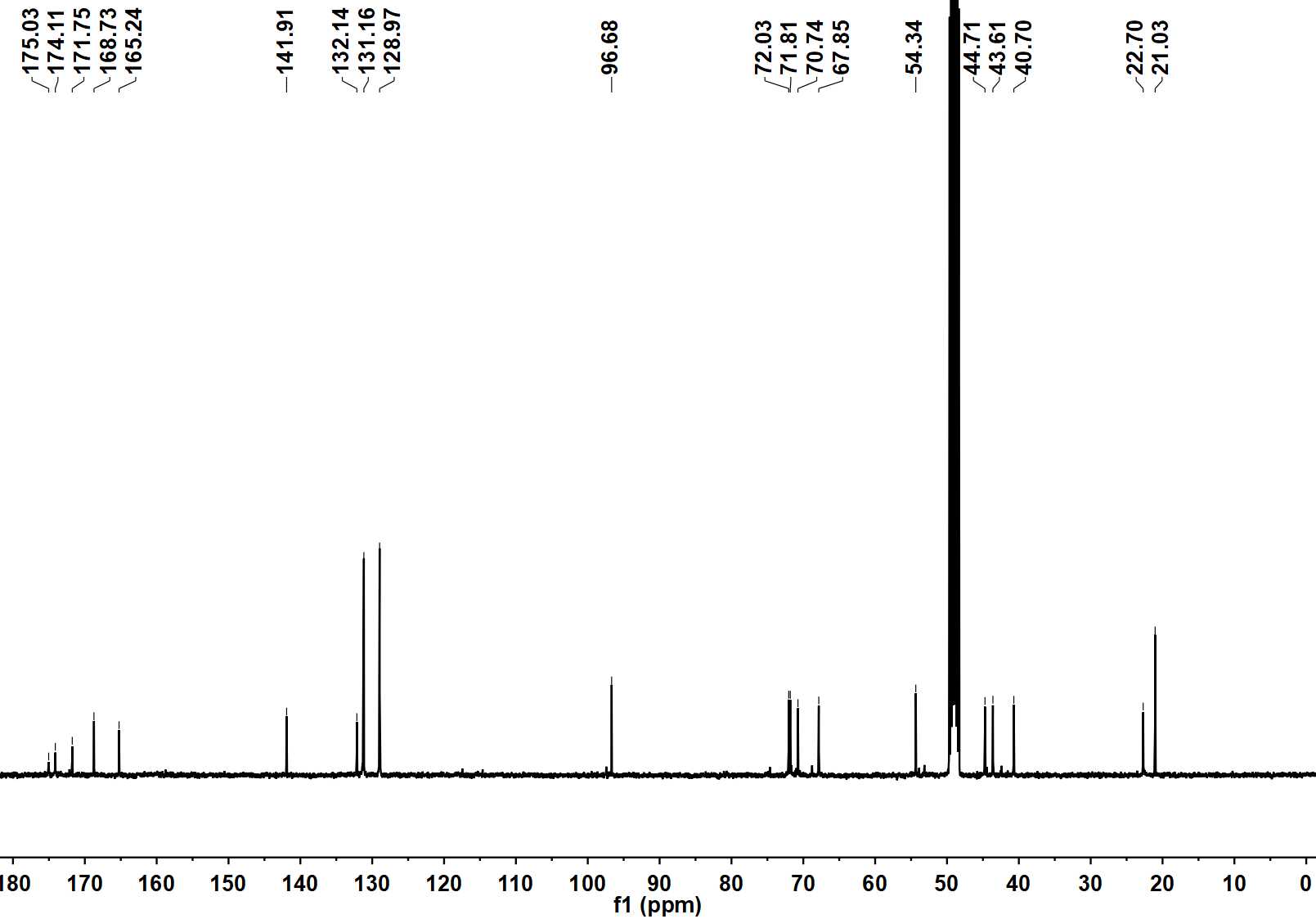


**Figure S9.** ^13^C NMR spectrum of SiaTz in MeOD-*d*_4_.


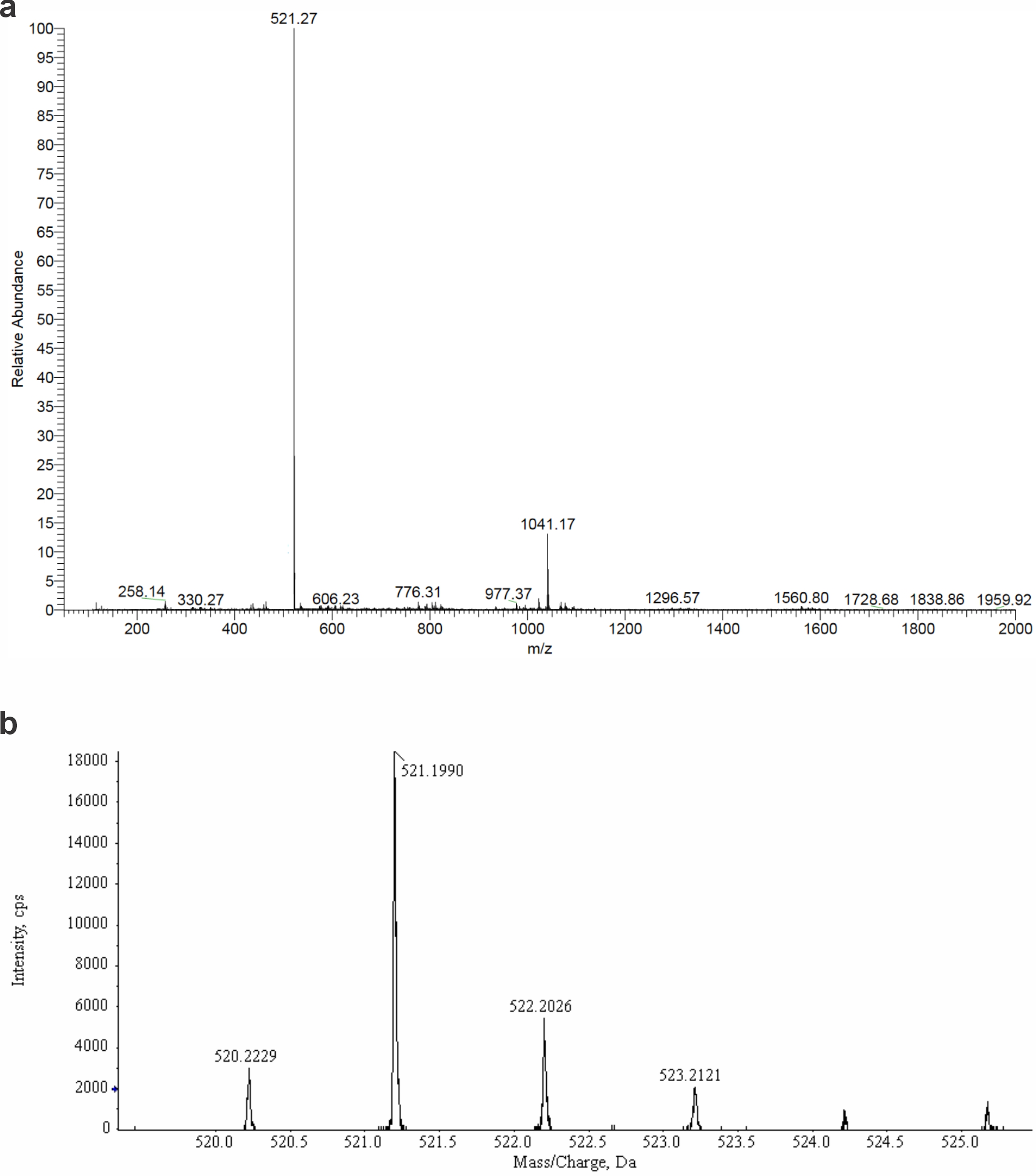


**Figure S10. (a)** Liquid chromatography and **(b)** high-resolution mass spectrum of SiaTz.


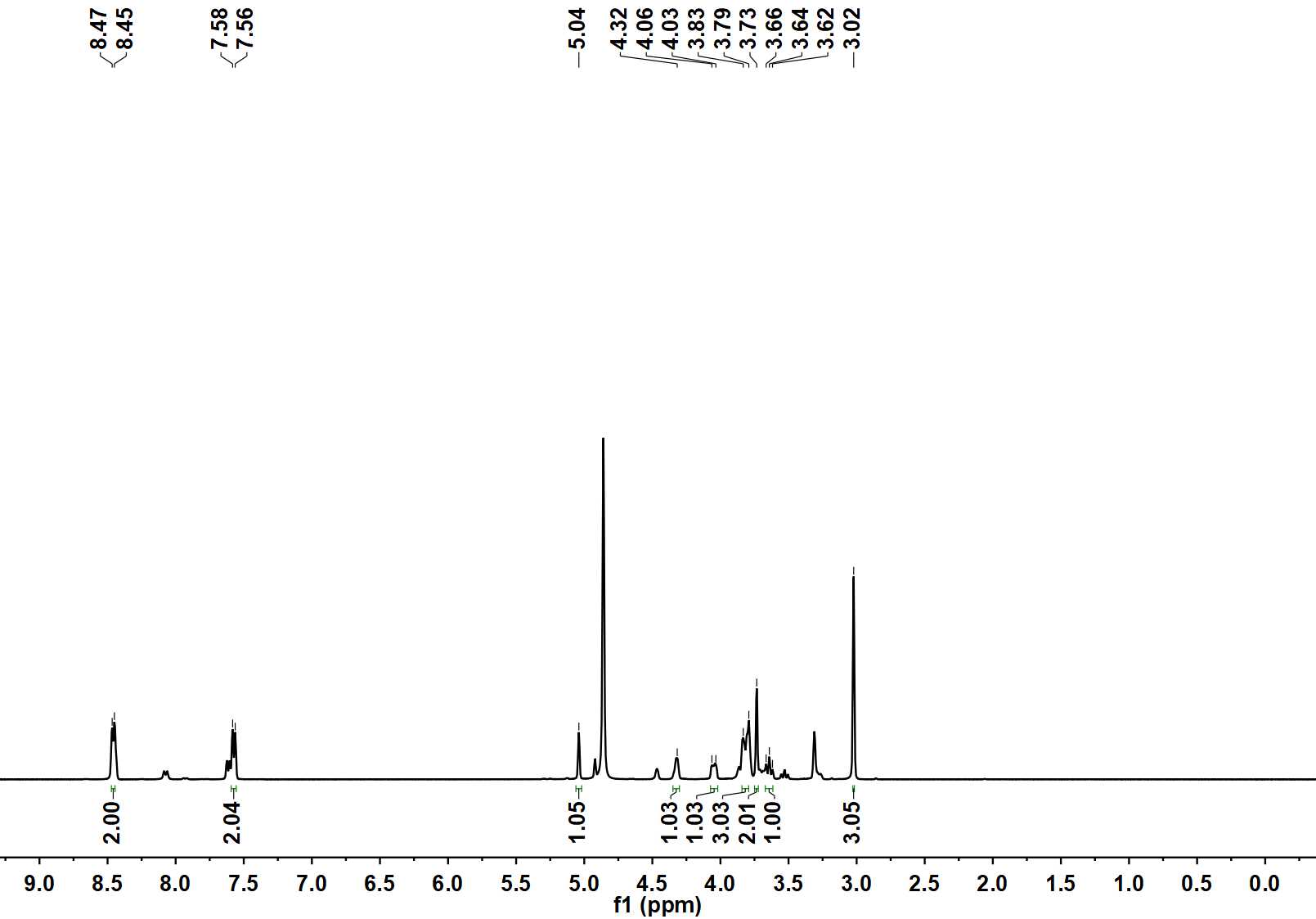


**Figure S11.** ^1^H NMR spectrum of ManTz in MeOD-*d*_4_.


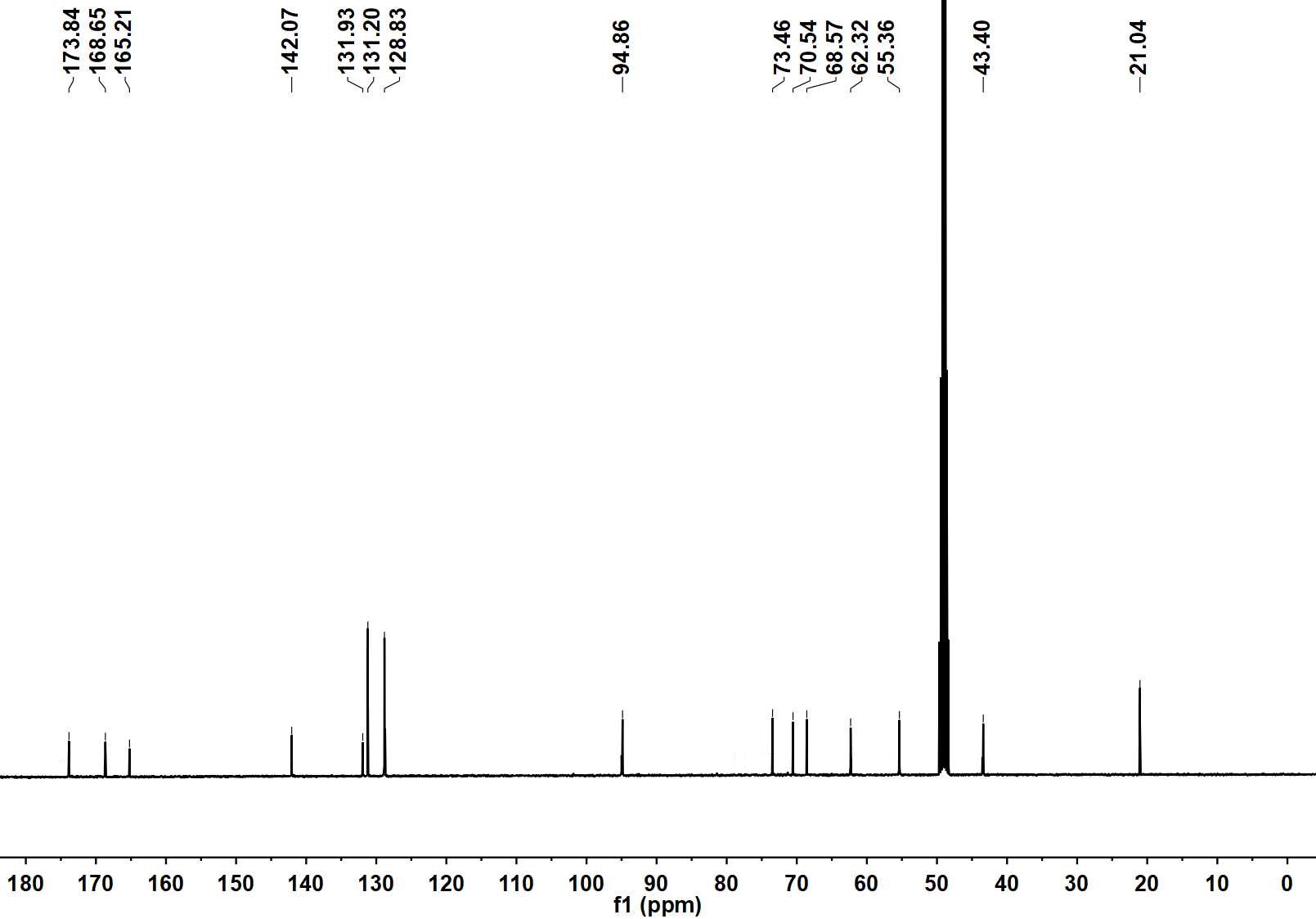


**Figure S12.** ^13^C NMR spectrum of ManTz in MeOD-*d*_4_.


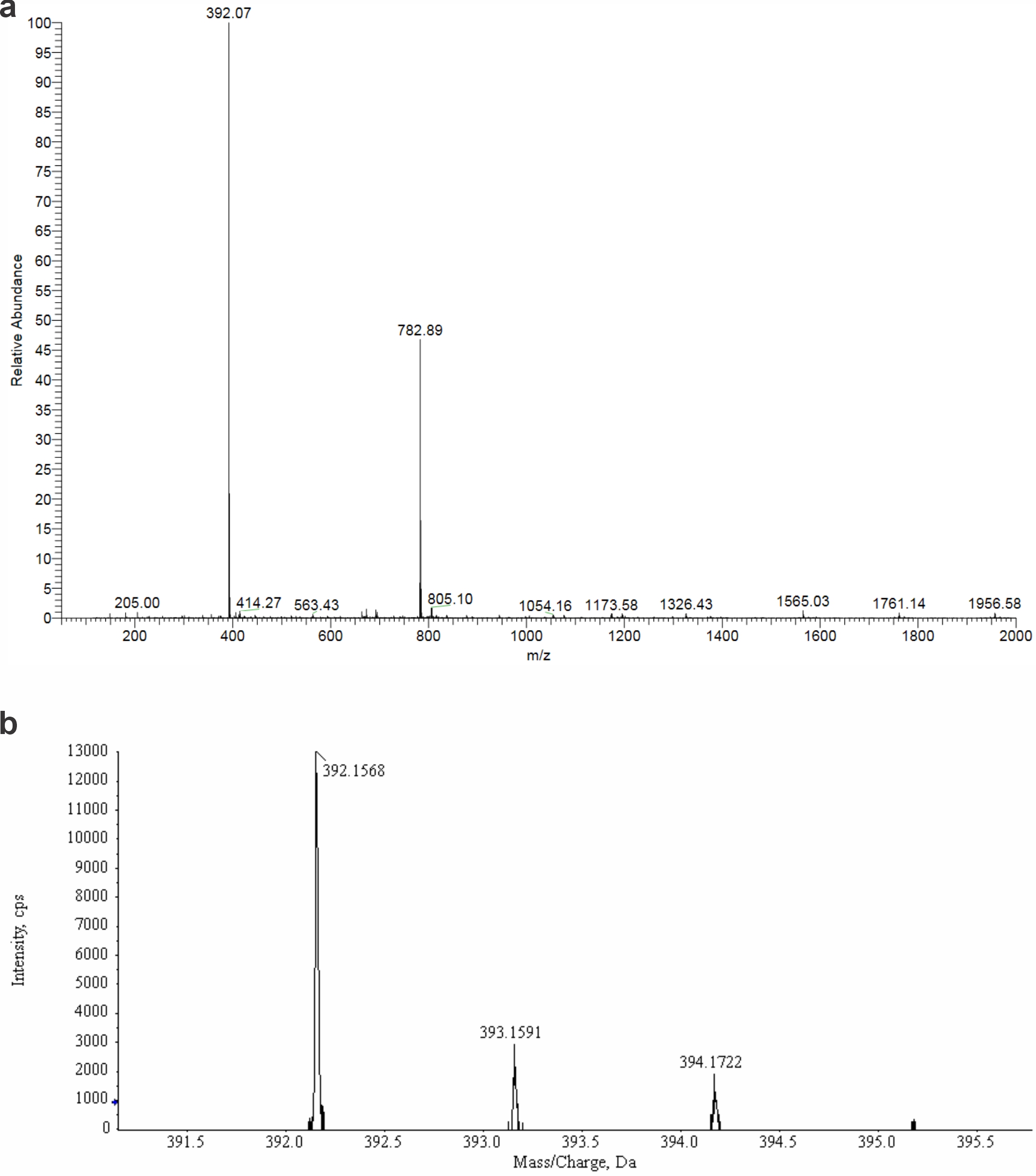


**Figure S13. (a)** Liquid chromatography and **(b)** high-resolution mass spectrum of ManTz.


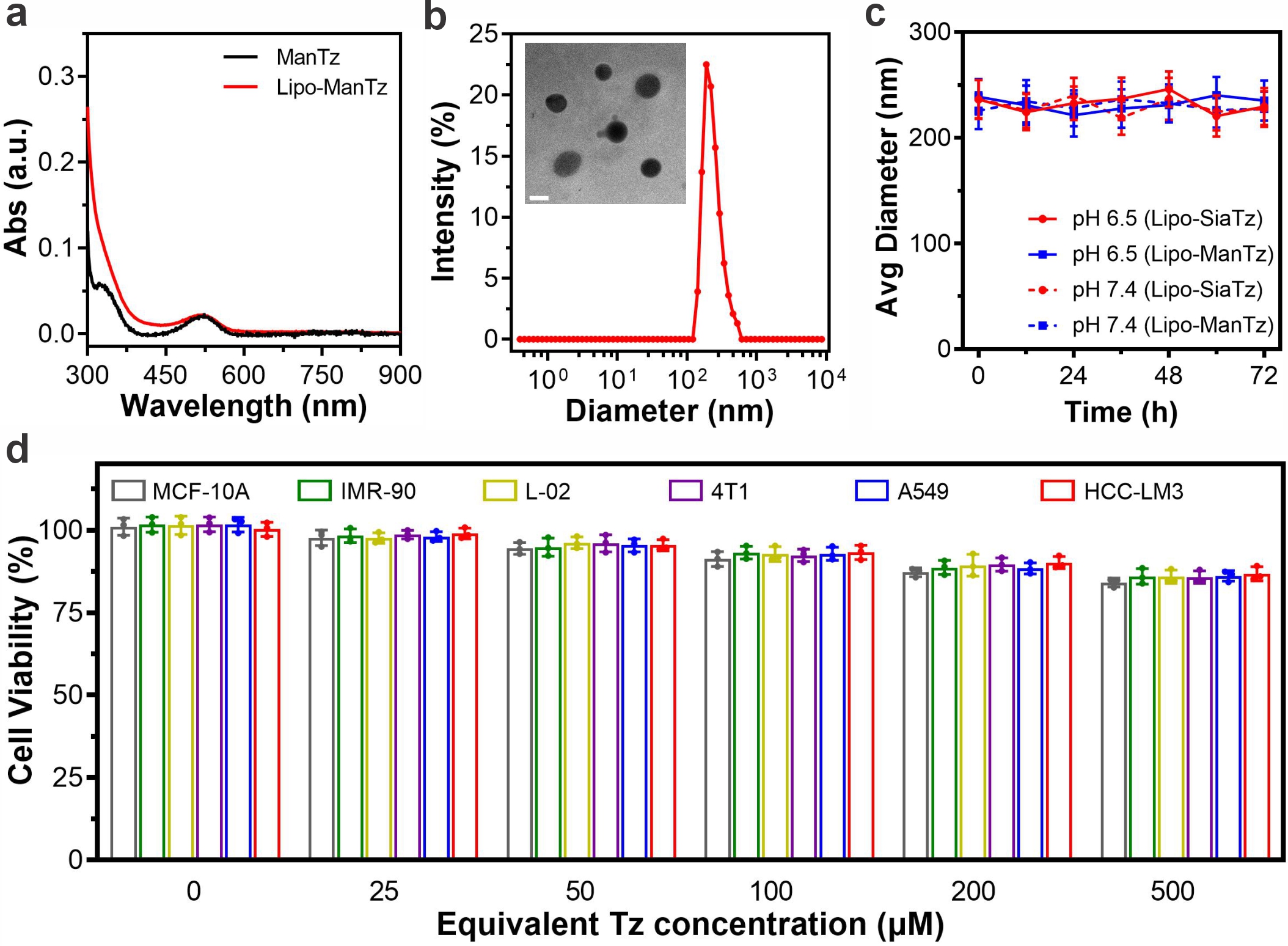


**Figure S14. Properties of ManTz and Lipo-ManTz. (a)** Absorption spectra of ManTz (10 μM) and Lipo-ManTz (equivalent Tz concentration: 10 μM). **(b)** Hydrodynamic diameter distribution and representative TEM image for a sample of Lipo-ManTz. Scale bar: 200 nm. **(c)** Changes in average diameters of Lipo-SiaTz and Lipo-ManTz (equivalent Tz concentration: 50 μM) after storage in CPBS (10 mM, pH = 6.5 or 7.4) for different times. **(d)** Viabilities of MCF-10A, IMR-90, L-02, 4T1, A549 and HCC-LM3 cells upon 48 hours of incubation with Lipo-ManTz at different concentrations (equivalent Tz concentration: 0, 25, 50, 100, 200, 500 μM) (n = 3 independent experiments). Data were represented as mean values ± standard deviation (SD).


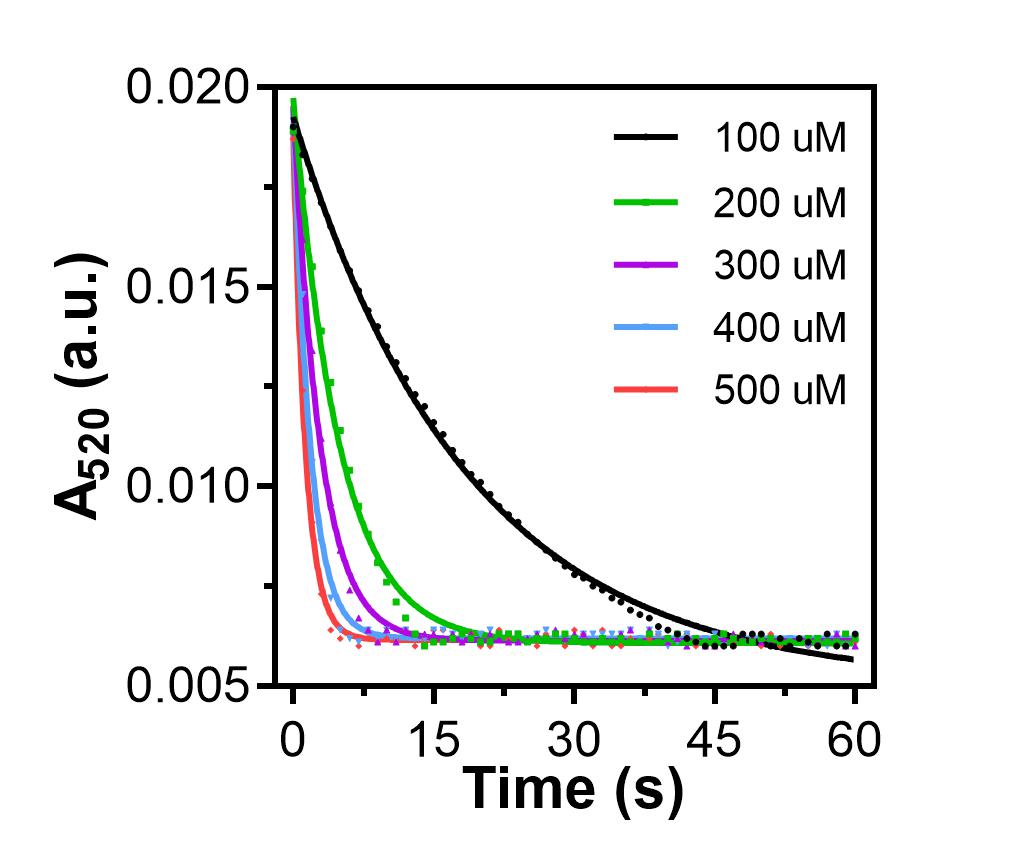


**Figure S15. Tetrazine–*trans*-Cyclooctene kinetics.** Data points and fitted lines for the reactions between SiaTz (10 μM) and different concentrations of TCO-amine in CPBS (10 mM, pH = 6.5) containing 5% DMSO by monitoring the absorbance at 520 nm.


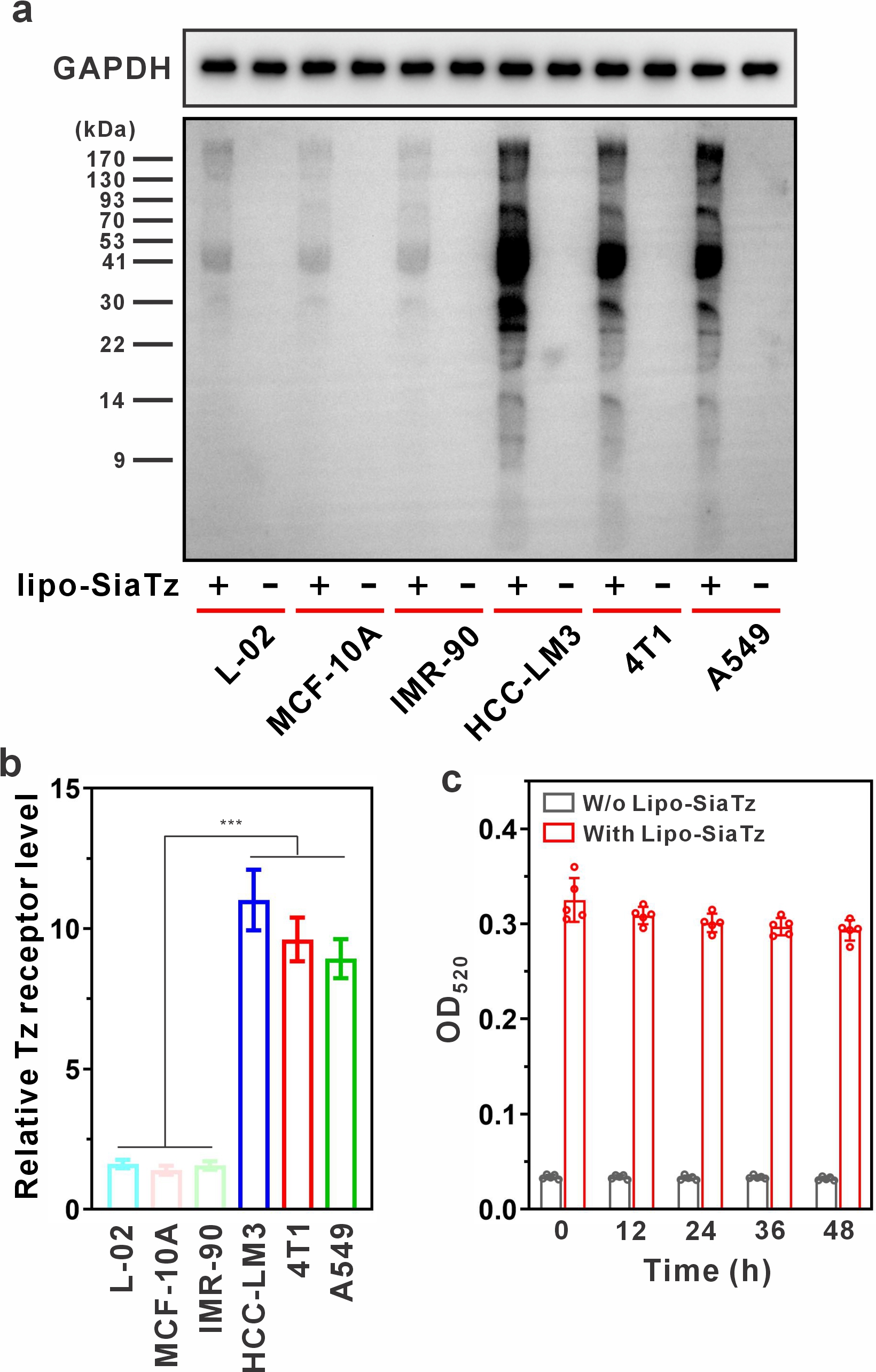


**Figure S16. Analysis of the Tz triggers expressed on the cell surfaces. (a)** Western blot images of normal (L-02, MCF-10A and IMR-90) and cancer (HCC-LM3, 4T1 and A549) cells upon 48 h of incubation without (-) or with (+) Lipo-SiaTz (equivalent Tz concentration: 75 μM). GAPDH (upper panel) was selected as the internal reference. **(b)** Relative expression levels of Tz triggers in different cells after incubation with Lipo-SiaTz. The quantitative results were obtained from **(a)** by using ImageJ software (n = 3 independent experiments). **(c)** Changes in optical density at 520 nm of the HCC-LM3 cells pre-treated with Lipo-SiaTz after incubation for 0, 12, 24, 36 and 48 h (n = 5 independent experiments). Data were represented as mean ± standard deviation (SD). Statistical significance was determined by two-tailed t test. ^***^P < 0.001.


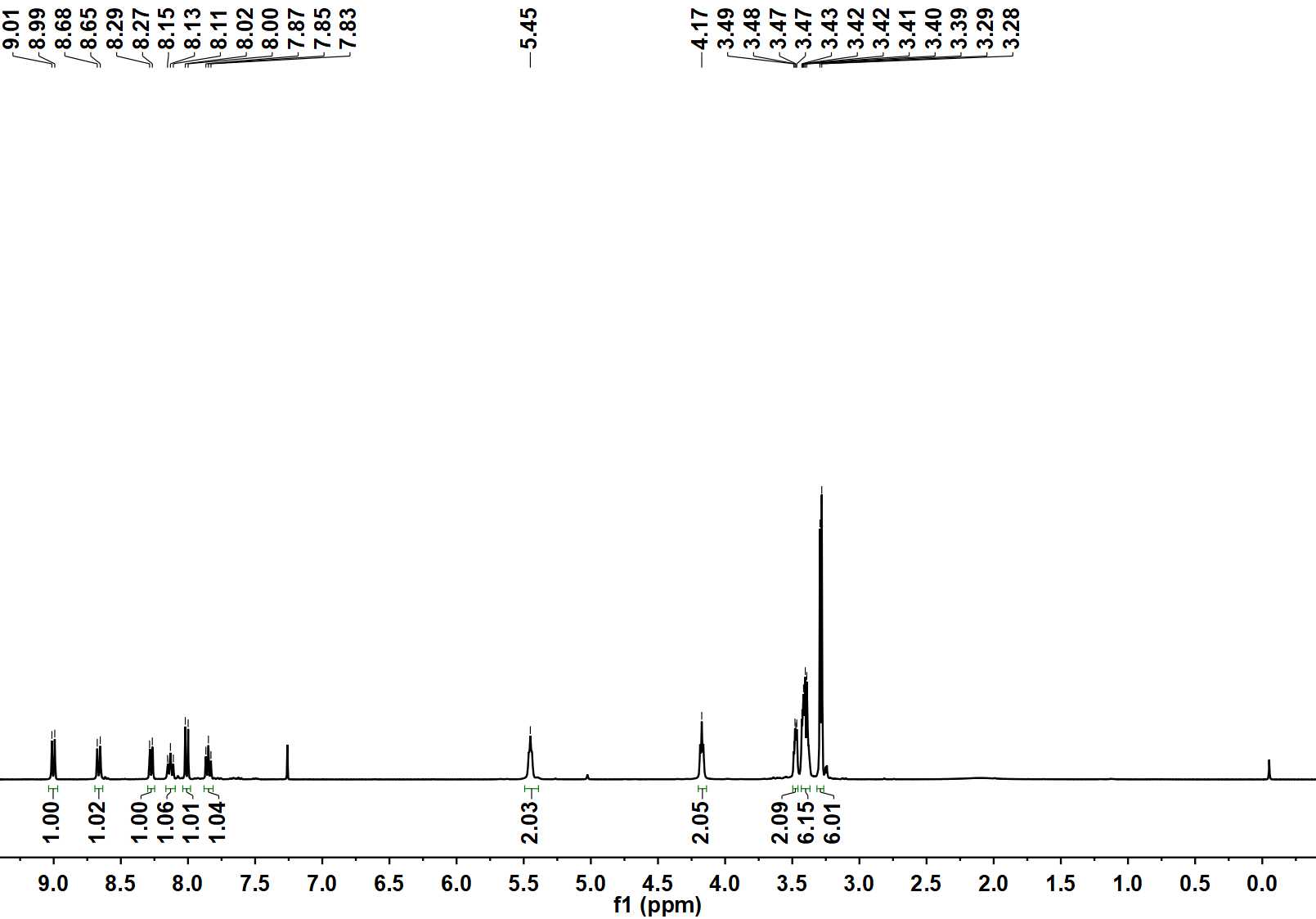


**Figure S17.** ^1^H NMR spectrum of compound **5** in CDCl_3_.


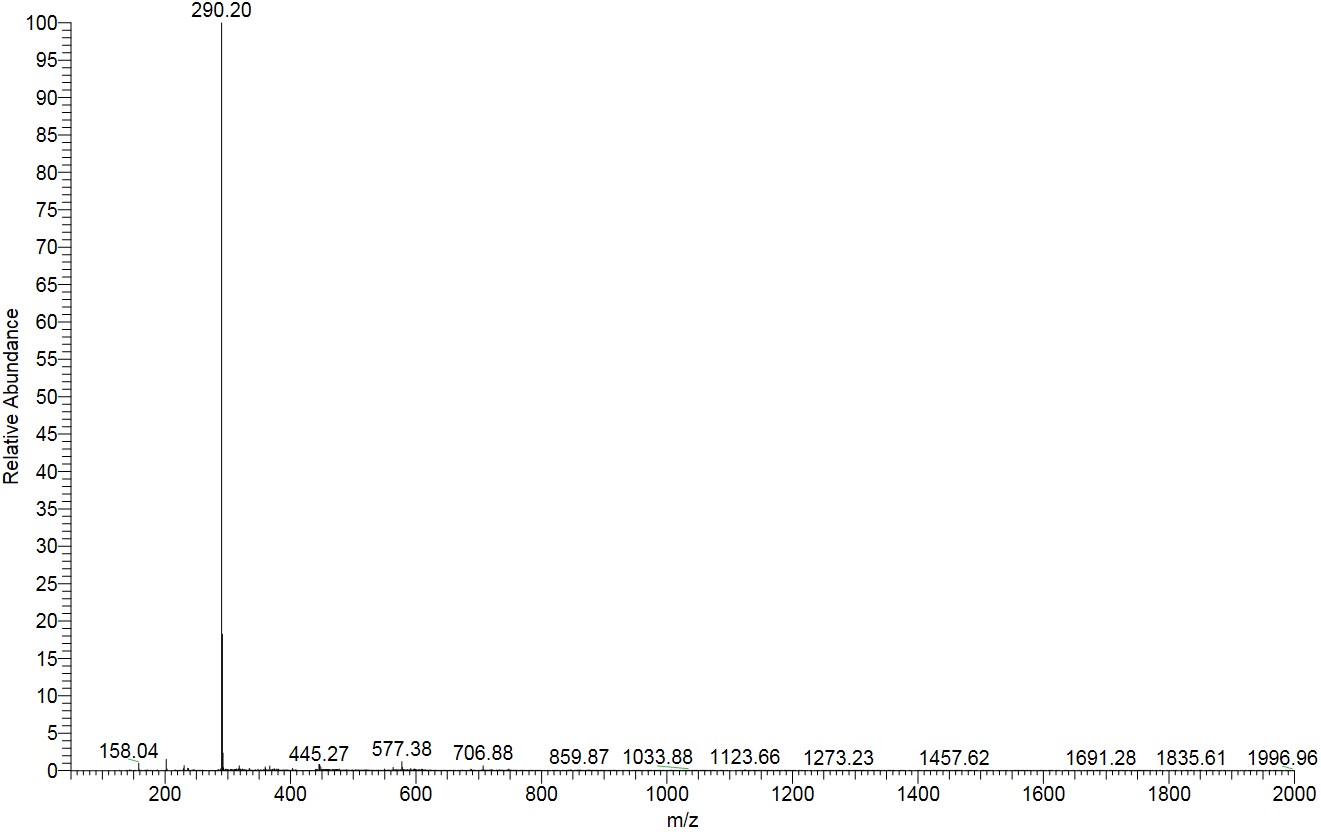


**Figure S18.** Liquid chromatography-mass spectrum of compound **5**.


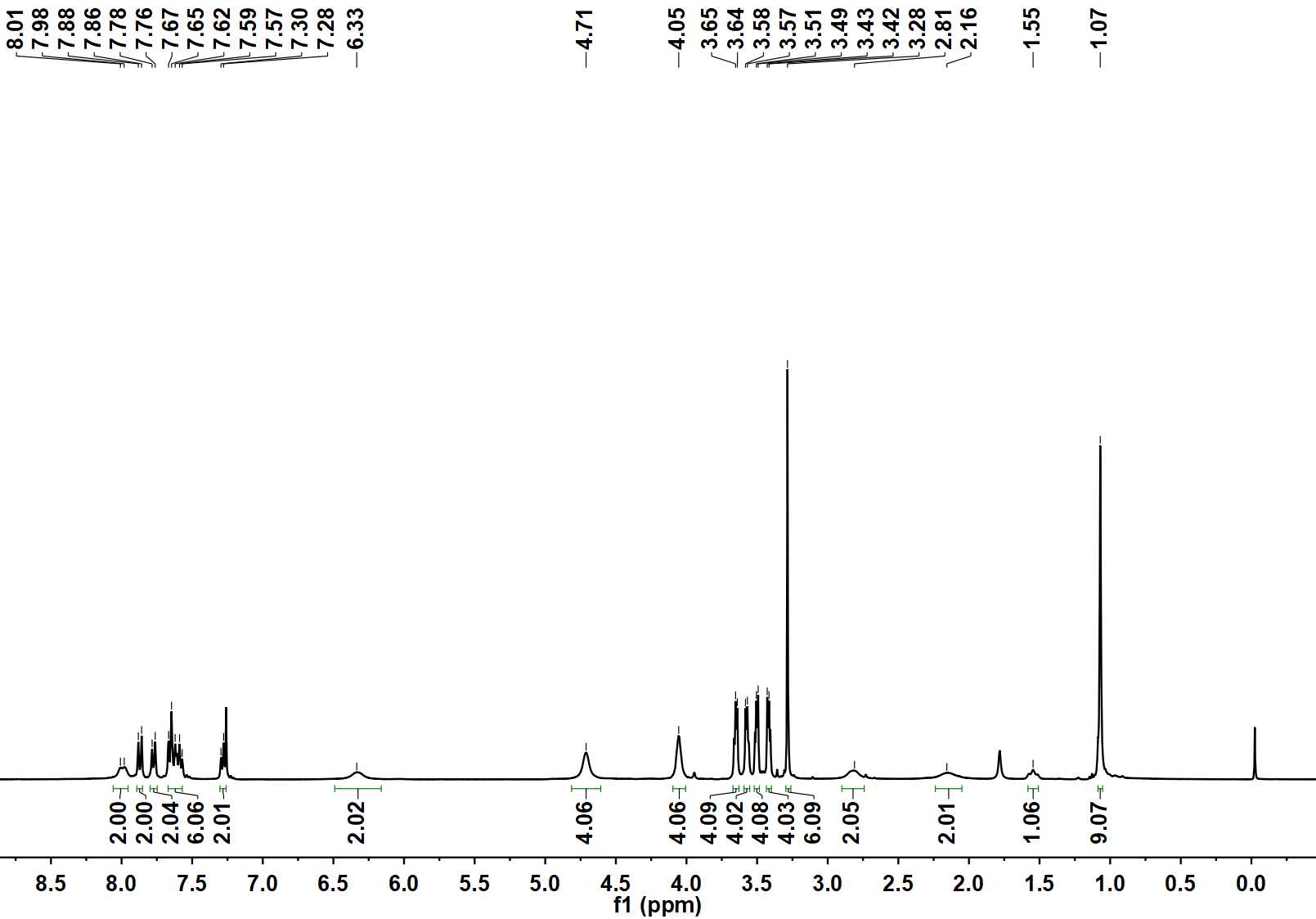


**Figure S19.** ^1^H NMR spectrum of compound **6** in CDCl_3_.


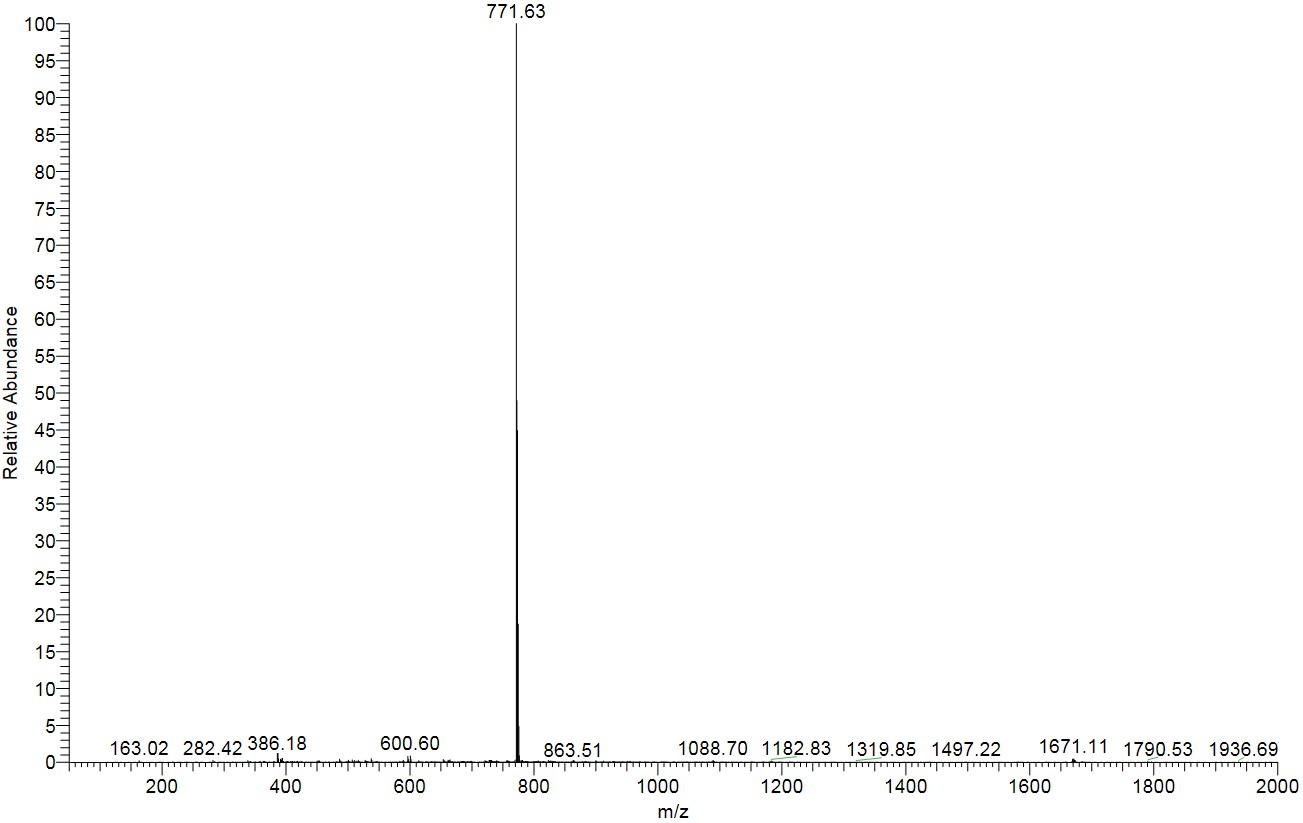


**Figure S20.** Liquid chromatography-mass spectrum of compound **6**.


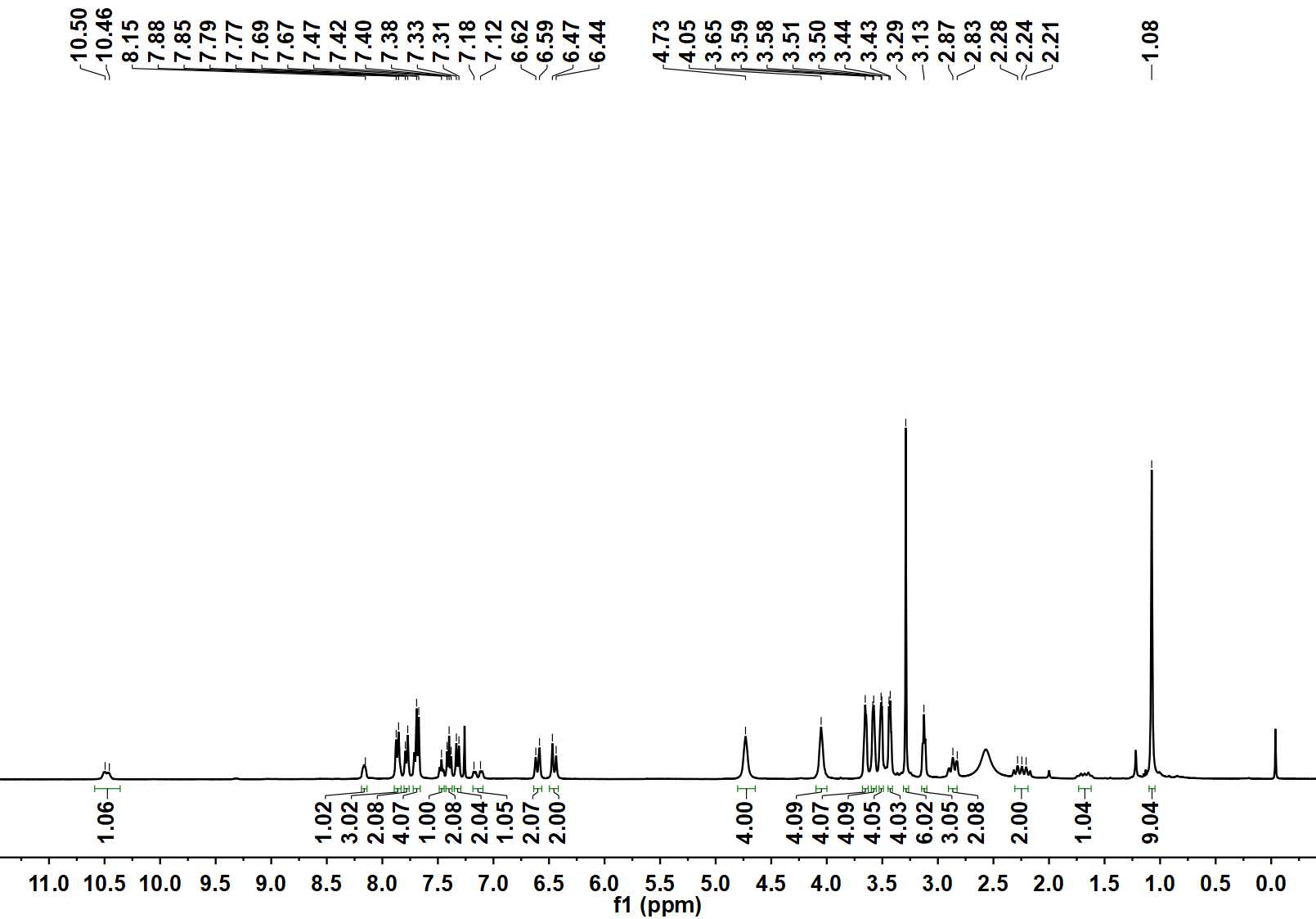


**Figure S21.** ^1^H NMR spectrum of compound **7 (CyNH)** in CDCl_3_.


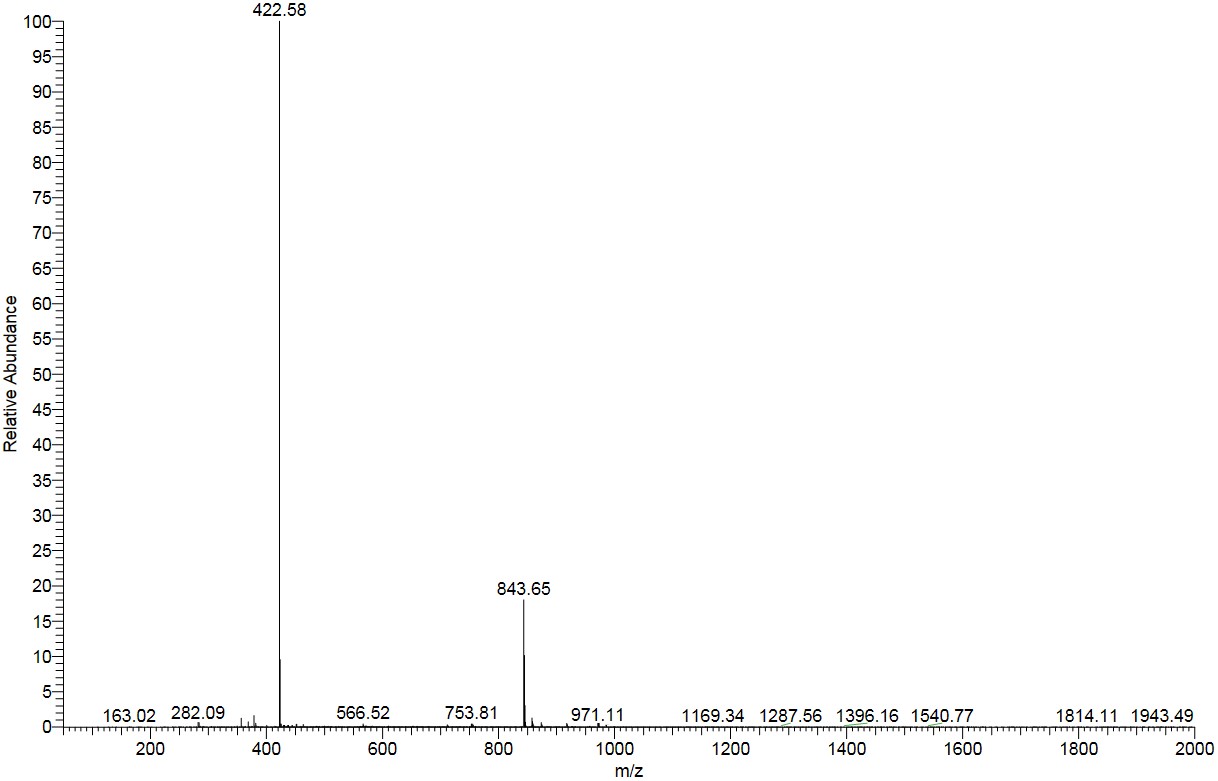


**Figure S22.** Liquid chromatography-mass spectrum of compound **7 (CyNH)**.


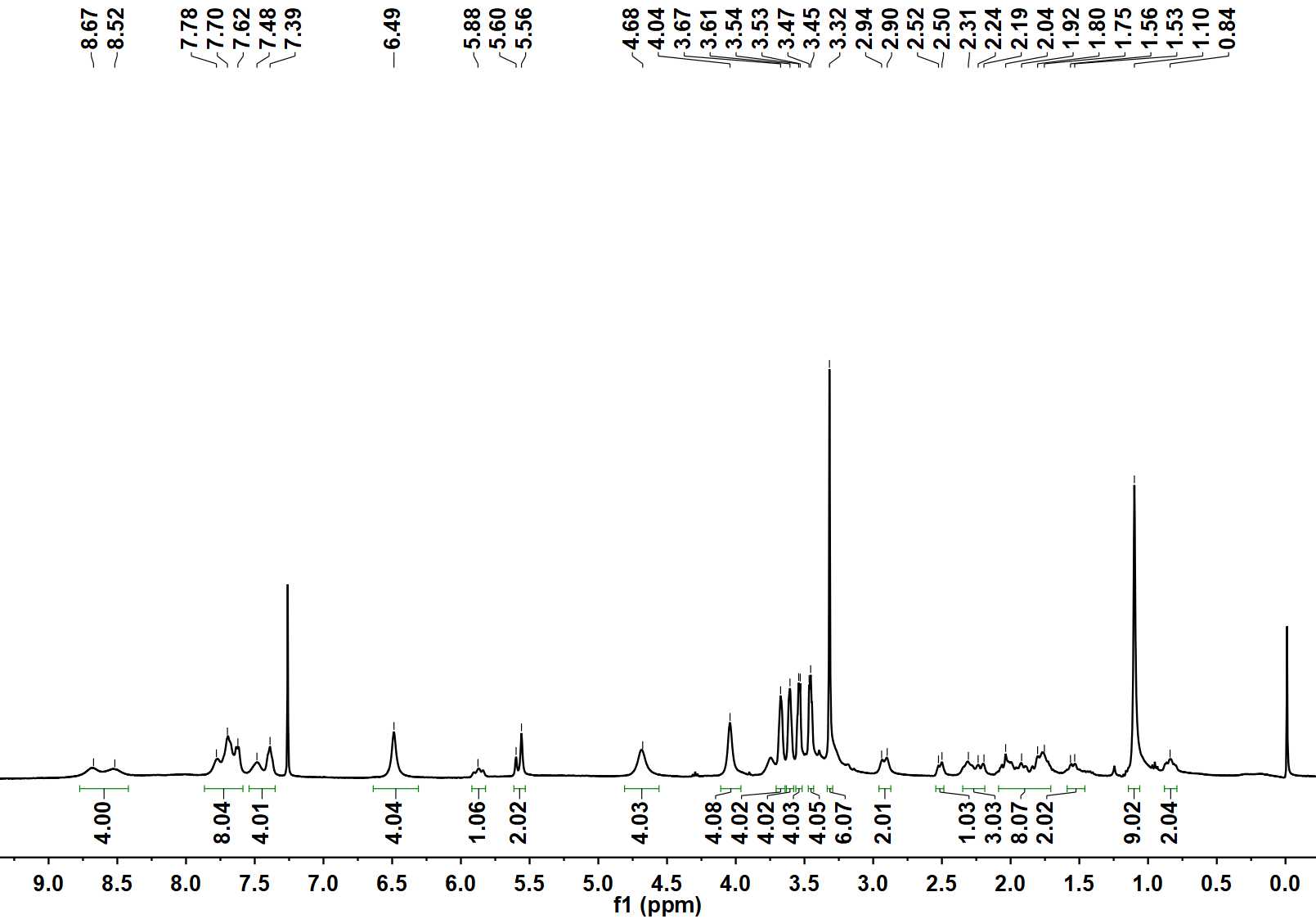


**Figure S23.** ^1^H NMR spectrum of CyTCO in CDCl_3_.


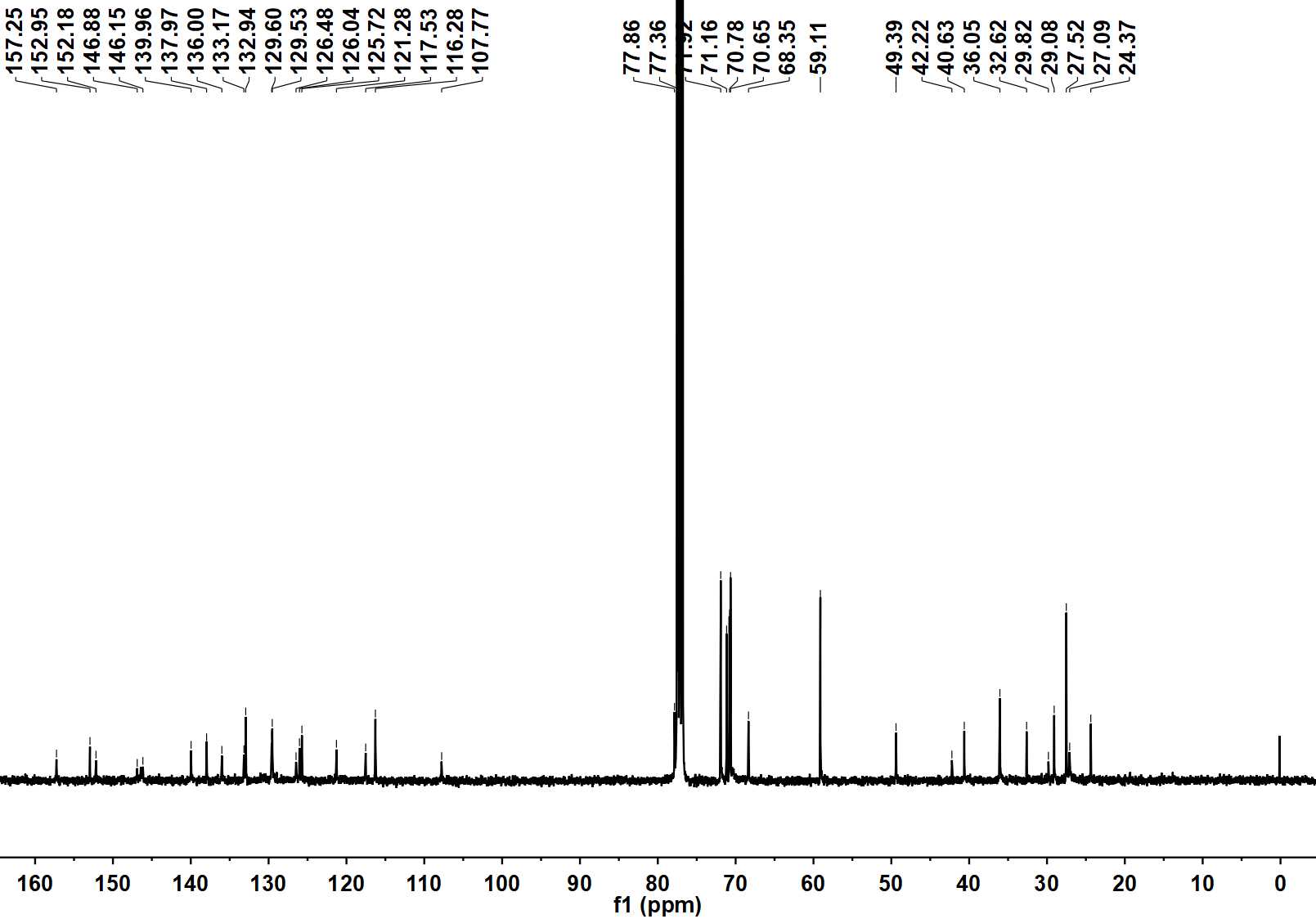


**Figure S24.** ^13^C NMR spectrum of CyTCO in CDCl_3_.


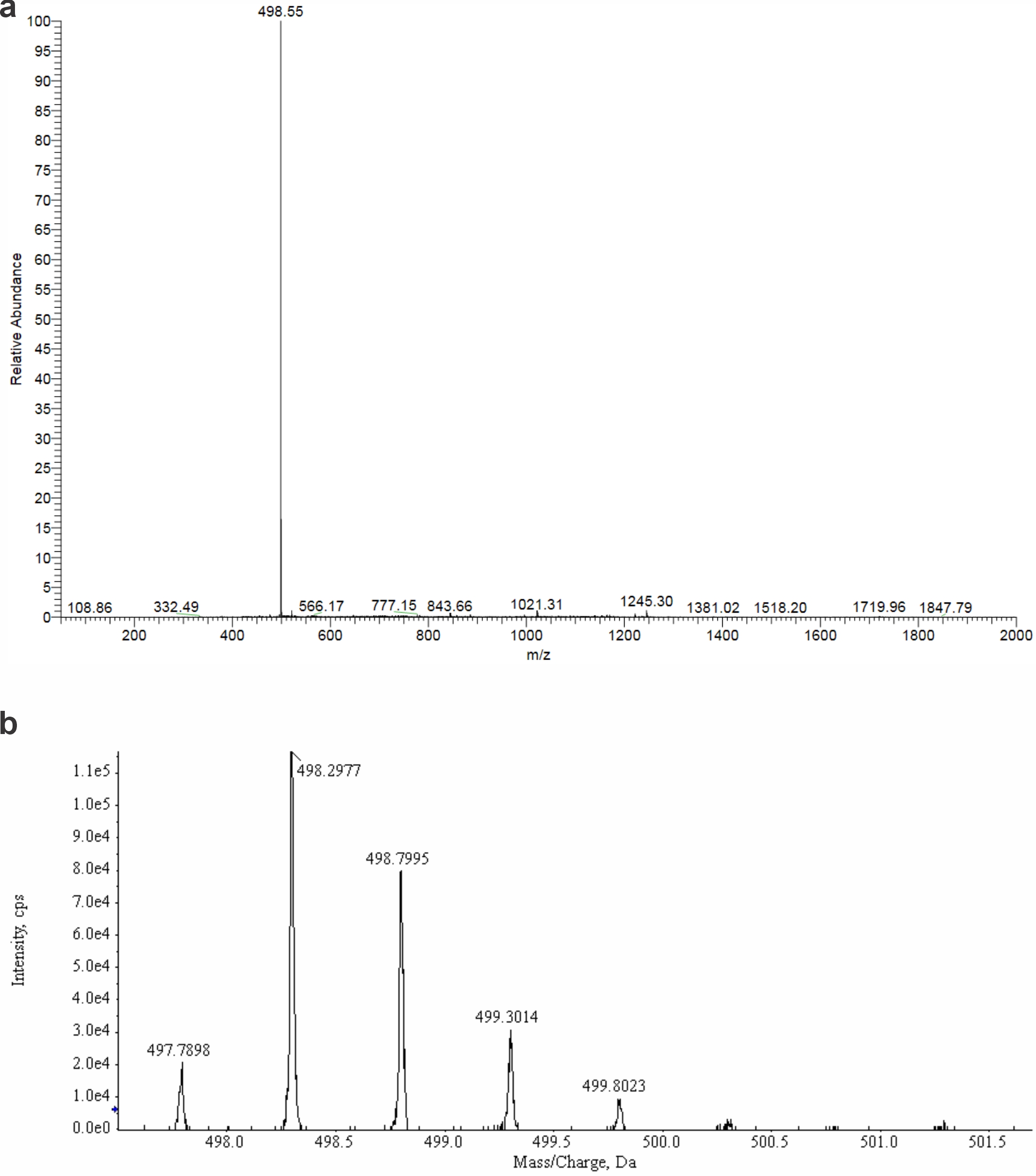


**Figure S25.** **(a)** Liquid chromatography and **(b)** high-resolution mass spectrum of CyTCO.


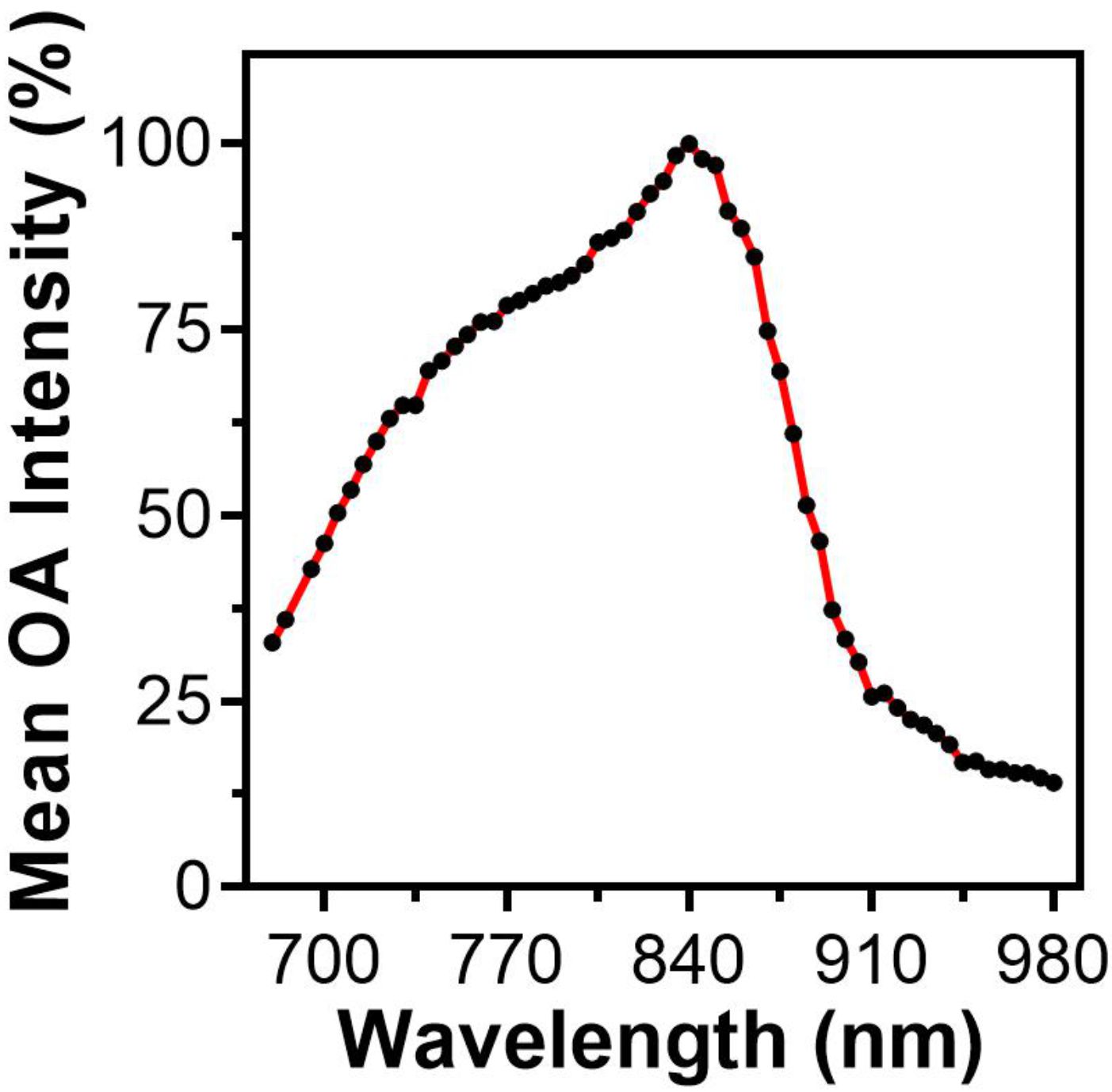


**Figure S26. Optoacoustic spectrum.** The wavelength-dependence of optoacoustic (OA) intensity of CyTCO in the presence of SiaTz in CPBS (10 mM, pH = 6.5) containing 5% DMSO.


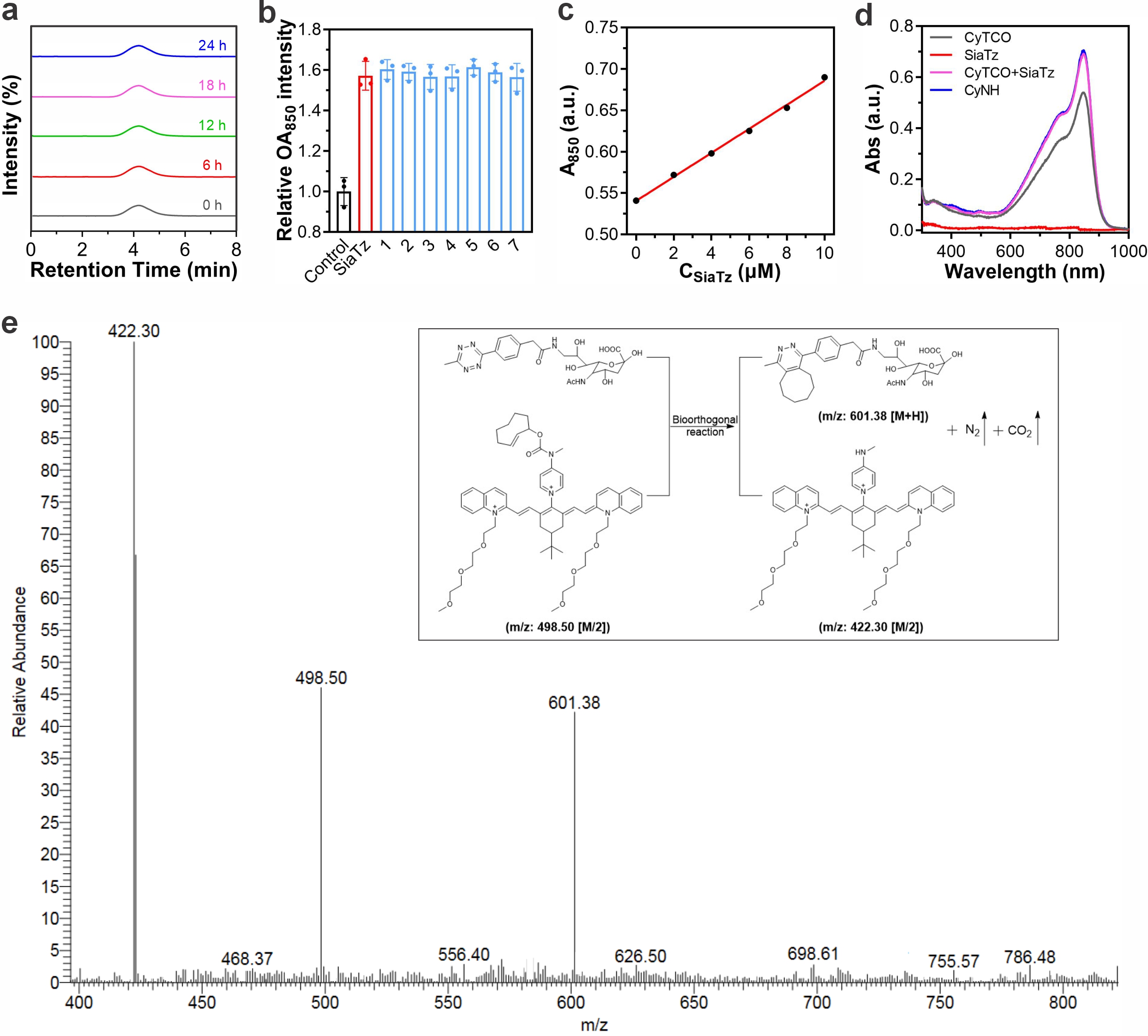


**Figure S27. Responses of CyTCO to SiaTz. (a)** Stability of CyTCO. **(b)** Relative optoacoustic intensity of CyTCO incubated with SiaTz or SiaTz together with other biologically relevant substances in CPBS (10 mM, pH 6.5) containing 5% (v/v) DMSO at 37 °C for 5 min. 1: Na^+^; 2: K+; 3: Ca^2+^; 4: Mg^2+^; 5: Cys; 6: GSH; 7: H_2_O_2_. **(c)** Absorbance of CyTCO at 850 nm as a function of added SiaTz concentration. **(d)** Absorbance spectra of CyTCO, SiaTz, the mixture of CyTCO and SiaTz after 5 mins of reaction, and pure CyNH (the Tz-activated form of CyTCO) in CPBS (10 mM, pH 6.5) containing 5% (v/v) DMSO. [CyTCO] = [SiaTz] = [CyNH] = 10 μM. **(e)** Mass spectrum of CyTCO (10 μM) after incubation with SiaTz (5 μM, half amount of CyTCO) for 5 min. The peak at 422.30 m/z corresponds to CyNH (the activated form of CyTCO), while the peak at 601.38 m/z corresponds to the cyclooctene addition product of SiaTz. As for the peak at 498.50 m/z, it belongs to excess CyTCO.


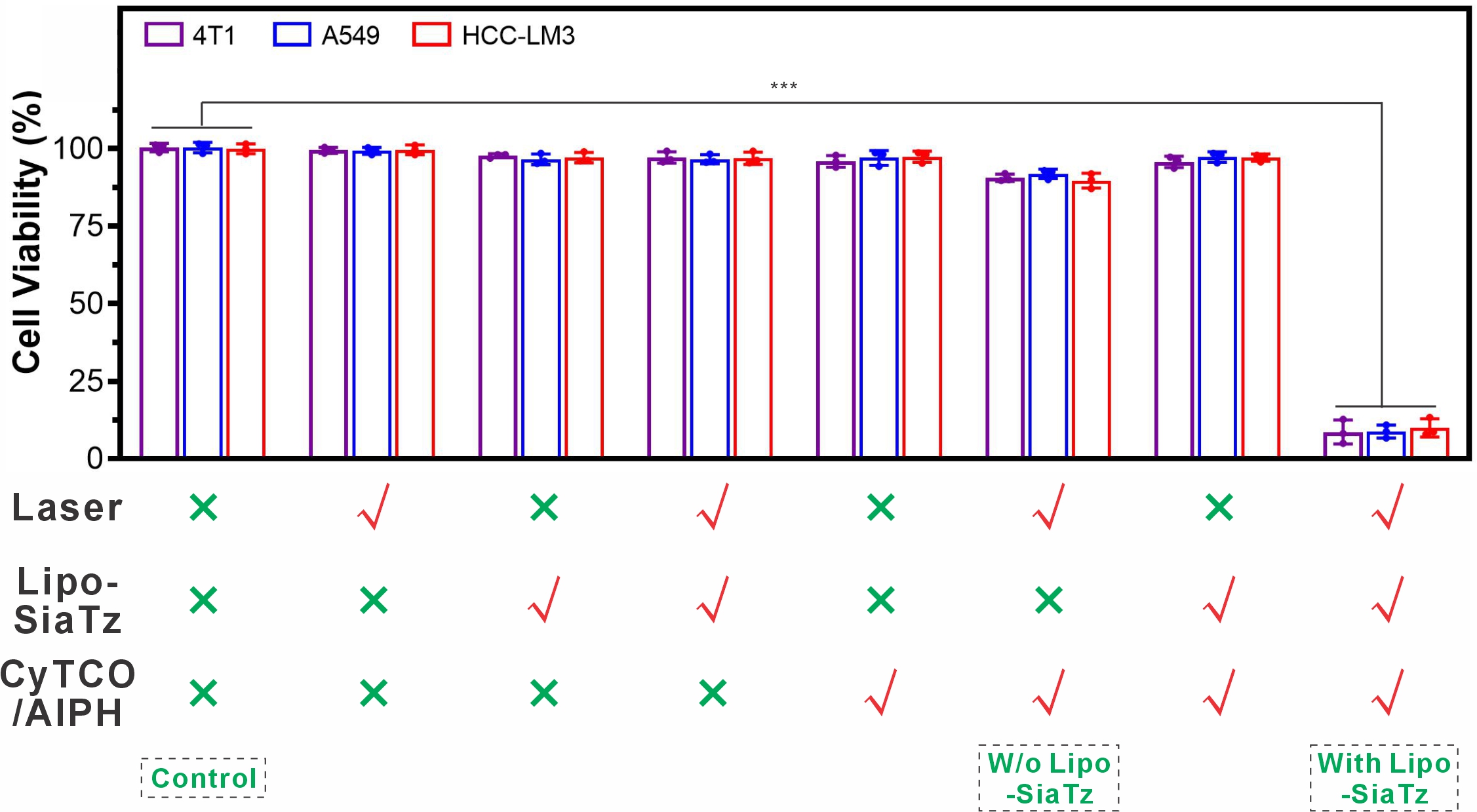


**Figure S28. In vitro apoptosis.** Cytotoxicity of CyTCO/AIPH (CyTCO/AIPH concentration: 10 μM /50 μM) on 4T1, A549 and HCC-LM3 cancer cells pretreated with or without Lipo-SiaTz (equivalent Tz concentration: 50 μM) under 808 nm laser (1 W cm^-2^) irradiation for 5 min (n = 3 independent experiments). As for other groups, the cells were treated according to the corresponding formulations. Data were represented as mean values ± standard deviation. Statistical significance was determined by two-tailed t test. ^***^P < 0.001.


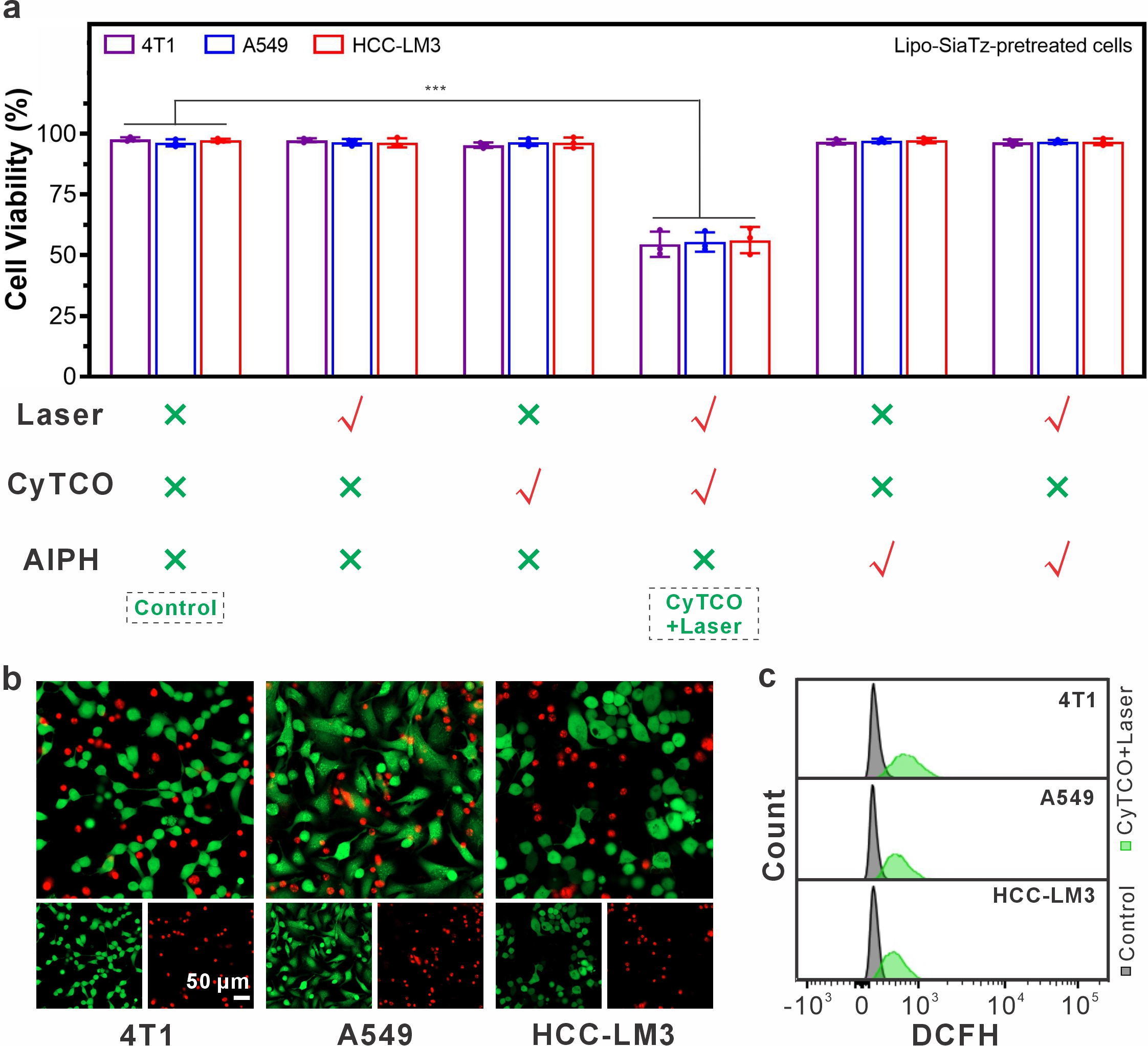


**Figure S29.** **Cytotoxicity of CyTCO. (a)** Cytotoxicity of CyTCO (10 μM) on 4T1, A549 and HCC-LM3 cancer cells pretreated with Lipo-SiaTz (equivalent Tz concentration: 50 μM) under 808 nm laser irradiation (1 W cm^-2^, 5 min) (n = 3 independent experiments). For other groups, the cells were treated according to the indicated formulations. **(b)** CLSM images of Calcein-AM/PI-stained cells which were pretreated with Lipo-SiaTz (equivalent Tz concentration: 50 μM), and then irradiated with an 808 nm laser (1 W cm^-2^, 5 min) after incubation with CyTCO (10 μM). **(c)** Flow cytometric analysis on the free radical levels in Lipo-SiaTz (equivalent Tz concentration: 50 μM)-pretreated cells after incubation with or without CyTCO (10 μM) and then irradiation by a 1 W cm^-2^ 808 nm laser for 5 min. Data were represented as mean values ± standard deviation. Statistical significance was determined by two-tailed t test. ^***^P < 0.001.


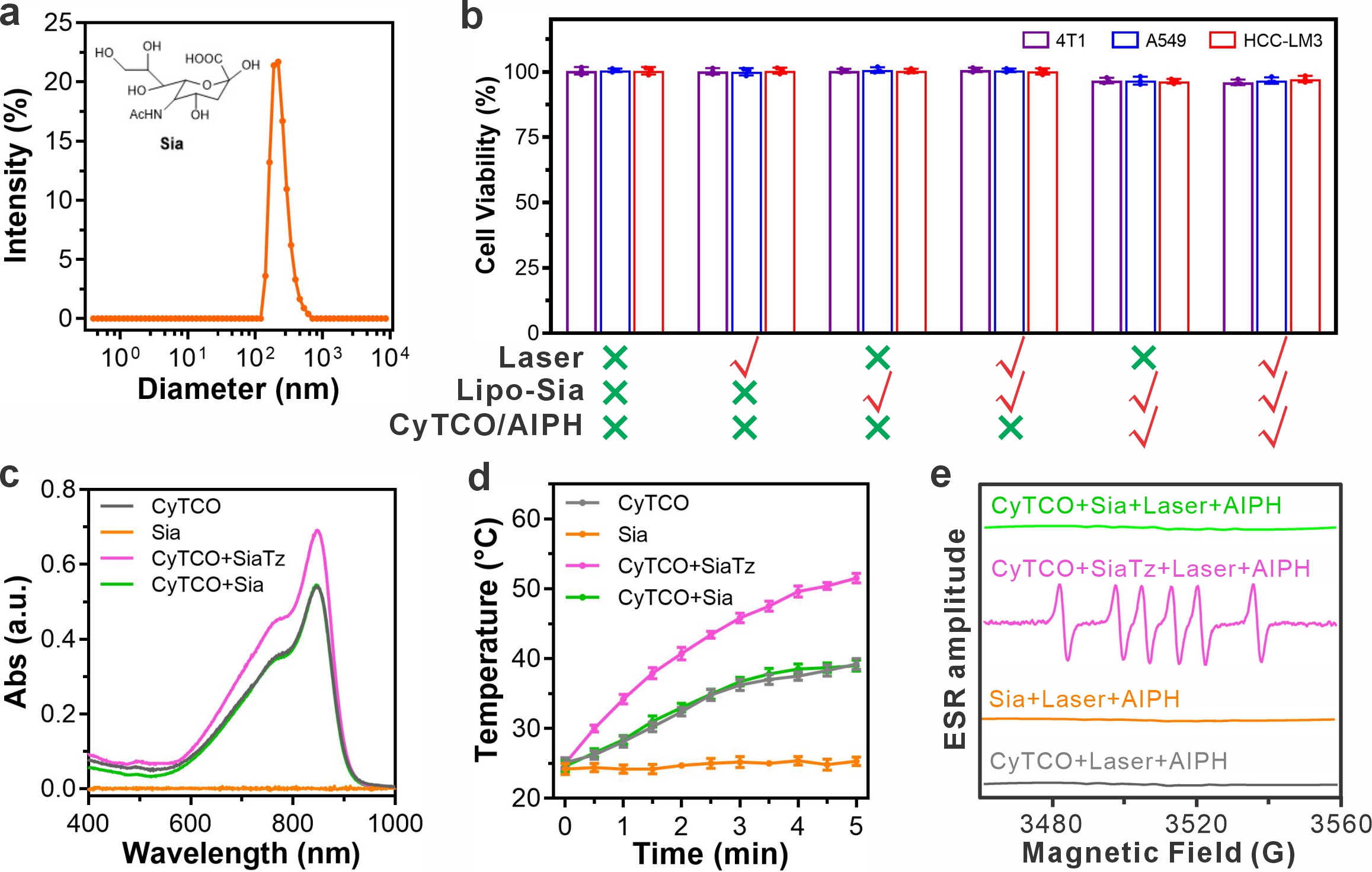


**Figure S30.** **Response of CyTCO toward a control precursor Sia and its converted unnatural target. (a)** Size distribution for Lipo-Sia. Inset: the chemical structure of the control precursor Sia. **(b)** Cytotoxicity of CyTCO/AIPH (CyTCO/AIPH concentration: 10 μM /50 μM) on 4T1, A549 and HCC-LM3 cancer cells pretreated with or without the control liposomal precursor Lipo-Sia (equivalent concentration: 50 μM) under 808 nm laser (1 W cm^-2^) irradiation for 5 min (n = 3 independent experiments). **(c)** Absorbance spectra for CyTCO, Sia, the mixture of CyTCO and SiaTz, as well as the mixture of CyTCO and Sia in CPBS (10 mM, pH 6.5) containing 5% (v/v) DMSO. [CyTCO] = [SiaTz] = [Sia] = 10 μM. **(d)** Photothermal conversion behavior of CyTCO alone, SiaTz alone, the reaction mixture of CyTCO and SiaTz, as well as the reaction mixture of CyTCO and Sia under 808 nm laser (1 W cm^−2^) irradiation. **(e)** ESR spectra of AIPH in the following solutions: CyTCO, Sia, the reaction mixture of SiaTz and CyTCO, as well as the reaction mixture of Sia and CyTCO under 808 nm laser irradiation (1 W cm^−2^) for 5 min. The free radicals produced from AIPH cleavage were captured by DMPO. Data were represented as mean values ± standard deviation.


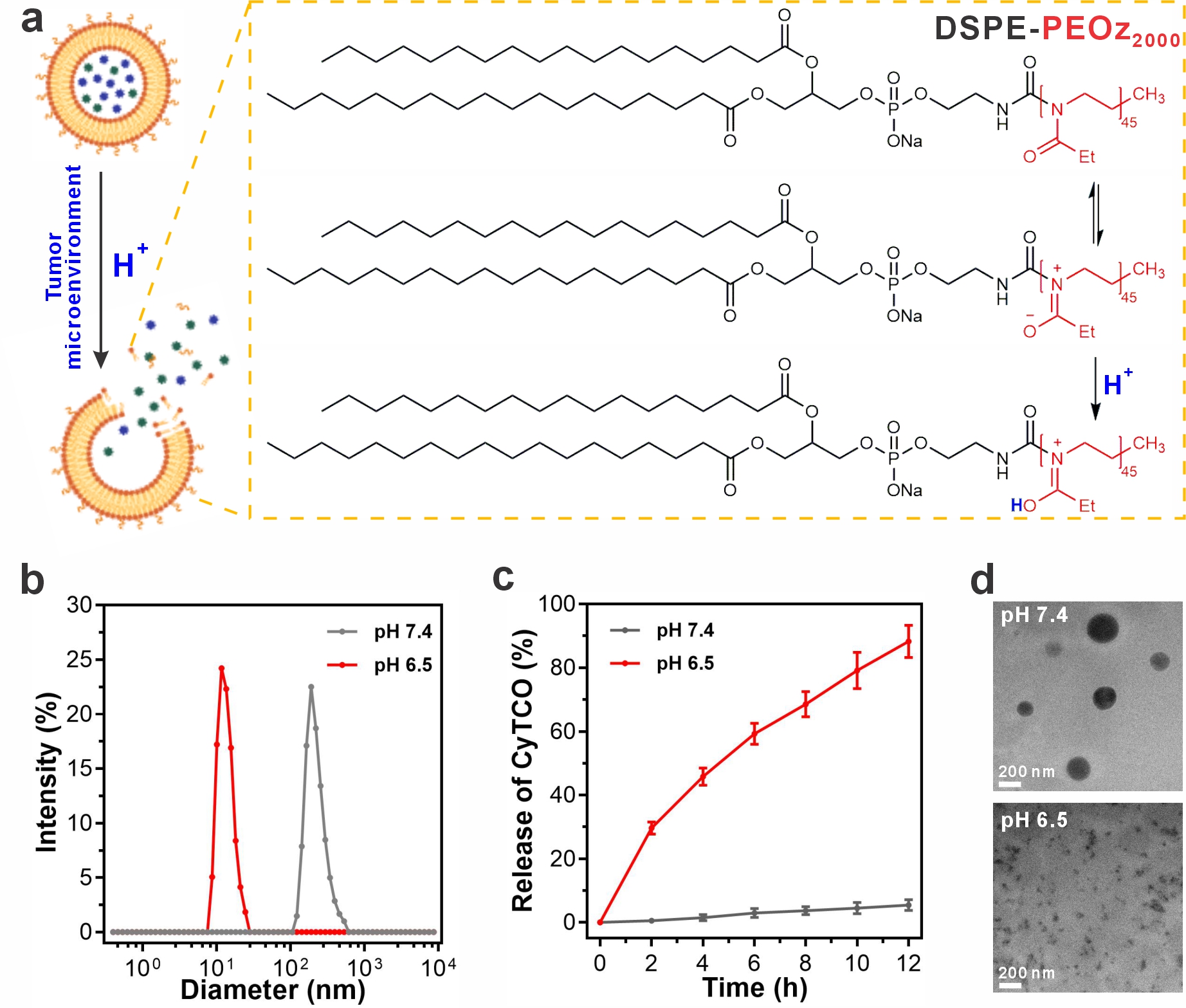


**Figure S31.** **Properties of pHLipo-CyTCO/AIPH.** **(a)** Schematic Illustration of pHLipo-CyTCO/AIPH responding to the acidic tumor microenvironment to release its payloads. The phospholipid DSPE-PEOz2000 in pHLipo-CyTCO/AIPH contains a large number of weakly basic tertiary amine groups, which remains stable at neutral/basic pH. In the acidic tumor microenvironment (pH 6.5), protonation of these amines induces electrostatic repulsion, leading to increased permeability and subsequent liposome disassembly for triggering the release of payloads^[3,4]^. **(b)** Size distribution of pHLipo-CyTCO/AIPH in CPBS at different pH values. **(c)** CyTCO release characteristics of pHLipo-CyTCO/AIPH in CPBS at pH 7.4 and 6.5 during a 12 h incubation. **(d)** Representative TEM images of pHLipo-CyTCO/AIPH in CPBS at pH 7.4 and pH 6.5 after 12 hours of incubation (scale bar: 200 nm).


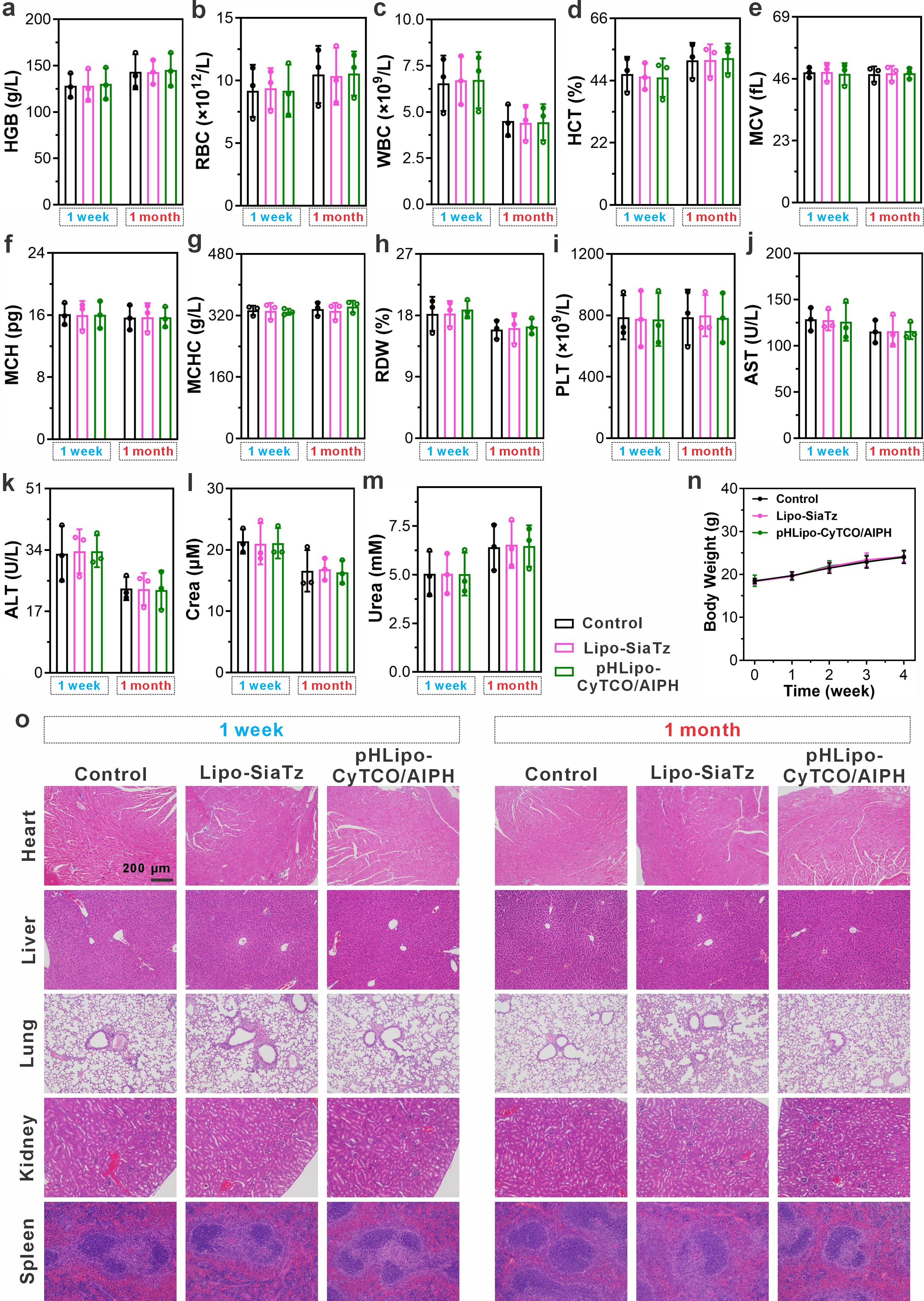


**Figure S32. Biosafety evaluation.** Blood routine indexes **(a-i)** and serum biochemical data (**j-m**) of mice at 1 week or 1 month after injection of PBS (control), Lipo-SiaTz (equivalent SiaTz concentration: 52 mg kg^-1^) or pHLipo-CyTCO/AIPH (equivalent CyTCO/AIPH concentration: 2.4 mg kg^-1^/2.6 mg kg^-1^) (n = 3). HGB: hemoglobin, RBC: red blood cells, WBC: white blood cells, HCT: hematocrit, MCV: mean corpuscular volume, MCH: mean corpuscular hemoglobin, MCHC: mean corpuscular hemoglobin concentration, RDW: red cell distribution width, PLT: platelets, ALT: alanine transaminase, AST: aspartate aminotransferase, Crea: creatinine and Urea: carbamide. **(n)** Time-dependent changes in body weight (n = 5) and **(o)** representative histological sections for main organs of the control mice, and the mice at 1 week or 1 month after administration with Lipo-SiaTz (equivalent SiaTz concentration: 52 mg kg^-1^) or pHLipo-CyTCO/AIPH (equivalent CyTCO/AIPH concentration: 2.4 mg kg^-1^/2.6 mg kg^-1^).


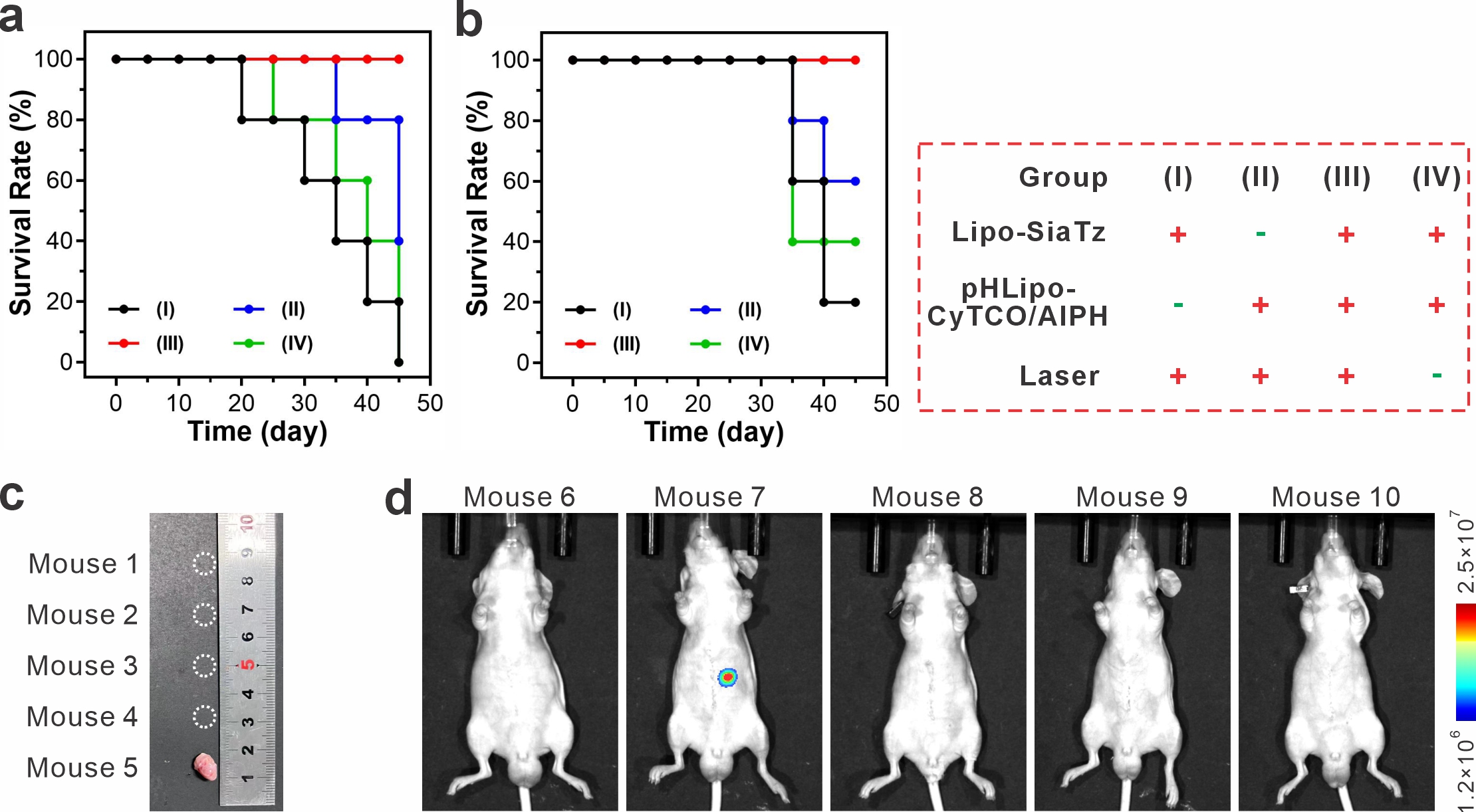


**Figure S33. Assessment of survival rate and tumor recurrence.** Survival rate of mice **(a)** with subcutaneous tumors or **(b)** orthotopic liver tumors in different groups after treatment (n = 5). Tumor recurrence in Group III mice with **(c)** subcutaneous tumors and **(d)** orthotopic liver tumors at Day 60 post-treatment (n = 5). Grouping details: (I) pretreatment with Lipo-SiaTz for 3 consecutive days followed by laser irradiation (no pHLipo-CyTCO/AIPH administration); (II) administration with pHLipo-CyTCO/AIPH followed by laser irradiation (no Lipo-SiaTz pretreatment); (III) pretreatment with Lipo-SiaTz for 3 consecutive days followed by pHLipo-CyTCO/AIPH administration and laser irradiation; (IV) pretreatment with Lipo-SiaTz for 3 consecutive days followed by pHLipo-CyTCO/AIPH administration (no laser irradiation).


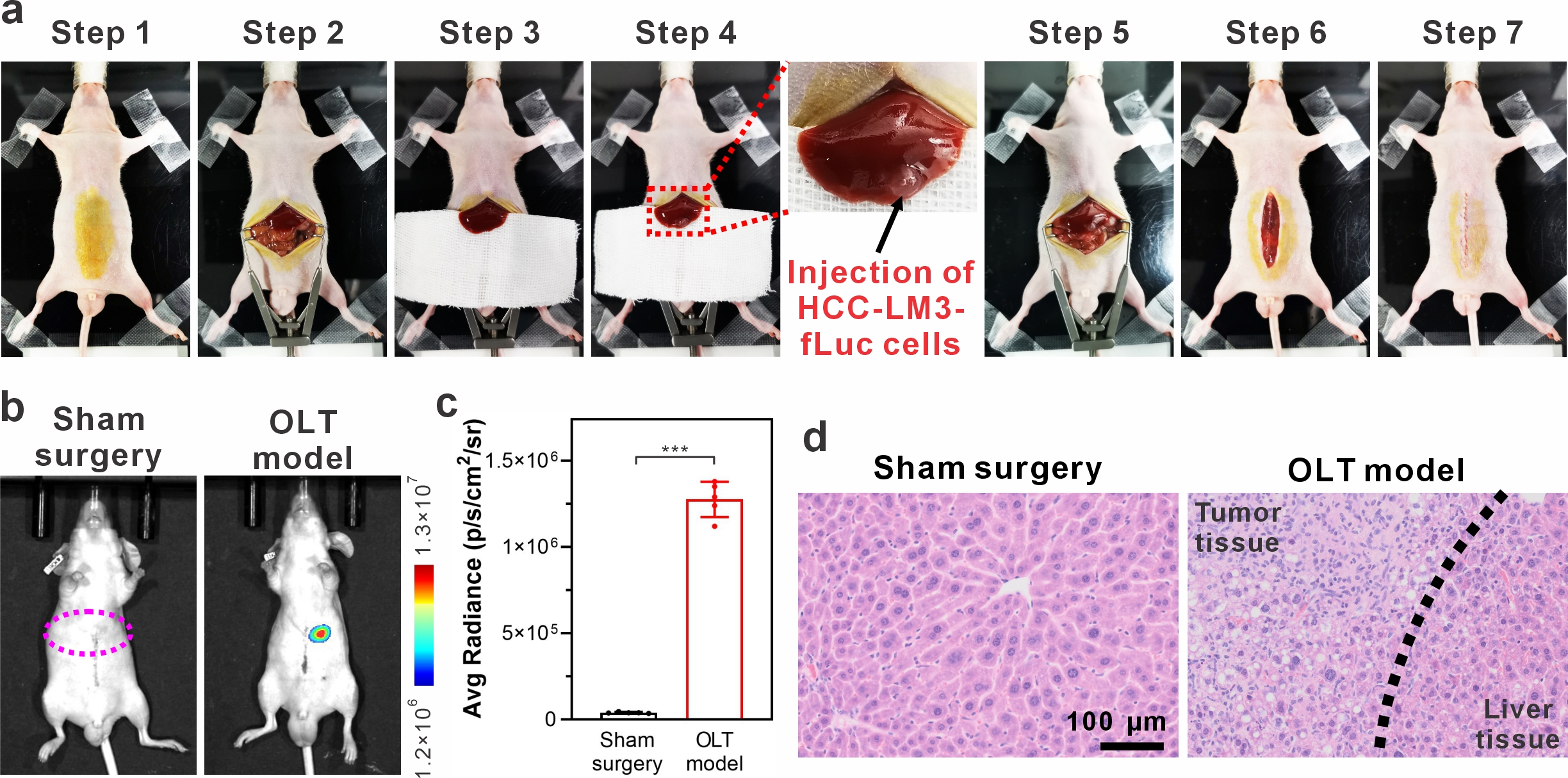


**Figure S34. Establishment of orthotopic liver tumor (OLT) mouse model. (a)** Photographs for the surgical process of orthotopic liver tumor modeling. **(b)** Representative bioluminescence images of mice 7 days after receiving sham surgery or intrahepatic injection of HCC-LM3-fLuc cells. **(c)** Bioluminescence intensity at ROI for the sham-surgery mice and OLT mice (n = 5). The entire liver area of mice was delimited with a pink dotted circle (ROI). **(d)** Typical H&E-stained sections for the liver from the sham-surgery mouse and the OLT mouse. Data were represented as mean values ± standard deviation. Statistical significance was determined by two-tailed t test. ^***^P < 0.001.


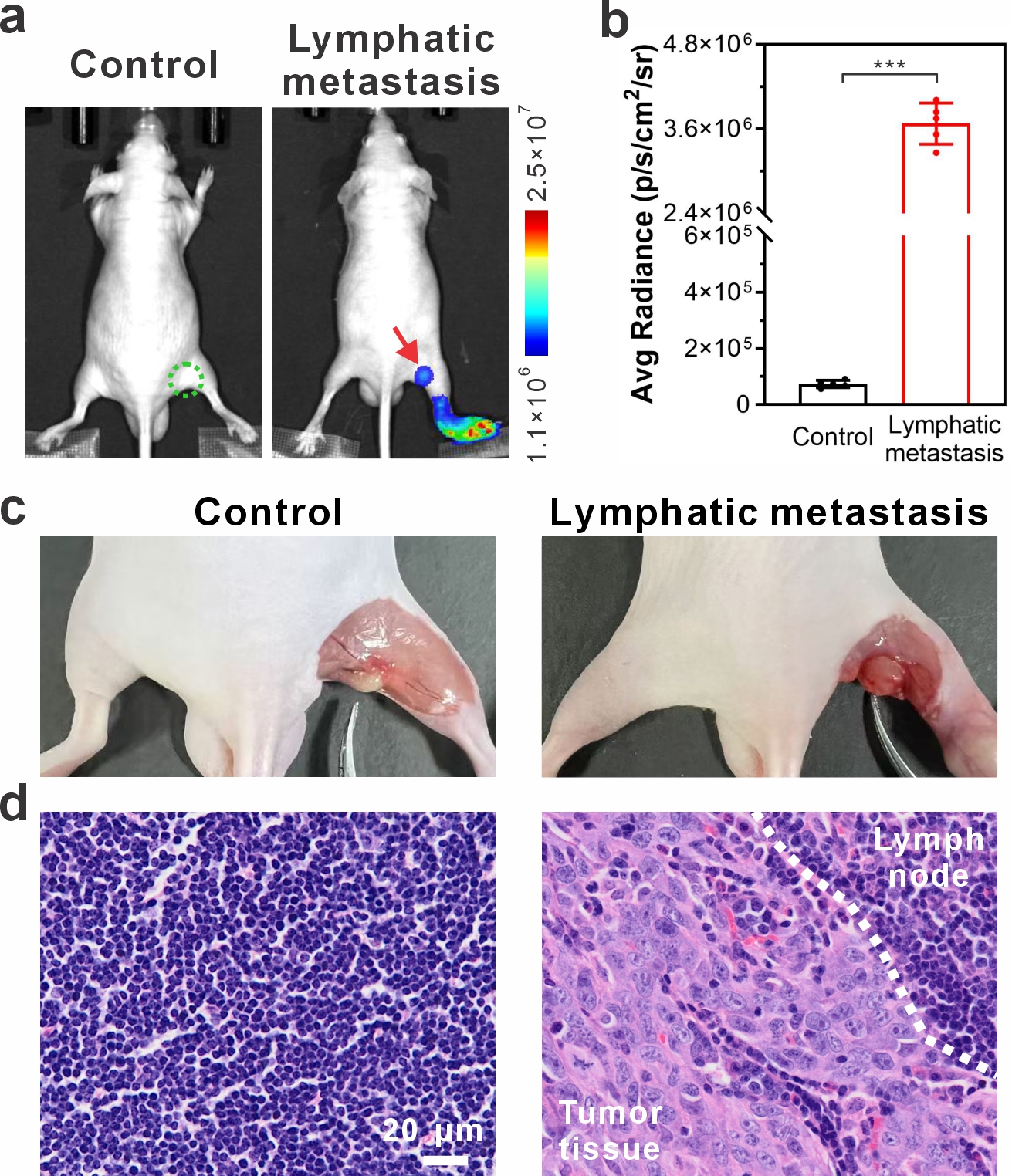


**Figure S35. Establishment of lymphatic metastasis mouse model. (a)** Representative bioluminescence images of mice 21 days after receiving intro-footpad injection of saline (control) or 4T1-fLuc cells. The popliteal lymph node region of mice was delimited with a green dotted circle (ROI). **(b)** Bioluminescence intensity at ROI for the control mice and mice with lymphatic metastasis (n = 5). **(c)** Photos for the popliteal lymph node of the control mouse and the mouse with lymphatic metastasis. **(d)** Typical H&E-stained sections for the popliteal lymph node from the control mouse and the mouse (with lymphatic metastasis) 21 days post intra-footpad inoculation. Data were represented as mean values ± standard deviation. Statistical significance was determined by two-tailed t test. ^***^P < 0.001.


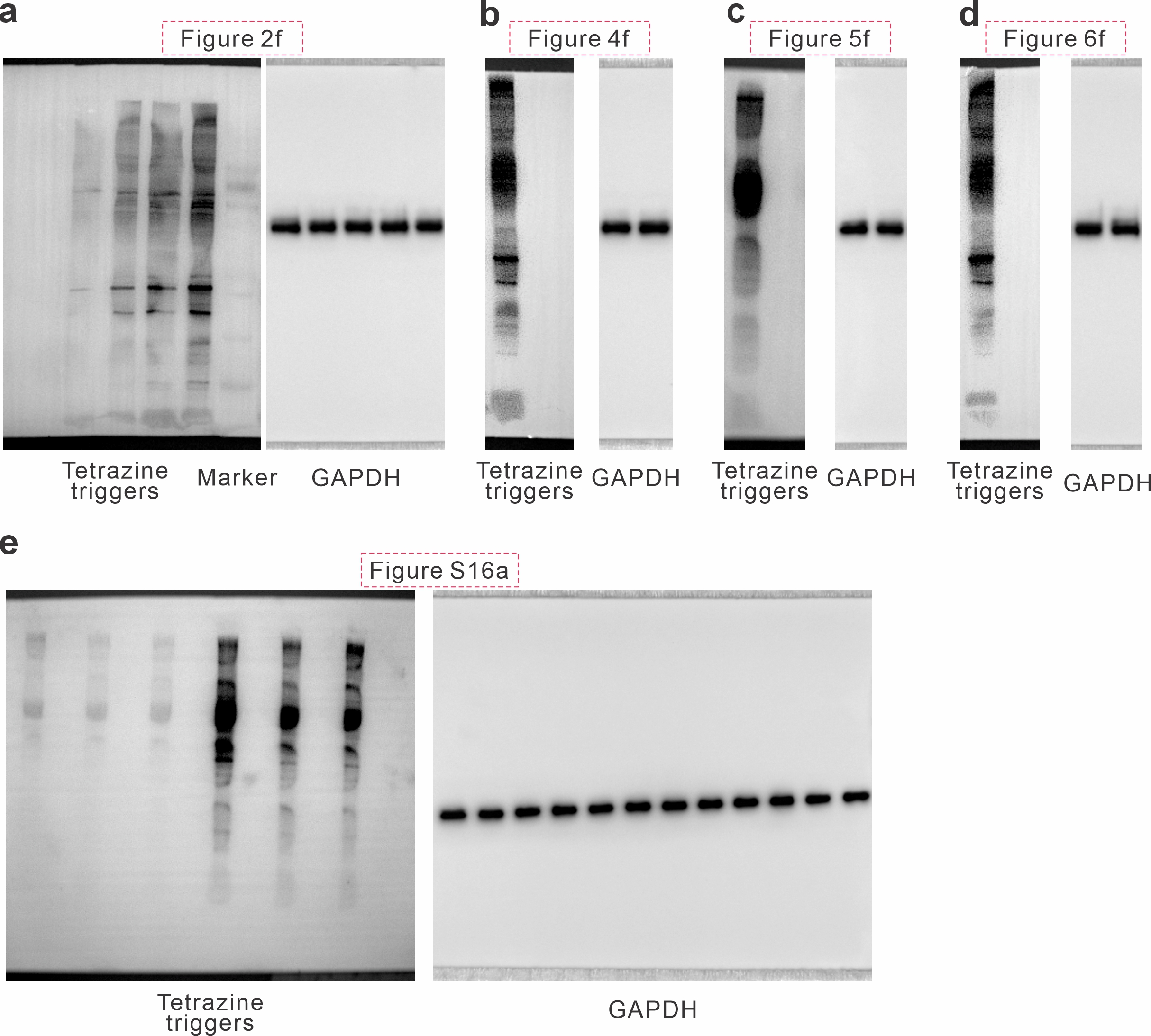


**Figure S36. Full-size blots.** Original blot for **(a)** Figure 2f, **(b)** Figure 4f, **(c)** Figure 5f, **(d)** Figure 6f and **(e)** Figure S16a.

**Table S1.** Comparison of the fabricated unnatural tetrazine triggers with other reported unnatural chemical groups.

| Unnatural groups  in precursors | Complementary groups in theranostic agents | Catalysts | Coupling products | Kinetic values/  Application |
| --- | --- | --- | --- | --- |
| 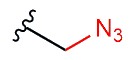  (Azide)^[5]^ | 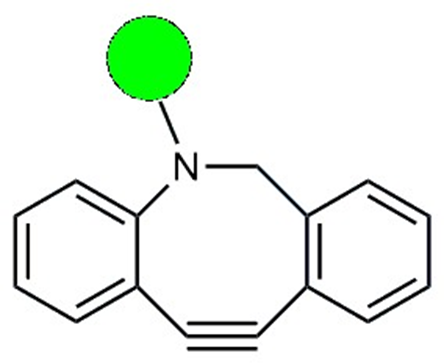  (DBCO) | No | 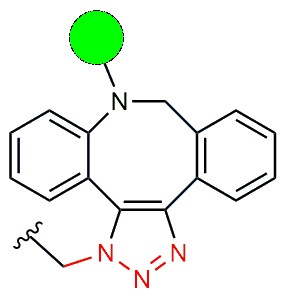 | 0.31-0.96 M^–1^ s^–1^  Widely used in vivo |
| 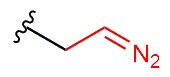  (Diazoalkane)^[6]^ | 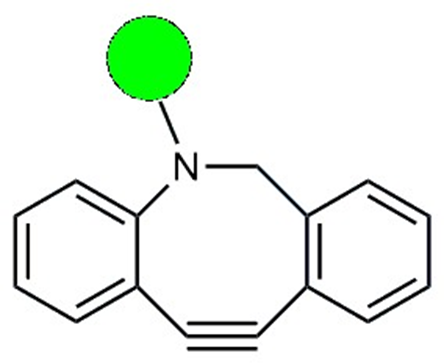  (DBCO) | No | 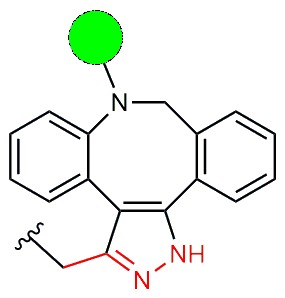 | 0.45 M^–1^ s^–1^  Not attempted in vivo |
| 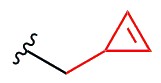  (Cyclopropene)^[7]^ | 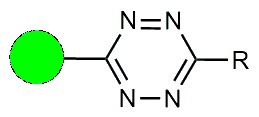  (Tetrazine) | No | 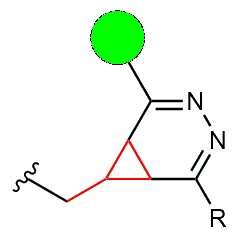 | 0.0004-3.34 M^–1^ s^–1^  Not attempted in vivo |
| 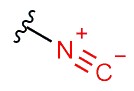  (Isonitrile)^[8]^ | 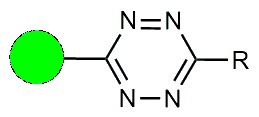  (Tetrazine) | No | 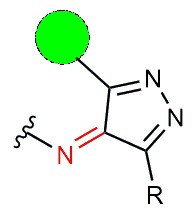 | 0.117-0.38 M^–1^ s^–1^  Not attempted in vivo |
| 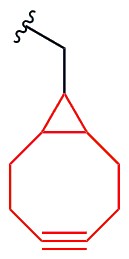  (BCN)^[5]^ | 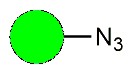  (Azide) | No | 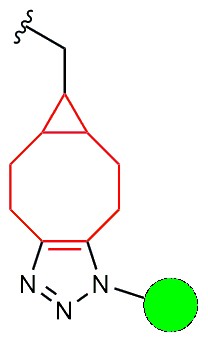 | 0.14 M^–1^ s^–1^  Can be used in vivo |
| 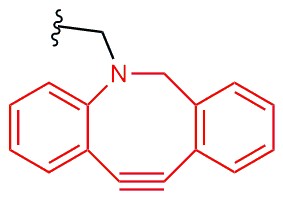  (DBCO)^[5]^ | 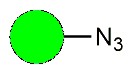  (Azide) | No | 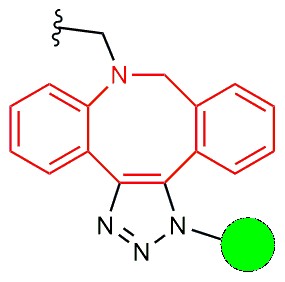 | 0.31-0.96 M^–1^ s^–1^  Can be used in vivo |
| 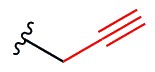  (Alkyne)^[5]^ | 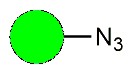  (Azide) | [Cu^+^] | 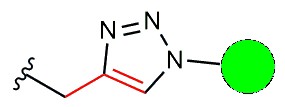 | 10-100 M^–1^ s^–1^  Limited to in vitro use  (Cu^+^ toxicity) |
| 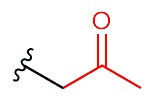  (Ketone)^[9]^ | 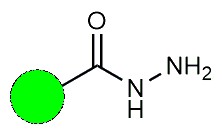  (Hydrazide) | [H^+^] | 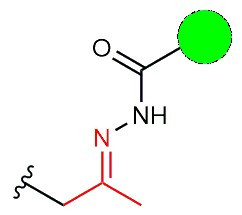 | 0.01 M^–1^ s^–1^  Limited to in vitro use  (Lack specificity) |
| 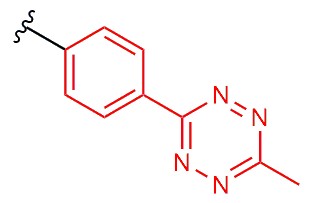  (Tetrazine)  **This work** | 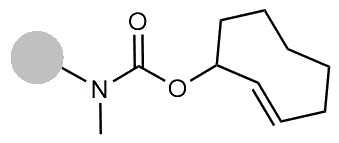  (TCO) | No | 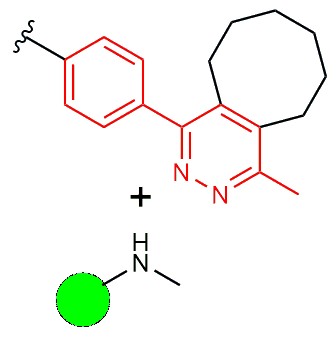 | 1742 M^–1^ s^–1^  Can be used in vivo  (Activatable) |

Note: BCN: bicyclononyne; DBCO: dibenzocyclooctyne; TCO: trans-cyclooctene.

**Table S2.** Loading efficiency (*LE*) and loading capacity (*LC*) of Lipo-SiaTz and pHLipo-CyTCO/AIPH.

| Loaded component | Liposome | *LE* (%) | *LC* (%) |
| --- | --- | --- | --- |
| SiaTz | Lipo-SiaTz | 70.3 | 6.57 |
| ManTz | Lipo-ManTz | 67.4 | 6.67 |
| CyTCO | pHLipo-CyTCO/AIPH | 76.7 | 8.42 |
| AIPH | pHLipo-CyTCO/AIPH | 80.8 | 3.66 |

**Supporting References**

1. J. Yang, Y. Liang, J. Šečkutė, K. N. Houk, N. K. Devaraj, *Chem. Eur. J.* **2014**, *20*, 3365-3375.
2. A. Darko, S. Wallace, O. Dmitrenko, M. M. Machovina, R. A. Mehl, J. W. Chin, J. M. Fox, *Chem. Sci.* **2014**, *5*, 3770-3776.
3. W. Ma, Y. Yang, J. Zhu, W. Jia, T. Zhang, Z. Liu, X. Chen, Y. Lin, *Adv. Mater.* **2022**, *34*, 2109609.
4. D. Wang, Y. Zhou, X. Li, X. Qu, Y. Deng, Z. Wang, C. He, Y. Zou, Y. Jin, Y. Liu, *ACS Appl. Mater. Interface*s **2017**, *9*, 6916-6930.
5. H. Wang, D. J. Mooney, *Nat. Chem.* **2020**, *12*, 1102-1114.
6. L. Josa-Culleré, Y. A. Wainman, K. M. Brindle, F. J. Leeper, *RSC Adv.* **2014**, *4*, 52241-52244.
7. J. Yang, J. Seckute, C. M. Cole, N. K. Devaraj, *Angew. Chem. Int. Ed.* **2012**, *51*, 7476-7479.
8. S. Stairs, A. A. Neves, H. Stöckmann, Y. A. Wainman, H. Ireland-Zecchini, K. M. Brindle, F. J. Leeper, *ChemBioChem* **2013**, *14*, 1063-1067.
9. K. J. Yarema, L. K. Mahal, R. E. Bruehl, E. C. Rodriguez, C. R. Bertozzi, *J. Biol. Chem.* **1998**, *273*, 31168-31179.
